# Structural Basis for a Scaffolding Role of the COM Domain in Nonribosomal Peptide Synthetases

**DOI:** 10.1002/anie.202506621

**Published:** 2025-07-15

**Authors:** Julia Diecker, Benedikt Hermanns, Jennifer Rüschenbaum, René Rasche, Wolfgang Dörner, Alexander Schröder, Daniel Kümmel, Henning D. Mootz

**Affiliations:** ^1^ Institute of Biochemistry, Department of Chemistry and Pharmacy University of Münster Correnstraße 36 48149 Münster Germany

**Keywords:** Conformational changes, Nonribosomal peptide synthetase (NRPS), Peptide biosynthesis, Photo‐crosslinking, Protein dynamics

## Abstract

Nonribosomal peptide synthetases (NRPSs) are multi‐domain enzymes that catalyze the biosynthesis of therapeutically relevant natural products. Efficient peptide synthesis relies on intricate domain interactions, whose underlying principles remain poorly understood. The communication‐mediating (COM) domains facilitate interactions between separate NRPS subunits. For unknown reasons, COM domains co‐occur with epimerization (E) domains, are partially embedded within the adjacent condensation (C) domains and can also be found as internal *cis*‐COM domains. These features set COM domains apart from other docking domains. We present the first crystal structure of a *cis*‐COM domain within an E‐COM‐C domain arrangement from modules 4 and 5 of bacitracin synthetase 3 (BacC). The structure reveals a compactly folded COM domain sandwiched between E and C domains, suggesting a role of the COM domain in orienting these domains for efficient peptidyl carrier protein (PCP) shuttling. Through mutational analyses, dipeptide formation assays, and proximity‐dependent photo‐crosslinking experiments, we investigated both *cis*‐ and *trans*‐COM domains and provide evidence supporting a principal role of COM domains as scaffolds of NRPS architecture. Their function as docking domains may be a secondary consequence of their division into separate donor and acceptor parts.

## Graphical abstract

COM domains are known as docking domains between subunits of nonribosomal peptide synthetases (NRPSs). Here, the first structure of a fused, internal COM domain with its neighboring domains is reported. Structural, biochemical and crosslinking data show that COM domains play an unexpected role in properly positioning epimerization (E) and condensation (C) domains to facilitate shuttling of the peptidyl carrier protein (PCP) and efficient product assembly.

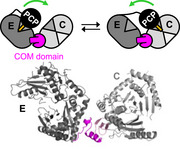

## Introduction

**Figure 1:**
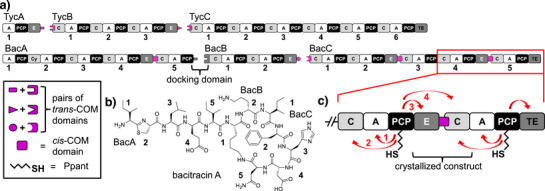
COM domains in NRPSs. a) Shown are the tyrocidine A (top) and bacitracin A (bottom) NRPSs with *trans*‐ and *cis*‐COM domains. b) Structure of bacitracin A. c) Interaction partners of the PCP of the BacC4‐C5 modules are highlighted according to the biosynthetic logic. The BacC4 PCP shuttles between the domains of the crystallized E‐COM‐C construct.

Non‐ribosomal peptide synthetases are multi‐functional enzymes that act as protein templates for the biosynthesis of a plethora of peptide‐based natural products (Figure 1).^[^ ^1^, ^2^ ^]^ They activate amino acid building blocks under consumption of ATP, bind them as thioester on the 4′‐phosphopantetheine (Ppant) moiety of the peptidyl‐carrier protein (PCP; or thiolation domain; T). PCPs then deliver the aminoacyl and growing peptidyl intermediates to the catalytic sites dedicated to peptide bond formation, optional chemical modification like epimerization or *N*‐methylation, and finally product release. The corresponding adenylation (A), PCP, condensation (C), epimerization (E), *N*‐methylation (M) and thioesterase (TE) domains are typically organized as a set of domains in an initiation, elongation or termination module. In linear NRPSs,^[^ ^3^ ^]^ one module is responsible for the incorporation of one amino acid monomer and the number and order of modules determines the product sequence; an arrangement that has enabled the rational reprogramming of NRPSs in combinatorial approaches.^[^ ^4^, ^5^ ^]^ A central mechanistic question is how the domain interplay is facilitated, in particular how the PCPs interact with their various catalytic domain partners, as highlighted for the BacC4 module in Figure 1c.^[^ ^6^, ^7^, ^8^, ^9^, ^10^ ^]^ Recent research in this regard has provided snapshots from crystal and cryo‐EM structures^[^ ^11^, ^12^, ^13^, ^14^, ^15^, ^16^, ^17^, ^18^, ^19^ ^]^ and in‐solution approaches have reported on conformational dynamics.^[^ ^20^, ^21^, ^22^, ^23^, ^24^, ^25^ ^]^

Another important structural aspect pertains to intermolecular interaction of NRPS domains and modules that are located on separate polypeptide chains. Such arrangements are often observed for bacterial NRPSs. For example, the tyrocidine A and bacitracin A synthetases each consist of three enzymes (Figure 1a).^[^ ^26^, ^27^ ^]^ Cognate pairs of docking domains at the respective C‐ and N‐termini provide specific recognition between them. Several types of docking domains have been functionally and structurally characterized in NRPSs and the related polyketide synthases (PKSs),^[^ ^28^, ^29^, ^30^, ^31^, ^32^ ^]^ including the type found between the PCP of the BacA and the C domain of the BacB synthetase (Figure 1a).^[^ ^33^ ^]^

However, the first identified type of docking domains, the so‐called communication‐mediating (COM) domain,^[^ ^34^, ^35^ ^]^ is still insufficiently understood. The donor COM (COM^D^) and acceptor COM domains (COM^A^) are the only known docking domains found at interfacing E and C domains, for example between the tyrocidine TycA/TycB and TycB/TycC^[^ ^26^ ^]^ as well as the bacitracin BacB/BacC subunits (Figure 1a).^[^ ^27^ ^]^ Interestingly, there are also intramolecular *cis*‐COM domains with high sequence homology,^[^ ^36^ ^]^ as for example in BacA and BacC (Figure 1a), suggesting *trans*‐COM domains might have arisen from *cis*‐COM domains in gene splitting events. However, these *cis*‐COM domains have received only little attention so far^[^ ^36^, ^37^, ^38^ ^]^ or were merely regarded as a linker between E and C domains.^[^ ^39^ ^]^

Initial biochemical analyses suggested *trans*‐COM domains were only short interacting peptide sequences at the protein termini.^[^ ^34^, ^35^, ^40^ ^]^ Later, a helix‐hand model was proposed, inspired by an artificial protein‐protein contact observed in the crystal lattice of the SrfA‐C NRPS,^[^ ^11^ ^]^ in which an appended myc‐tag appeared to mimic a C‐terminal helix of the COM^D^ part. Notably, the model suggested that the COM^A^ domain also engages β‐strand and turn elements from the internal, globular part of the C domain to form palm and fingers of the hand motif that embeds the COM^D^ helix. Molecular dynamics (MD) modeling of a *trans*‐COM domain supported the model based on the SrfA‐C structure.^[^ ^37^ ^]^ Photo‐crosslinking and mass spectrometry (MS) mapping studies also corroborated a helix‐hand interaction of an amphipathic helix with a hydrophobic palm and charged finger and hand motifs, but suggested a reversed orientation of the donor COM helix in an helix‐up conformation.^[^ ^36^ ^]^ A structure of a native *trans*‐COM complex has remained elusive^[^ ^37^, ^41^, ^42^ ^]^ until very recently when a crosslinked cryo‐EM structure confirmed the helix‐hand proposal with the helix‐up conformation.^[^ ^18^ ^]^

Swapping and inserting native and artificial docking domains provides an enormous potential for engineering novel NRPSs with designer activity,^[^ ^43^ ^]^ however, this promise has not materialized so far for the COM docking domains. Despite some early successes in COM domain swapping experiments,^[^ ^34^, ^35^ ^]^ subsequent studies yielded only modest and partially conflicting results.^[^ ^40^, ^44^, ^45^, ^46^ ^]^ COM domain swapping will likely be restricted to the junctions between E and C domains, thereby limiting this combinatorial approach to NRPS systems harboring E domains. However, the associated d‐amino acids in the NRP are important contributors to the conformation and bioactivity of NRP products.

We hypothesized that the presence of the *cis*‐COM domains hinted at a more general role of COM domains, beyond intermolecular recognition mediated by *trans*‐COM domains.

In this work, we report the first structure of a *cis*‐COM domain in the context of its flanking E and C domains. The crystal structure revealed a compact COM domain forming extensive contacts with both catalytic domains and suggested that the COM domain favorably orients the catalytic domains for efficient interaction with the common PCP partner. To probe this hypothesis, we biochemically characterized structural mutants of both *cis*‐ and *trans*‐COM domains in peptide formation assays and analyzed conformational changes using a photo‐crosslinking approach. Together, our data support the model of a novel scaffolding role of the COM domain as an essential structural part in the multi‐domain organization of NRPS templates.

## Results and Discussion

### Crystal Structure of a Cis‐COM Domain Sandwiched Between E and C Domains

We aimed to crystallize one of the underexplored *cis*‐COM domains^[^ ^36^ ^]^ (see Figure S1 for a sequence alignment of *trans*‐COM and *cis*‐COM domains). We selected the *cis*‐COM domain between modules 4 and 5 of the BacC subunit including its upstream E and downstream C domains (termed E‐COM‐C; Figure 1). We cloned the corresponding gene fragment encoding a 913 aa sequence following PCR amplification from chromosomal DNA of *Bacillus licheniformis* ATCC10716 and expressed it in *E. coli*.^[^ ^27^ ^]^ Recombinant selenomethionine‐substituted E‐COM‐C was purified by Ni‐NTA and size‐exclusion chromatography, crystalized and its structure determined by SAD phasing at ∼3.3 Å resolution. The structure could be confidentially modeled using computational models as template and the positions of the anomalous signals from selenomethionine as reference points (Figures S2 and S3, Table S1).

**Figure 2:**
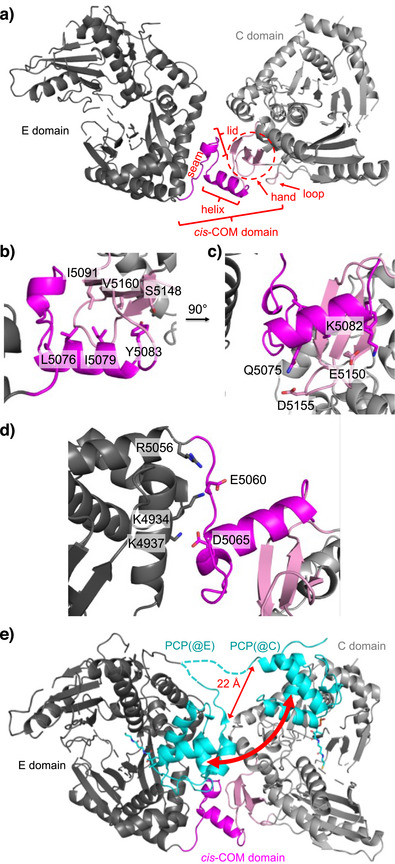
Crystal structure of the *cis*‐COM domain. a) Entire structure of the E‐COM‐C ensemble of BacC4‐C5. b,c) Some key interactions of the helix. d) Some key interactions between E domain and seam/lid. e) Modeling of the BacC4 PCP into the structure at the entry positions to the active sites of both the E domain and the donor site of the C domain, using pdb files 5isx and 6mfz, respectively. The red arrows indicate the PCP movement between the domains and the distance between the PCP's C‐terminal ends in each conformation. The cyan dashed lines in (d) represent proposed linkers to the PCP domain in its two positions, while the grey dashed lines within the E and C domains in (a) and (d) represent unresolved parts of the structure.

In the E‐COM‐C structure, the *cis*‐COM domain appears as a small globular domain sandwiched between and partially overlapping with the E and C domains (Figure 2a). Both the E and C domains adopt their canonical V‐shaped structures.^[^ ^42^, ^47^ ^]^ Regions distant from the *cis*‐COM domain are less well defined and show high b‐factors, indicating flexibility of these parts (Figure S2). Also, several loop regions are not resolved in the electron density map. However, the *cis*‐COM domain is well defined and makes extensive interactions with both E and C domains that result in the formation of rigid interfaces.

The *cis*‐COM domain shows the helix‐hand arrangement with a helix‐up conformation, in agreement with the revised upside‐down helix‐hand model.^[^ ^36^ ^]^ The helix packs with three hydrophobic residues (L5076, I5079 and Y5083), which are conserved across COM domains,^[^ ^36^ ^]^ against the palm of the hand motif, for example, the mostly hydrophobic central residues of three β‐strands (I5091, V5160, S5148; Figure 2b). This part of the structure is consistent with previous work on *trans*‐COM domains.^[^ ^11^, ^34^, ^36^, ^37^ ^]^ The back side of the central helix contains more hydrophilic and charged residues, some of which interact with counterparts on the side of the palm and the finger motifs (e.g., Q5075/D5155, K5082/E5150 and E5152; Figure 2c). Importantly, the structure also reveals the folding of previously uncharacterized parts of the COM sequence that pertain to the stretch upstream of the central helix. Here, a seam segment (aa5056‐5061) follows the last helix of the E domain as a mostly extended polypeptide chain, which then turns to fold into a lid region (aa5062‐5072) that closes off the COM domain structure between the N‐terminal end of the central helix and the first β‐strand of the palm region (Figure 2a). Together, seam, lid and helix represent what was initially defined as the COM^D^ domain in *trans*‐COM domains,^[^ ^34^ ^]^ and all these segments make extensive contacts with the E domain, including salt bridges between E5060 and K4934/R5056 as well as between D5065 and K4937 (Figure 2d) and an interaction between D5074 and R4931. Furthermore, the lid residue D5068 interacts with R5158 of the C domain. Y5066 packs against residues L5076, I5091 and I5088, making up a hydrophobic core with participation from the lid, helix palm and thumb. Finally, a loop element (L5200‐E5203) in the C domain caps off the finger, palm and C‐terminal end of the helix (Figure 2a). In the contiguous polypeptide chain of the *cis*‐COM domain, the C‐terminal end of the helix (part of COM^D^) and the N‐terminal end of the thumb (part of COM^A^) are connected by a peptide bond, similar to a helix‐thumb‐palm arrangement in a PCP‐C didomain of the fungal TgaA NRPS,^[^ ^48^ ^]^ which, however, has no further homology to COM domains. Overall, the COM domain appears to act rather as a rigid connector than as a flexible hinge between its neighboring E and C domains.

### The COM Domain Favorably Positions E and C Domains for Interaction with BacC4 PCP

In the E‐COM‐C structure, the E and C domains are oriented such that the PCP binding sites on both domains face each other (donor position on the C domain). Such an orientation appears functionally relevant because in the NRPS synthesis logic the BacC4 PCP has to visit both domains consecutively and probably dynamically (Figure 1c).^[^ ^23^, ^49^, ^50^ ^]^ While the PCP is tethered via a linker to the E domain, ensuring the proper interaction, it has to reach the donor position of the C domain through the interdomain space resulting from the orientation of the two domains. To examine the possible path of the PCP when shuttling between the two catalytic domains, we modeled it into both binding sites using overlays with a PCP‐E structure showing the epimerization conformation and a PCP‐C structure showing the donor condensation conformation.^[^ ^17^, ^42^ ^]^ The model in Figure 2e shows that the PCP's C‐terminal residue has to translocate by about 22 Å, and additionally, the PCP body has to undergo rotation toward the other catalytic site by about 180°. While representing a significant conformational change, it is important to note that this PCP movement is a necessity to direct the Ppant group to two different domain entry sites. In fact, the path of the PCP appears to be nearly perfectly *minimized* by the observed positioning of E and C domains relative to one another, thereby minimizing entropic penalties and facilitating efficient PCP visits. Together, these considerations provide a rationale for why the COM domain acts as a rigid connector between the E and C domains.

We thus hypothesized a role for the COM domain to properly position the C domain relative to the PCP‐E unit. A different COM structure resulting in a wider opening of the cleft between E and C domains or in a larger twist would necessitate additional movements of the catalytic domain bodies.

### A Functional Dipeptide Formation Assay Reveals Sensitivity of the *cis*‐COM Domain Toward Sequence Changes

**Figure 3:**
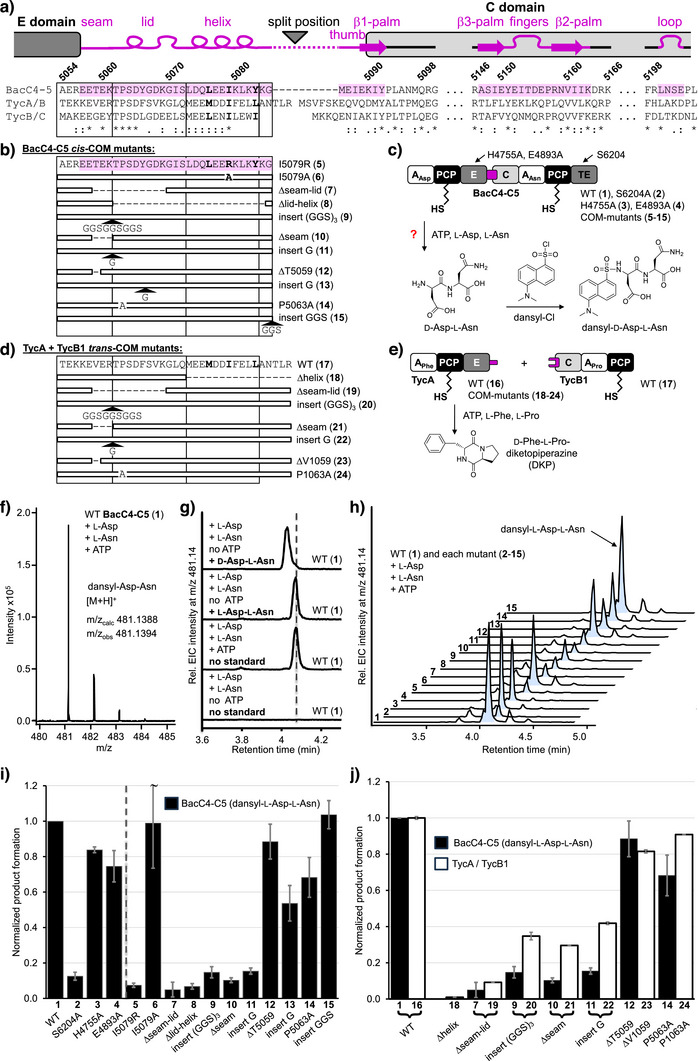
Mutational analysis of *cis*‐ and *trans*‐COM domains. a) Structural features and sequence of the BacC4‐C5 *cis*‐COM domain with sequences of the *trans*‐COM sequences of the TycA/B and TycB/C pairs. b) Schematic representation of investigated BacC4‐C5 as well as its catalytic domain and *cis*‐COM mutants. c) Scheme of the product formation assay with BacC4‐C5. d) Schematic representation of investigated TycA/TycB1 system and its COM^D^ mutants. e) Scheme of the product formation assay with TycA and TycB1. f) Mass spectrum of the dansylated product of BacC4‐C5 (**1**) fitting dansyl‐Asp‐Asn. g) EIC spectra of BacC4‐C5 (**1**) assays including the indicated substrates and spiked chemical standards. h) EIC spectra of product assays with all BacC4‐C5 mutants. i) Diagram of product levels as analyzed in H, normalized to the wildtype (WT) level. Error bars represent the standard deviation of three technical repeats, including protein ppantylation. j) Product formation of equivalent mutants of the *cis*‐COM and *trans*‐COM systems, each normalized to the wildtype (WT) levels. Error bars represent standard deviations of values from BacC4‐C5 and TycA/TycB1 product formation assays with *n* = 3 and *n* = 2, respectively.

To test our hypothesis of a structural role of the COM domain, we aimed for a mutagenesis study as outlined in Figure 3a and b. To this end, we developed a functional assay involving the crystalized *cis*‐COM domain (Figures 1c and 3c). To shortcut the bacitracin biosynthesis leading up to the undecapeptidyl intermediate, we aimed to convert the BacC4 module into an initiation module through deletion of its C domain and upstream modules.^[^ ^22^, ^50^ ^]^ Assuming sufficient substrate tolerances of the catalytic domains with the altered intermediates, the dimodular BacC4‐C5 protein (domain composition A‐PCP‐E‐COM‐C‐A‐PCP‐TE; construct **1**; 2362 aa) should activate and covalently bind l‐Asp, racemize it to the d‐aspartyl thioester and process this moiety along to the BacC5 module, where the resulting d‐Asp‐l‐Asn dipeptide should be cleaved off by the TE domain (Figure 3c).

We prepared recombinant holo‐BacC4‐C5 (**1**) by protein expression in *E. coli* and treatment of the purified protein with Ppant‐transferase Sfp and CoA (Figure S4).^[^ ^51^ ^]^ We confirmed its specific covalent and ATP‐dependent binding of the substrate amino acids by ESI‐MS (Figure S4B). To assay for dipeptide formation, we added ATP (5 mM), l‐Asp (2 mM) and L‐Asn (2 mM). To increase the column retention time of the expected d‐Asp‐l‐Asn product during LC‐MS analysis, we used dansyl chloride to convert all potential peptide products into their dansylated forms (Figure 3c). Indeed, we could detect the expected product peptide mass, and its formation was dependent on the holo‐form of the protein as well as on the presence of all substrates, as expected (Figure 3f and g). Dipeptide formation remained linear over time for about 1 h (Figure S5) at a rate of about 1 molecule per 3 min (Figure S6). A control construct in which the catalytic serine residue in the TE domain was mutated (S6204A; construct **2**) showed only 13% dipeptide production compared to the wildtype (WT) construct **1** (Figure 3h,i; see Figure S4A for all purified Bac4‐C5 constructs), indicating that dipeptide formation occurred along the thiotemplate mechanism, as expected.

However, additional control experiments with two mutant proteins with an inactivated E domain (by mutation of the key catalytic residues H4755A and E4893A in constructs **3** and **4**, respectively) showed only a moderate reduction in product yields to about 80% (Figure 3h,i), suggesting that the participation of the E domain was not crucial. Indeed, comparison with synthetic dipeptide standards revealed that the BacC4‐C5 constructs had formed the diastereomer l‐Asp‐l‐Asn instead of d‐Asp‐l‐Asn (Figure 3g). Based on precedent in the literature, a likely explanation for this observation is that the E domain exhibits a strong substrate preference for its native undecapeptidyl substrate and was not capable to convert the l‐aminoacyl moiety into the d‐stereoisomer.^[^ ^50^ ^]^ In contrast, the BacC5 C domain appeared to have a high tolerance toward the opposite substrate stereochemistry at the donor site,^[^ ^52^, ^53^, ^54^ ^]^ as it accepted the l‐Aspartyl substrate for elongation to the l‐Asp‐l‐Asn dipeptidyl moiety.

We reasoned that, despite these limitations, the dimodular BacC4‐C5 model protein should still be valuable to study our hypothesis. The question whether the COM domain has a structural role to orient the C domain in an optimized distance and orientation relative to the PCP‐E unit should be independent of which aminoacyl thioester epimer is presented to the BacC5 C domain and hence independent of the catalytic participation of the E domain.

We next probed the functional outcome of structural perturbations of the COM domain by insertions, deletions and point mutations of varying severity (Figure 3b). Our rationale was that structural changes were likely to be tolerated if the *cis*‐COM domain mostly serves as a linker to simply ensure covalent connectivity. If, on the other hand, the *cis*‐COM domain is critical in the fine‐tuned positioning of the C domain, dipeptide formation should be sensitive against structural perturbations. ESI‐MS analysis suggested that the aminoacylation activity of the generated mutants was not affected by the perturbations in the COM domain, as expected from the semi‐autonomous character of the catalytic domains (Figure S7).

A destabilizing mutation to a charged residue (I5079R; construct **5**) in the central and hydrophobic helix‐hand interaction interface (Figure 2b) resulted in a drastic product decrease down to 7% compared to the unmutated construct **1**. In contrast, the mutation I5079A (**6**) had no significant effect, likely because it still supported the hydrophobic packing of the helix in the hand (Figure 3b,h,i). In construct **7**, we left the helix‐hand region untouched to ascertain its folding but deleted a part of the seam and most of the lid, to perturb the positioning of E and C domains. This structural change led to a massive drop in product formation to 5%. Similarly, deletion of the lid and the helix in construct **8** resulted in only 7% product formation (Figure 3b,h,i). Together, these findings clearly showed that the COM domain is more than a simple covalent linker.

We then introduced more subtle alterations in the COM sequence that had the potential to still support the overall positioning of the C domain relative to the E domain. Inserting a (GGS)_3_ linker in front of the lid (**9**) might leave the COM fold intact but could increase the distance between E and C domains (Figure 3b). However, dipeptide product yields of only 15% suggested a significant functional impact or another structural consequence (Figure 3h,i). Deletion of three amino acids T5059 to K5061 (**10**) within the seam abrogated dipeptide formation to ≤15%, just like the insertion of a single glycine residue (**11**).

We also observed less pronounced but still significant impacts with other subtle pertubations: Deletion of a single residue in the seam (ΔT5059; **12**) or insertion of a single glycine residue between lid and helix (**13**), resulted in 88% and 54% product yield, respectively. Furthermore, mutation of the reasonably well conserved proline in the lid in mutant P5063A (**14**) reduced yields to 68% (Figure 3b,h,i). Together, these findings indicated a high overall sensitivity of the E‐COM‐C interface toward structural perturbations.

Only the insertion of the flexible linker sequence GGS at the end of the helix (**15**) showed no discernible loss of product yield (Figure 3b,h,i). This position corresponds to the split site in *trans*‐COM domains according to multiple sequence alignments (Figures 3a and S1). The observed tolerance is therefore not surprising and further supports the idea that *trans*‐COM domains can evolve from *cis*‐COM domains. In summary, the mutational analysis corroborated our model of an important structural role of the COM domain.

### Dipeptide formation and proximity‐dependent chemical crosslinking in *trans*‐COM assay confirms scaffolding role of COM fold

We next asked whether the structural role of *cis*‐COM domains extends to *trans*‐COM domains. We chose the first two modules from the tyrocidine biosynthesis (Figure 1a), namely l‐Phe‐activating TycA (module composition A‐PCP‐E‐COM^D^) and the l‐Pro activating TycB1 (COM^A^‐C‐A‐PCP) module taken from a truncated TycB enzyme (constructs **16** and **17**, Figure 3a, d, e).^[^ ^26^ ^]^ The d‐Phe‐l‐Pro dipeptide formed as thioester on the TycB1 autocatalytically cleaves itself off the template by cyclization to d‐Phe‐l‐Pro‐diketopiperazine (DKP), thereby leading to product turnover and enabling sensitive readout of the enzymatic activity.^[^ ^36^, ^55^ ^]^ A negative control construct of TycA with the COM^D^ helix deleted (**18**) showed completely abrogated product formation, consistent with previous reports (Figures 3d,h,i and S8).^[^ ^34^, ^36^, ^56^ ^]^

We introduced most of the analogous deletions, insertions and mutations into seam and lid regions of the TycA COM^D^ domain as described above for the BacC4‐5 *cis*‐COM domain (Figure 3d). The mutant enzymes were unaffected in their aminoacylation activity (Figure S9) and were then assayed for dipeptide formation with TycB1 (Figures 3e and S8). Notably, the structural changes overall had very similar dramatic impacts on dipeptide formation in the *trans*‐COM system. Specifically, the deletion of a seam‐lid part (ΔV1059 to K1069; **19**), the insertion of a flexible (GGS)_3_ linker (between R1061 and T1062; **20**) or of a single glycine (**22**) between seam and lid, as well as the deletion of the last three residues of the seam (V1059 to R1061) (**21**) led to significant reductions in the d‐Phe‐l‐Pro‐DKP production down to 9, 35, 42, and 30%, respectively (Figure 3d,h,i). Better tolerated were only the deletion of the single V1059 in the seam region (**23**) as well as the P1063A mutation in the lid (**24**), again consistent with the relative trends observed for the *cis*‐COM domain of BacC4‐C5 (Figure 3d,h,i). Collectively, these findings corroborated the idea of a high structural similarity between *cis*‐ and *trans*‐COM domains and of a common scaffolding role.

**Figure 4:**
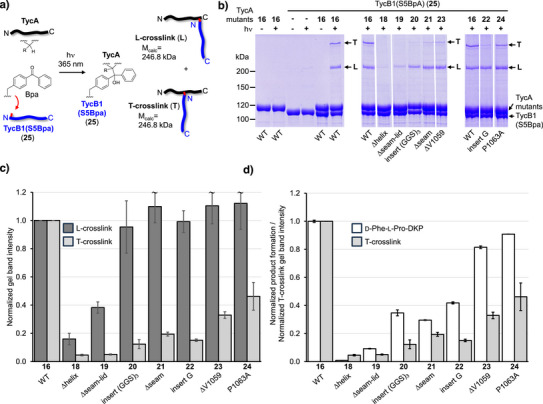
Photo‐activated crosslinking analysis. The indicated COM^D^ mutants of TycA were combined with TycB1(S5Bpa). a) Scheme of the crosslinking reaction resulting in nearly linear (L‐form) and branched (T‐form) polypeptide backbone connectivities of identical mass but different SDS‐PAGE migration behavior. b) Analysis of photo‐crosslink products on Coomassie brilliant blue stained SDS‐PAGE gels. Shown are lanes from three different gels, which were analyzed in relation to the wildtype TycA control (with **16**) present on each gel. c) Diagram of L‐ and T‐crosslink intensities from experiments shown in b) analyzed by densitometry and normalized to the wildtype TycA control. d) Comparison of T‐crosslink intensities shown in c) with d‐Phe‐l‐Pro‐DKP yields as shown in Figure 3j, each normalized to the wildtype (WT) levels. Error bars of crosslink data represent the standard deviations of two technical repeats.

To collect direct evidence for the hypothesis that the COM domain positions the C domain relative to the PCP‐E unit, we turned to conformationally sensitive chemical crosslinking. Proximity‐dependent photo‐activated crosslinking is useful to probe the interaction between two molecules or proteins.^[^ ^57^, ^58^ ^]^ We previously reported the incorporation of the genetically encoded photo‐crosslinking amino acid Bpa^[^ ^57^, ^59^, ^60^ ^]^ into the GrsA/TycB1 and TycA/TycB1 pairs to investigate proximity parameters in the *trans*‐COM domains.^[^ ^36^, ^56^ ^]^ Importantly, Bpa incorporated at Ser5 in the COM^A^ thumb region of TycB1 (TycB1(S5Bpa)) (**25**) results in two crosslink types into different regions of TycA (**16**), one reflecting a direct COM interface interaction, the other a conformation‐dependent domain encounter (Figures 4a and S10).^[^ ^56^ ^]^ The protein band slightly above the 200 kDa marker on an SDS‐PAGE gel corresponds to a crosslink to TycA residue(s) within the COM^D^ helix (calculated weight of a TycA‐TycB1 crosslink is 246 808 kDa; Figures 4a,b and S10 and S11). This crosslink, termed L‐crosslink, results from the helix‐thumb contact of the COM^D^/COM^A^ interaction, which is confirmed by our new E‐COM‐C structure. The second crosslink, termed T‐crosslink, migrates at the upper end of the SDS gel (6% polyacrylamide; apparent molecular weight of 300–400 kDa; Figure 4a,b)^[^ ^56^ ^]^ and connects Ser5Bpa of the thumb with a region of the functionally unrelated small A domain subunit (A^C^) of TycA (Figures S10 and S11). This crosslink captures a close proximity brought about by the overall 3‐D multi‐domain arrangement (Figure S10B).^[^ ^56^ ^]^ The aberrantly slow migration in the SDS‐PAGE gel stems from the branched connectivity of the two polypeptide chains in T‐shape (Figure 4a).^[^ ^56^ ^]^ Here, we reasoned that the conformationally dependent T‐crosslink should also report on the COM‐mediated positioning of the C domain relative to the PCP‐E unit, because the A^C^ domain is directly linked to the PCP, while the thumb region of the COM domain with Ser5Bpa sits on the body of the C domain (Figure S10). A perturbed COM structure should thus affect the proximity between the A^C^ domain and the thumb region. Notably, our crystal structure is consistent with this possible structural arrangement, because the residue corresponding to Ser5 in the COM^A^ of TycB1 faces the domain interspace accessible to the PCP and A^C^ domains.

**Figure 5:**
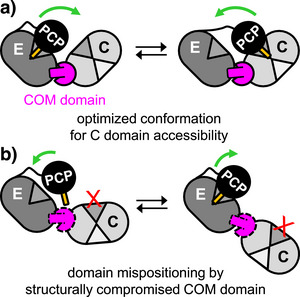
Model for structural role of the COM domain. a) Proper positioning of the C domain relative to the PCP‐E unit. b) As a consequence of a structurally altered COM domain, PCP visits to the C domain are impaired.

The negative control mutant of TycA with the COM^D^ helix deleted (**18**) showed a nearly complete loss of both L‐ and T‐crosslinks with TycB1(S5Bpa), consistent with a lack of association between the two proteins (Figure 4b,c). The seam and lid deletion mutant **19** showed a significant reduction in the L‐crosslink (down to 38% compared to WT TycA), together with a virtually complete disappearance of the T‐crosslink (5%) (Figure 4b,c). Since the diminished L‐crosslink is a clear indication of an impaired intermolecular association with TycB1, it is possible that the observed reduction in the dipeptide product formation assay to 9% is also a result of the reduced affinity toward TycB1, prohibiting further structural conclusions. In contrast, analysis of the remaining internal TycA mutants in the COM^D^ region (**20**, **21**, **22**, **23** and **24**) showed that they all formed nearly unchanged L‐crosslink levels compared to TycA WT (Figure 4b,c). These observations suggested that the structural alterations in their COM^D^ domains did not strongly affect the association with the COM^A^ domain of TycB1, likely because their helix and lid regions were left basically intact. However, the formation of their T‐crosslinks was strongly reduced (Figure 4b,c). Interestingly, the remaining levels of these T‐crosslinks showed a correlation with the levels of dipeptide formation observed for the same mutants. In the first group of mutants (**20**, **21** and **22**) T‐crosslink levels dropped to 12%–19% relative to WT TycA (**16**) (Figure 4b,c) and these proteins exhibited only 30%–42% of dipeptide formation with TycB1 (Figure 4d). On the other hand, a second group of mutants (**23** and **24**) retained relatively higher T‐crosslink yields of 33% and 46% (Figure 4b,c), which correlated with significantly higher dipeptide formation levels of 82% and 91%, respectively (Figure 4d). Thus, these observations established a link between the domain arrangement in 3‐D space and the dipeptide formation activity. Since the latter requires PCP interaction with the downstream C domain across the COM interface, these data provide direct evidence to support our model of the COM domain's structural role for the conformational alignment in the multi‐domain ensemble. Perturbing the structure of the COM domain has the potential to misposition the connected C domain relative to the PCP‐E unit, resulting in impaired PCP interaction with the C domain and reduced peptide elongation activity (Figure 5).

## Conclusion

Multi‐functional NRPSs and other related multi‐domain enzymes like polyketide and fatty acid synthases^[^ ^61^ ^]^ have to coordinate the interaction of the central carrier protein with various catalytic domains to allow for a stepwise and directional product assembly.^[^ ^6^, ^7^, ^8^, ^9^ ^]^ Recent work on NRPS conformations in solution suggests they can adopt a dynamic mixture of interconverting conformations, with the PCP shuttling between catalytic domains in finely graded fashion.^[^ ^20^, ^21^, ^22^, ^23^, ^24^ ^]^ The A domain is one driving force for conformational changes with its ATP‐driven cycle of amino acid adenylation and thiolation reactions.^[^ ^62^ ^]^ Furthermore, the chemical state of the Ppant terminus (e.g., free thiol group, aminoacyl or peptidyl thioester) influences the relative population of different possible conformations as it determines for which catalytic domain(s) it serves as the fitting substrate or acts by product inhibition.^[^ ^20^ ^]^ Preferred adoption of a particular conformation helps to prime the enzyme for a productive PCP encounter with a catalytic domain, which together with the catalytic properties of the latter will decide on the rate of catalyzing the next step.

We here show that the COM domain, both as *cis*‐COM and as split *trans*‐COM domain, plays an important scaffolding role in addition to its previously described function as a *trans*‐docking domain. The resulting domain positioning appears as another factor affecting the proper conformations of an NRPS assembly line to optimize product throughput. The importance of this aspect of COM domains is reflected by the observation that, to the best of our knowledge, a *cis*‐COM or *trans*‐COM domain is always found when an E domain is integrated into the NRPS assembly line (note that another type of epimerization domain exists that is inserted differently).^[^ ^63^ ^]^ In contrast, other docking domain types are only found as *trans*‐forms in split NRPS chains, where they reside as additional tag sequences at the C‐ and N‐termini. The scaffolding role thus explains the strict co‐occurrence of the COM domain with E domains. We propose that all COM domains properly position the C domain relative to the preceding PCP‐E unit and have corroborated this model by a mutational analysis and by conformationally dependent crosslinking. The COM‐mediated domain positioning facilitates a shortened and entropically optimized trajectory of the PCP between the two domains. This arrangement allows the PCP to efficiently visit the donor site of the C domain and probably is important for a balanced population of all necessary conformations along a catalytic cycle, according to the PCP transitions to all its domain partners (Figures 5 and S10B). PCP trajectories are likely also optimized when shuttling between other catalytic domains. For example, the trajectories between C (acceptor position) and A domains^[^ ^13^ ^]^ as well as between formylation (F) and A domains^[^ ^12^ ^]^ are structurally well investigated. The distances the PCP must travel in these two latter cases are much larger than the distance between the E and C domains that is investigated here. Their associated conformational changes also rely on differently stretched conformations of the involved A domain with its two subdomains, which are also ATP‐driven.^[^ ^62^ ^]^ Notably, the *cis*‐COM and *trans*‐COM^[^ ^18^ ^]^ domain mediated positioning of E and C domains provide the first structurally investigated multi‐domain ensembles to deal with PCP shuttling that does not involve the mobile A domain.

Extrapolating from the model of the COM domain's scaffolding role, we further propose that several other structural factors in NRPS assembly lines will have important effects on the relative population of different conformations, for example, domain size and shape as well as their positioning mediated through contact interfaces and linkers^[^ ^64^ ^]^ of different length and flexibility. The impact of these parameters on the domain interplay has hardly been studied so far.

The E‐COM‐C crystal structure can be superimposed well with the recently reported *trans*‐COM cryo‐EM structure^[^ ^18^ ^]^ (r.s.m.d. = 3.5 Å over 632 C‐α of the entire structure and 1.3 Å over 42 C‐α of the COM domain). Both structures show the same relative orientations of the neighboring E and C domains (Figure S12), supporting a common structural role,^[^ ^36^ ^]^ with only slight differences in the binding modes of the central helix (Figure S13). These similarities are consistent with our new model of the COM domain generally acting as rigid domain connector important for scaffolding. Notably, the *trans*‐COM structure was obtained after covalently fixing the dimodular TycA/TycB1 ensemble through bioorthogonal crosslinking of their Ppant moieties in the C domain's active site,^[^ ^18^ ^]^ thereby prohibiting a conclusion whether the observed domain arrangement was caused by the crosslink or by the COM domain. The present E‐COM‐C structure thus further corroborates the cryo‐EM *trans*‐COM structure. On the other hand, NMR studies and MD simulations of the *trans*‐TycA/TycB1 system suggested a helix‐down conformation and a dynamic folding of the split COM domain upon module association.^[^ ^37^ ^]^ While both our E‐COM‐C structure and the *trans*‐COM structure^[^ ^18^ ^]^ confirm the helix‐up model,^[^ ^36^ ^]^ they neither confirm nor contradict a possibly dynamic association process.

Finally, our *cis*‐COM structure, together with the recently reported *trans*‐COM structure,^[^ ^18^ ^]^ provide the long‐awaited structural blueprints to identify specificity‐determining molecular interactions in split *trans*‐COM domains that could be exploited in improved COM‐swap engineering experiments. However, the extended interaction interface, which includes substantial contact areas with the bodies of the E and C domains, suggests a limited matchmaker potential of such COM‐swaps compared to other docking domain architectures. Intriguingly, the high sensitivity of the NRPS templates investigated in this study toward structural perturbations in the *cis*‐ or *trans*‐COM domains suggests that collateral sequence changes resulting from COM‐swap experiments might cause impairment in productivity. Further research may help rationalize the success of COM‐based combinatorial engineering.

## Supporting Information

The authors have cited additional references within the Supporting Information.^[^ ^65^, ^66^, ^67^, ^68^, ^69^, ^70^, ^71^, ^72^, ^73^ ^]^

## Conflict of Interests

The authors declare no conflict of interest.

## Supporting information



Supporting Information

## Data Availability

The data that support the findings of this study are available from the corresponding author upon reasonable request.
