# Supplementary material for: Structural Basis for a Scaffolding Role of the COM Domain in Nonribosomal Peptide Synthetases
Source: Angew Chem Int Ed Engl. 2025 Jul 15;64(36):e202506621. doi: 10.1002/anie.202506621 (PMC12402864; doi:10.1002/anie.202506621)
Supplement: Supplementary file 1 — Supporting Information [file ANIE-64-e202506621-s001.pdf]

Content

Experimental section 2

DNA cloning of expression plasmids 2

Protein production 2

Cell lysis and protein purification 3

Protein crystallization 3

4`-phosphopantetheinylation 4

Asp-Asn dipeptide formation assay 4

D-Phe-L-Pro-diketopiperazine assay (DKP assay) 5

NRPS aminoacylation assays 5

Photo-activated crosslink reaction with TycB1(S5Bpa) 5

Densitometric analysis of crosslink bands 5

Tryptic digestion and MSMS analysis of crosslink bands 5

Mass analysis of intact proteins 6

Supporting Figures 7

Supporting Tables 26

Amino acid sequence 29

Supporting References 34

Experimental section

DNA cloning of expression plasmids

The gene fragment encoding BacC4-C5[E-COM-C] was PCR-amplified from chromosomal DNA of *Bacillus licheniformis* ATCC 10716 (DSM 603; DSMZ-German Collection of Microorganisms and Cell Cultures GmbH) using oligonucleotides 5´-GTTACCATGGACTCAGATGAAGAAATGAAC-3´ and 5´-CATGGATCCTATTTTGACAG TCTTATTGATG-3´. The fragment was digested using *Nco* I and *Bam* HI and ligated into pET28a to give pAF24.

The gene fragment encoding BacC4-C5[A-PCP-E-COM-C-A-PCP-TE] was PCR-amplified from the same chromosomal DNA using oligonucleotides 5´-GCTGACCATGGGTGATAAATCAGCATCATATG-3´ and 5´-GCTCTGGATCCGGAAAGGACTGTGATATCG-3´ and ligated into pET28a using restriction sites *Nco* I and *Bam* HI to give pJD01. Derivatives of pJD01 coding for mutations, deletions and insertions in the COM domain were introduced using site-directed mutagenesis. All resulting amino acid sequences are given below.

pJR89 encoding SBP-TycA[A-PCP-E-COM^{D}] was created from pJR88 (Ref[1]) by introducing a stop-codon following the gene encoding TycA to remove the following His-tag from the open reading frame and create the native C-terminal end in the encoded SBP-TycA. Derivatives of pJR89 to encode for mutations, deletions and insertion in the COM domain were introduced using site-directed mutagenesis. All resulting amino acid sequences are given below.

pBH96 was created by introducing an amber stop-codon into pJR95 (Ref[2]) using site-directed mutagenesis at the position coding for S5 in TycB1[COM^{A}-C-A-PCP].

See Table S2 for all plasmids encoding the recombinant proteins.

Protein production

Proteins were produced in *E. coli* LOBSTR BL21(DE3) cells carrying the encoding plasmid and the plasmid pLacIRARE2 that decodes rare codons. LB medium containing the antibiotics ampicillin at 100 µg/mL or kanamycin at 50 µg/mL and chloramphenicol at 34 µg/mL was inoculated 1:100 from an overnight culture. The culture was incubated at 37°C until the optical density of OD_{600} ~0.8 was reached. Gene expression was induced by adding IPTG to a final concentration of 400 µM and conducted at 18°C for 16 hours and 180 rpm.

Cells expressing the genes encoding BacC4-C5-His_6 wild type or mutant proteins thereof were harvested by centrifugation and resuspended in 10-15 mL ice-cold HEPES A buffer (50 mM HEPES, 100 mM NaCl; pH 8.0) for the purification via Ni^{2+}-NTA affinity chromatography.

Cells expressing the genes encoding SBP-TycA wild type or mutant proteins thereof were harvested by centrifugation and resuspended in 15 mL ice-cold Strep-Tactin buffer (100 mM Tris/HCl, 150 mM NaCl; 1 mM EDTA, pH 8.0) for the purification via Strep-Tactin affinity chromatography.

Cells expressing the gene encoding TycB1-His_6 were harvested by centrifugation and resuspended in 15 mL ice-cold HEPES A buffer (50 mM HEPES, 150 mM NaCl, pH 8.0) for the purification via Ni^{2+}-NTA affinity chromatography.

TycB1(S5Bpa) containing p-benzoylphenylalanine (Bpa) was produced in LOBSTR BL21(DE3) cells using nonsense suppression. The cells carried the plasmid encoding TycB1(S5*tag*)-His_6 as well as the plasmid pEVOL-Bpa encoding the tRNA-synthetase /$tRNA_{CUA}$ pair.[3] LB medium containing the antibiotics ampicillin at 100 µg/mL and chloramphenicol at 34 µg/mL was inoculated 1:100 from an overnight culture. The culture was incubated at 37°C until the optical density of OD_{600} ~0.7 was reached. Before gene expression was induced by the addition of IPTG at a final concentration of 400 µM as well as 0.2 % (w/v) L-arabinose, Bpa

was added at a final concentration of 1 mM (dissolved in 1 M NaOH). Gene expression was conducted at 28°C for 4 hours and 180 rpm.

BacC4-C5[E-COM-C]-His_6 was produced in *E. coli* LOBSTR BL21(DE3) cells carrying the encoding plasmid. LB medium containing kanamycin at 50 µg/mL was inoculated 1:100 from an overnight culture and incubated at 37°C and 180 rpm until the optical density of OD_{600} ~0.7 was reached. Gene expression was induced by the addition of IPTG at a final concentration of 400 µM and conducted at 28°C for 4 hours and 180 rpm.

The gene encoding BacC4-C5[E-COM-C]-His_6 was also expressed as selenomethionine derivative. The protein was produced in LOBSTR BL21(DE3) cells carrying the encoding plasmid. M9 medium (Na_2HPO_4 6,8 g/L, KH_2PO_4 3 g/L, NaCl 0.59 g/L, NH_4Cl 1 g/L, glucose 2 g/L, $MgSO_4$ 0.241 g/L, $CaCl_2$ 0.011 g/L, thiamine 0.5 mg/L) containing all necessary amino acids, selenomethionine and the corresponding antibiotic kanamycin at 50 µg/mL was inoculated 2:100 with harvested cells from an overnight culture. The culture was incubated at 37°C until the optical density of OD_{600} ~0.8 was reached. Gene expression was induced by the addition of IPTG to a final concentration of 400 µM and conducted at 16°C for 16 hours and 180 rpm.

Cell lysis and protein purification

To purifiy proteins via their C-terminal His_6-tags, the cells were disrupted by a homogenizer (EmulsiFlex-C5, Avestin). The suspension was centrifugated 30 min at 25,000 x g and 4°C to separate soluble proteins from insoluble material and cell debris. The soluble protein fraction was adjusted to 20 mM imidazole. A Ni^{2+}-NTA gravity flow column was equilibrated with HEPES A containing 20 mM imidazole (HEPES A-20) and the soluble fraction was loaded to the column twice. The column was then washed with 15 column volumes (cv) of HEPES A-20 and 10 cv of HEPES A-35 containing 35 mM imidazole. Proteins were eluted with HEPES B containing 250 mM imidazole. In the case of selenomethionine, all buffers were degassed and DTT was added at a final concentration of 1 mM to prevent oxidation.

The pooled fractions (selected by concentration and purify check using SDS-PAGE and Coomassie staining) were dialyzed first against assay buffer (50 mM HEPES, 100 mM NaCl, 10 mM $MgCl_2$, 1 mM EDTA, pH 7.0) + 2 mM DTT, followed by buffer without DTT, and finally against buffer with 10 % glycerol. Proteins were concentrated when concentrations were below 25 µM using spin columns with a molecular cut-off of 30 kDa (TycA and TycB1 constructs) or 100 kDa (BacC4-C5 constructs) (Thermo Scientific™ Pierce™ Protein Concentrators PES 100 K MWCO).

After affinity chromatography of BacC4-C5[E-COM-C]-His_6 and its selenomethionine derivative, the pooled fractions were dialyzed against crystallization buffer X (50 mM HEPES, pH 7.5, 300 mM NaCl,). In the case of the selenomethionine derivate, buffer X was degassed and 2 mM TCEP was added. Size exclusion chromatography (Superdex 200 10/300 GL, Cytiva) with buffer X was carried out to remove protein aggregates. The selected pooled fractions were concentrated to a final concentration of 15 mg/mL using spin columns with a molecular cut-off of 30 kDa (Thermo Scientific™ Pierce™ Protein Concentrators PES 30 K MWCO).

All steps were performed at 4°C. Protein concentrations were determined using calculated extinction coefficients at 280 nm.

Protein crystallization

Elongated, rod-shaped crystals were grown at 12°C in a hanging drop vapor diffusion approach from an optimized initial hit derived from a MORPHEUS sparse matrix screen.[4] The final crystallization condition contained 0.09 M halogens mix (Molecular Dimensions, MD2-100(250)-71; 0.3 M, sodium fluoride, 0.3 M sodium bromide, 0.3 M sodium iodide), 0.1 M buffer mix 3 pH 8.7 (Tris-base, BICINE), 36% precipitant mix 4 (Molecular Dimensions MD2-100-84; 25% MPD, 25% PEG 1000, 25% PEG 3350). The crystals were mounted on nylon loops and cooled in liquid nitrogen.

Diffraction data were collected at beam line P13 (PETRA III) at 0.979510 Å and 100 K allowing collection of anomalous scattering from the selenium atoms. The data were processed to 3.29 Å using XDSAPP2 (Ref[5]) with Friedel's pairs as individual reflections. Single-wavelength anomalous diffraction phasing was used to determine the selenium substructure and generate an initial structural model with AutoSol[6-7] that was improved with AutoBuild.[8] The model was further refined in Coot[9] and phenix_refine.[10] The final structure was deposited at Protein Data Bank under PDB accession number 9RDE.

4`-phosphopantetheinylation

Apo-BacC4-C5-His_6 wild type and mutant proteins were converted into their holo-forms by a 40 min incubation at 25°C with 0.05 eq. of 4`-phosphopantetheinyl transferase Sfp (*B. subtilis*) and 50 eq. coenzyme A in the presence of 10 mM $MgCl_2$ and 1 mM TCEP. Apo-SBP-TycA and apo-TycB1-His_6 wild type and mutant proteins were converted into their holo-forms at a concentration of 10 µM by a 3 hrs incubation at room temperature with 0.02 eq. of Sfp and 50 eq. coenzyme A in the presence of 10 mM $MgCl_2$ and 2 mM TCEP.

Asp-Asn dipeptide formation assay

In the following section, activity means ATP-dependent Asp-Asn dipeptide formation. For each holo-dimodule of interest as well as for each wild-type control, two aliquots with one reaction volume containing 7 µM of the respective protein were prepared with 2 mM of L-Asp, 2 mM of L-Asn and 20 mM $MgCl_2$. ATP was added at a final concentration of 5 mM to one aliquot, whereas the other was complemented with buffer. After a 50 min incubation at 37°C, an aliquot was withdrawn from each reaction mixture for SDS PAGE analysis. The reaction was quenched and peptides and amino acids were dansylated by adding one reaction volume of a 75 mM dansyl chloride solution in dry *N*, *N*-dimethylformamide and 1 reaction volume of a 1 M Na_2CO_3 solution to the remaining reaction mixture. After a 45 min incubation at 60°C in the dark, the reaction was stopped by acidification with formic acid to a final concentration of 17% (v/v). Precipitates were removed by centrifugation (5 min, 18400 x *g*). The supernatant was used for MS analysis.

To quantify the dansylated products, 3 µL of the supernatant were subjected to LC-MS analysis on a maXis II UHR qTof spectrometer hyphenated to an Ultimate 3000 RS HPLC, which was equipped with a C18 reversed phase column (ZORBAX SB-C18 RR HT, 80 Å, 1.8 µm, 50 mm x 3 mm, Agilent Technologies, Waldbronn, Germany). The analyte solution was injected at 0.6 mL min^{-1} and 1% eluent B (eluent A: 0.1% formic acid in water, eluent B: 0.1% formic acid in acetonitrile) and exposed to a linear gradient reaching 40% B at 6 min and 95% B at 7 min. MS settings: range m/z 120 to m/z 1300, spectra rate 1 Hz, capillary voltage 4500 V, endplate offset -500 V, nebulizer pressure 3.0 bar, dry gas flowrate 8 L min^{-1}, dry gas temperature 250°C. The system was controlled using Bruker Compass HyStar 6.0 and otofControl 5.2 (Bruker Daltonik GmbH, Bremen, Germany). After each run, a processing method was automatically carried out, which encompassed a lock mass calibration and generated an extracted ion chromatogram (EIC) for $[M+H]^+$ of $C_{20}H_{24}N_4O_8S$ (positive polarity, width ± 0.02, monoisotopic peak only). All signals within 3.9 min to 4.2 min were integrated and results exported to csv files. Data from csv files were manually imported into Excel and used for further analysis. Chemically synthesized standards of L-Asp-L-Asn-OH and D-Asp-L-Asn-OH were purchased from Davids Biotechnologie GmbH (Regensburg, Germany) and used for calibration of product yields by spiking defined amounts into the reaction mixture lacking ATP before the dansylation step.

Wild-type activity was repetitively measured, as well as the background in the absence of ATP. After subtraction of the background values, linear regression of the wild-type values provided the basis for normalization of the mutant values. In a final step, mutant activities were corrected for the actual protein concentrations of each BacC4-C5 construct. To this end, the withdrawn aliquots from each reaction mixture were run on SDS PAGE gels (8%). Gels were digitized and the intensities of the protein bands corresponding to the BacC4-C5 constructs were densitometrically determined using ImageJ 1.54i (Wayne Rasband and contributors, NIH, USA) and used to calculate concentration-based correction factors relative to the wildtype

construct (**1**). All mutant activities were measured in triplicates (three samples, three gel lanes). Columns in the respective graphs show the averaged values, error bars indicate the standard error.

D-Phe-L-Pro-diketopiperazine assay (DKP assay)

DKP assays were performed using 5 µM of holo-proteins SBP-TycA and TycB1-His$_6$ in a total volume of 100 µL. For the reaction, L-Phe (1 mM), L-Pro (1 mM), ATP (5 mM) and $MgCl_2$ (20 mM) were added, and the reaction mixture was performed for 30 min at 37°C. The reaction was stopped using 400 µL of an nBuOH/$CHCl_3$ mixture (4:1) and 200 µL of ddH$_2$O. Separation of the product was performed via 20 seconds of vortexing, followed by 2 minutes of centrifuging (15870 x *g*, rt) for phase separation. The organic layer was collected. 400 µL of the nBuOH/$CHCl_3$ mixture were added two additional times together with 300 µL of ddH$_2$O and the procedure repeated. The combined organic solvents were removed in vacuum and the residue was resuspended in 30 to 600 µL of HPLC buffer A (ddH$_2$O with 0.1% trifluoroacetic acid and 5% acetonitrile (v/v)). For HPLC analysis, 20 µL were loaded on a C18 reversed phase column (ZORBAX SB-C18 RR HT, 80 Å, 1.8 µm, 50 mm x 3 mm, Agilent Technologies, Waldbronn, Germany) at a flow rate of 0.4 mL min^{-1} and subjected to a gradient starting from 5% eluent B (eluent A: 0.1% trifluoroacetic acid in water; eluent B: 0.1% trifluoroacetic acid in acetonitrile) to 80% B at 20 min, 80% B at 22 min, 5% B at 25 min and 5% B at 30 min. The absorbance of the eluate was monitored at 210 nm.

NRPS aminoacylation assays

In a total volume of 30 µL, 7 µM holo-BacC4-C5-His$_6$ wild type and mutant proteins were incubated with 2 mM of the respective amino acid and 1 mM ATP for 10 min at 25°C to trigger aminoacyl thioester formation. The reaction was quenched by adding formic acid. The supernatant of the subsequent centrifugation (5 min, 18400 x *g*) was used for MS analysis.

10 µM holo-SBP-TycA and holo-TycB1-His$_6$ proteins were incubated with 1 mM of the respective amino acid and 5 mM ATP in the presence of 20 mM $MgCl_2$ at 37°C. Aliquots were removed after 30 s, 60 s and 300 s. Each aliquot was quenched by adding formic acid. The supernatant of the subsequent centrifugation (5 min, 18400 x *g*) was used for MS analysis.

Photo-activated crosslink reaction with TycB1(S5Bpa)

Photo-activated crosslink reactions were performed using 5 µM of holo-SBP-TycA (5 µM) and holo-TycB1(S5Bpa)-His$_6$ in a total volume of 20 µL. The reaction mixture was preincubated for 30 min at 37°C and subsequently the crosslink reaction was performed for 60 min at 25°C using UV-light of 365 nm (Herolab UV-16L, 2x8 W). Subsequently, 9 µL of the reaction mixture were mixed with 3 µL of 4x SDS-PAGE buffer, of which 10 µL were analyzed via SDS-PAGE (6% acrylamide gel) and Coomassie staining.

Densitometric analysis of crosslink bands

Analysis of crosslink bands was performed using Coomassie-stained SDS gels and *GelAnalyzer* software (Istvan Lazar Jr.), basically as previously described.[11] L- and T-form crosslink band intensities were calculated using a rolling ball method and baseline correction in combination with manual integration.

Tryptic digestion and MSMS analysis of crosslink bands

For tryptic protein in-gel digestions, proteins or mixtures were mixed with 4x SDS loading dye (250 mM Tris/HCl, pH 6.8, 8% (w/v) SDS, 40% (v/v) glycerine, 20% (v/v) β-mercaptoethanol, 0.2% (w/v) bromophenol blue) and heated to 95°C for 5 min. Following separation by SDS-polyacrylamide gel electrophoresis and Coomassie brilliant blue staining, the gel was then destained using 50% (v/v) EtOH and 10% (v/v) acetic acid in H2O. The gel pieces containing the crosslink bands were excised and washed in H_2O, followed by a further destaining step using 50% (v/v) MeOH in 50 mM ammonium carbonate. Gel pieces were washed with acetonitrile and dried in a vacuum concentrator. Once dried, the gel pieces were reduced by a 25 min incubation with 25 mM DTT in 50 mM ammonium carbonate and subsequently alkylated by addition of

iodoacetamide to a final concentration of 55 mM. After another wash and drying step, the gel pieces were rehydrated in 20 µL trypsin solution, consisting of 400 ng pre-warmed trypsin (Promega) in 50 mM ammonium carbonate supplemented with 0.01% ProteaseMax (Promega). After 10 min 30 µL buffer were added and then digested for 1 h at 50°C. After the digestion process the supernatant (50 µL) was collected and acidified with formic acid at a final concentration of 0.1% and analyzed by LC-MS without further dilution.

Tandem MS analysis of tryptic peptides (LC-MS^2) was performed using an UltiMate™ 3000 RS LC nano system (Thermo Fisher Scientific GmbH) connected to a maXis II UHR-qTOF mass spectrometer with a nano-ESI source (CaptiveSpray with nanoBooster, Bruker Daltonik GmbH). 3.5 µL of each sample were loaded on a C18 trapping column (Acclaim PepMap™ 100, 5 µm, 100 Å, ID 100 µm x L 20 mm, Thermo Fisher Scientific GmbH) at a flow rate of 20 µL/min in 2% eluent B (eluent A: 0.1% formic acid in water; eluent B: 0.1% formic acid in acetonitrile). After 10 min of washing at 2% B, a 180-minute gradient (2 to 40% B, flow rate 500 nL/min) was applied for the separation on a C18 nano column (PepSep TWENTY-FIVE C18, ID 150 µm x L 250 mm, 1,5 µm, Bruker Daltonik GmbH). MS settings: capillary voltage 1600 V, mass range: m/z 150-2200. MS survey scans were performed with a cycle time of 2.5 s. After each survey scan, the 10 to 20 most abundant precursor ions with z > 1 were selected for fragmentation using collision-induced dissociation. MS/MS summation time was adjusted depending on the precursor intensity, the precursor isolation window and the collision energy were depending on the precursor m/z and charge. DataAnalysis (v5.3) (Bruker Daltonik GmbH) was used for chromatogram processing and fragment spectra isolation. The resulting mgf files were analyzed using StavroX (version 3.6.6).[12]

Mass analysis of intact proteins

Mass analysis of intact proteins was performed using an UltiMate™ 3000 RS system (Thermo Fisher Scientific GmbH) connected to a maXis II UHR-qTOF mass spectrometer (Bruker Daltonik GmbH) with a standard ESI source (Apollo, Bruker Daltonik GmbH). When necessary, proteins were reduced with 2 mM TCEP at 4°C for 10 min to avoid inhomogeneity issues. Then, samples were acidified using a 10% formic acid solution to reach a pH 2-3 and centrifuged (20,000 x g, 3 min). According to the protein concentration, an appropriate volume of the supernatant was loaded on a C4 column (Advance Bio RP-mAb C4, 2.1 mm x 50 mm, 3.5 µm, Agilent Technologies) at a flow rate of 0.6 mL/min in 5% eluent B (eluent A: 0.1% formic acid in water; eluent B: 0.1% formic acid in acetonitrile). After a desalting period of 7 minutes at 5% B, a steep gradient was applied (5-60% B in 2 min). MS settings: capillary voltage 4500 V, endplate offset 500 V, nebulizer 5.0 bar, dry gas 9.0 L/min, dry T = 200°C, mass range m/z 300-3000. Data were analyzed with DataAnalysis (version 5.2) (Bruker Daltonik GmbH) and deconvolution was performed using the MaxEnt algorithm implemented in the software.

Supporting Figures

A

cis-COM domains

```
                  E-domain                                                                                     C-domain
                  -------                                                                 ----------------------------------------------------
BacC4-5           KAVIRHCAE------REETEKTPSDYGDKGISLDQLEEIKLKYKG-------------------MEIEKIYPLANMQRGMLFHALEDKESQ-AYFEQMAINMKGLIDERLFAETFNDIMERHE
BacA4-5           EKIVDHCVD------KEGSDMTPSDYGDVSLGLEELELIKDKYS--------------------AFQIEKIYPLANMQKGMLFHNAMDQTSG-AYFQQIVIKLKGRVHPDILEESFHEIVKRHE
BacC2-3           EQIIKHCTQ------QTESERTPSDYGDTNISLAELEEIKGKY---------------------RSAIEKIYPLANMQKGMLFHAIEDHTSD-AYFQQTVMDIEGYVDPAILEASFNDIMKRHE
LgrB2-3           RAIIAHCRT------EQAGGYTPSDFPLAELDQNSLDKFIGHNR----------------------LIENVYTLTPLQEGMLFHSLYEQAGG-DYVVQLALKLEH-VNVEAFSAAWQKVVERHA
LgrC2-3           REIMEHCRS------EEAGGYTPSDFPLARLDQRAIDNYVGRDR----------------------SIENVYPLTPLQEGMLFHSLYEHAGG-DYVVQFSMTMHH-VEVDVFQQAWQKVVDRHS
LgrC4-5           REIIAHGQS------EAAGGYTPSDFPLARMDQRALDKYLGQNR----------------------SIENVYPLSPLQGGMLFHSLYEQEGG-DYVVQLAMTVEG-LDVEAFEQAWQKVVDRHS
LgrD2-3           REIVAHCTL------PEAGGYSPSDFPLAVLEQKQIDKHIGFDR----------------------QIEDVYTLSPLQQGMLFHSLYNQDSG-DYVVQFAVTFQN-LDVSVLEKAWQNVLDRHS
FegR2-3           TGLVTHVSR------PGSGGRTPSDFPLVALDQARIEQLEAEV----------------------PGLAEVLPVSPLQEGLLFHALFDEQGTDVYVEQMVLDLEGPLDSVALRASWQALLDRHA
FegR4-5           SGLAAHVAG------PGSGGRTPSDFPLVALDQARIEQLEADV----------------------PGLAEVLPVSPLQEGMLFHALYDEQGTDVYVEQMSTGLEGELDAVALRASWQALLDRHA
FegQ2-3           TGLATHVSR------PGSGGRTPSDFPLVELDQPQIEELETQH----------------------PGLADVLPVSPLQEGLLFHALFDESDTDVYVEQMDLGLDGPLDARALRGAWQALTARHA
FegQ1-2           TGLTVHTSR------PGGGGRTPSDLPLLALTQPQVEELEAGE----------------------PEFVDILPVTPLQEGLLFHAQFDENTRDVYVEQMLLDLRGPLDVRALRASWAAMLDRHP
DptA2-3           EGLVAHARR------PDAGGLTPSDLPLVALDHAELEALQADVTG---------------------GVHDILPVSPLQEGLLFHSSFAADGVDVYVGQLTFDLTGPVDADHLHAVVESLVTRHD
Tcp11_4-5         TGLATHAGD------PRAGGHTPADFDLVEVTQRDVTALEAAAPGLT----------------------DIWPLSPLQRGMLFERALDEDGVDVYQSQRILDLDGPLDADRLRAAWRTLVARHE
Tcp11_5-6         AGLAVHAGD------VRAGGHTPSDFPLTALSGQEVAQIEAAVPDLL----------------------DIWPLSPLQEGLLFHAVD-ERGPDVYASMRTLAVDGPLDVARFRASWQAVLDRHA
```

trans-COM domains

```
                                              COM^D                           COM^A
                                  --------------------------------     -------------------
TycA-B            LAIIAHCTE------KKEVERTPSDFSVKGLQMEEMDDIFELLANTLR           MSVFSKEQVQDMYALTPMQEGMLFHALLDQEHN-SHLVQMSISLQGDLDVGLFTDSLHVLVERYD
TycB-C            LGIIEHCMA------KEEGEYTPSDLGDEELSMEELENILEWI                  MKKQENIAKIYPLTPLQEGMLFHAVTDTGSS-AYCLQMSATIEGDFHLPLFEKSLNKLVENYE
GrsA-B            LAIIEHCVQ------KEDTELTPSDFSFKELELEEMDDIFDLLADSLT           MSTFKKEHVQDMYRLSPMQEGMLFHALLDKDKN-AHLVQMSIAIEGIVDVELLSESLNILIDRYD
SrfAA-AB          LRIIEHCLS------QDGTELTPSDLGDDDLTLDELDKL                   MEIFMSKKSIQKVYALTPMQEGMLYHAMLDPHSS-SYFTQLELGIHGAFDLEIFEKSVNELIRSYD
SrfAB-AC          LMLIRHCTE------KEDKEFTPSDFSAEDLEMDEMGDIFDMLEENLK           MSQFSKDQVQDMYYLSPMQEGMLFHAILNPGQS-FYLEQITMKVKGSLNIKCLEESMNVIMDRYD
LicA-B            LQLIDHCLE------RDGGELTPSDLGDDELTLEELDKL                   MEIFMGKQKIQKVYPLTPMQEGMLYHAMLDPESS-SYFTQLELFIDGEFDLGIFEKSVNQLVRSYD
LicB-C            TALIDHCTA------KEEREFTPSDFSAGDLEMEEMGDLFDVLEENLK     MKGDTILSQFKKEHVQDIYYLSPMQEGMLFHTLLHPGQS-FYIEQISMQVKGSFQKDILEKSMNVIIGRYD
BacB-C            QMVISHCTG------KHETEKTSSDYGYDKLSLEDLEELLNEYESVDS               MKTKVEKIYPLSNMQKGMLFHAMKDEASH-AYFEQFIIELKGDVDERMFEESLNEVMKRHE
LgrA-B            NALLAHCLQ------KTETELTPSDFVDKNLSRSELDDIMDLISDL                MSRKKVDNIYPLTPMQEGMLFHSLLDEGSE-SYFEQMRFTIKGLIDPAILEQSLNALIERHD
LgrB-C            QSLIQAEKQSYRAEDFEDADLSQSALNKVLARLKNRKGNELHGGSH               MNSMGDLTDLYTLTPLQEGMLFHSLYS-EGS-AYMIQTTAILTGELDIVPFEKAWKKVIQRHS
LgrC-D            RQLLAHAKT------DKAAAPAQSAASNLSEFGWDDEEIADLLDLIQDK               MNNIETYYPVTPLQQGLIFHSLLEPESG-AYIVQMGLKLQGPLNIPLFEQAWQCLVDRHA
FengA-B           EDIIAHCSG------KKEREKTLTDYSHTELTAQALSSIEDLVKGL               MTQATEIQDIYPLSYMQEGMLFHSLLDSGSN-AYVEQISFTVSGDLCIDTFQKSLDMLVSRYD
FengB-C           KQIISHCTG------KEEREWSAADFSDEELTLEDLSDIMGAVNKL               MGQQPEIQDIYPLSFMQEGMLFHSLLDHDSR-AYFEQAVFTINGQLDRERFQKSIDAVFERFD
FengC-D           RDIMAHCTG------KEEAEKTVSDFSSKTLTSDDLDSIASFVEEL               MTKKNAIQDIYPLSYMQEGMLFHSLLQKESQ-AYAEQASFSITGKVDTRVFEESVHALFDRHD
FengD-E           LKLSEHCLS------KTDTEKTVSDFDDRELTEEALQDIADLLSFH               MDKTKNIQNIYPLSRMQEGMLFHSFLQKEGA-AYIEQSVFTIKGRLRPELFEQSVQSVISRHD
PpsA—B            IETIEHCSQ------KKAREKTLSDFSNKELTLSALSSIEDLVKDL               MAQSAQIQDIYPLSHMQEGMLFHSLMDFSSK-AYIEQTSFTITGNLCVDSFQKSLNLLVSRYD
PpsB-C            ENIIEHCTG------KENQEWSASDFTDEDLTLDELSEIMGAVNKL               MPQQPEIQDIYPLSFMQEGMLFHSLYDEQSR-AYFEQASFTIHGQLDLERFQKSMDAVFDRYD
PpsC—D            LDIMKHCAG------QQKAEKTLSDFSSQSLTAEDLDSISSLVEEL               MTKANSIQDIYPLSYMQEGMLFHSLLQKDSQ-AYVEQASFTIEGKVNPQFFQNSINALVERHD
PpsD—E            LQLIEHCQN------KSETEKTISDFDDQELTEDALQEIADMLSFH        MKKGADTMNTIKKIKNIYPLSHMQEGMLFHSFLRKEEG-AYVEQSLFTIKGSLSYDWFQRSIQAIIDRHD
FegR-S            TGLVTHTSD------AGSGGYTPSDFPLVDISQDELDEFEAMAKQIDEGA            MSDSALVGVWPLSPLQEGMLFHAVYDEHGTDVYVEQISTGLEGRLDTVVLRASWQAVLDRHE
Tcp9-10           SGLAGQADD------PSAGGHTASDFDLLDLDQDEIENFEAIAAEFGGGQES    MTTPTTSPGSALAEVWPLSPMQEGMLYHASFDDAAPDIYVIQQSQIIDGPLDTERFRRSWEVLLHRHA
                                       : :           .  .                                  :: :* *:::.          :       .   .    :      :   .
```

>>> Figure S1 continued on next page

```
                                                                        C-domain                                      >>> (seq. truncated)
                  ------------------------------------------------------------------------------------------------------------------------
                            COM^A                                          COM^A
                     ------------------                                    ----
BacC4-5           ILR--ASIEYEITDEPRNVIIKDRKINLDYHDLRKQSPAEREQVIQAYRKADREKGFRLNSEPLIRAALMRTEDDSYTFIWTNHHILLDGWSRGIIMGELFHMYHMKEARQKHRLEEARP
BacA4-5           ILR--ASFEYEITAEPRQIIARDRKTPFTSIDLTGENRTRQHRFIETYLKEDQEKGFDLSSEALMRVCLIKMSDESYRLIWSHHHILLDGWCLGIVLSELFSLYGKIMKGESRRLKEPKP
BacC2-3           ILR--ASYEYEIVEEPRQIIIENRSIDFTYFNIAKSSAQQQEMFIERLLNEDRKKGFDLSKDVLMRAYLLKTAERSYRLVWSHHHILLDGWCLGIIMRELFVIYENRMNGKASPLKETKP
LgrB2-3           ILRTSFLWSG--LEKPHQVVHAKVKTFVERLDWRHLTAAEQEAGLQTYLEQDRKRGFDLARPPLMRWTLIRLDASTFQFVWSFHHMLLDGWSTPIVFQDWQAFYAAASHGKEASLPAIPP
LgrC2-3           ILRTSFIWDG--VSTPHQIVRKHVQVVVDEQDWRHVPADQQKAEWDAFLEEDRKRSFAITEPPLMRWTLLRISDTAYRFIWSFHHVLLDGWSVPLVMKDWFAAYMALADGKDIQFGAVHP
LgrC4-5           ILRTSFIWEG--LTEPHQVVRKQVKACVEKIDLRHLTPDQQKAELSEYLAADRRRSFEIAVAPLMRWTLFRLSESAYRFTWSFHHVLLDGWSIPIVLKDWFSAYLSLAEGKEVAHSFVQP
LgrD2-3           ILRTHFVWEG--LSEPHQVVRKDVKVTLTKEDWRHLQADVQDEMLAAFLEEDRRRSFDIAQAPLSRWVVFQTKDEEYRFVWSFHHVLLDGWSVPIVLNELLAHYAAISEGREGKLVPSQP
FegR2-3           SLRAGFRQLDG-LDEPVQVIARSVTLPWREVDLSALDGEAASAAAERLAGEDQARRFDVAVPPLLKVMLVKVGPDRYRMAVTLHHILLDGWSLPILMQELWSAY--EAGGSVAALPRVTP
FegR4-5           SLRAGFRQLDG-VDQPVQVIAQGVTLPWREVDLSALAEDEALAEAERLSAEERDLGFRMTVPPLLKVLLVKVGADRYRMSVTLHHILLDGWSLPIMMRELWDAY--AAGGSAAGLPAVTP
FegQ2-3           SLRAGFRQLPG-VEQPVQVIVAREVTLPWREVDLSALPENEALAEAARLGVEERARRYDLSAPPLLRILLVKVGETRHRMMVTLHHILLDGWSLPILTRDLWAAY--AAGGSTTGLPPVIP
FegQ1-2           ALRAGFRRIPG-LKQPVQTITARATLPWREEDLSALGRDAAHAEARRLEDEERARRFDVQQPPLVRVLLTALGDGHHRMVVTLHHLVLDGWSLPVLVRELWSAY--DAGGDAGALAPVTP
DptA2-3           VLRTGYRQAQ--SGEWIAVVARQVHTPWQYIHTLDT-------DADTLTNDERWRPFDMTQGPLARFTLARINDTHFRFIVTYHHVILDGWSVAVLIRELFTTYRDTALGRRPEVPYSPP
Tcp11_4-5         SLRTSFHQLE--SGETVQVVVDQADIGWRVADVSHRAEADAAAEVGRLLAEDQAQRFDVTRAPLLRLLLVRLGADRHRLVVTSHHIVLDGWSTPIILGEMSAAY--AGAPSTATAPSP--
Tcp11_5-6         ALRASFHQLE--SGAAVQAIAREVTLPWQETDLSDLPEDVALAEFDRLAAQLRDERFDLTRAPLLRLHLVRLGERRHRLAFASHHISCDGWSLPIVSTEVMAAY--EGR----GLPAPTS
TycA-B            VFRTLFLYEK--LKQPLQVVLKQRPIPIEFYDLSACDESEKQLRYTQYKRADQERTFHLAKDPLMRVALFQMSQHDYQVIWSFHHILMDGWCFSIIFDDLLAIYLSLQNKTALSLEPVQP
TycB-C            VLRTAFVYQN--MQRPRQVVFKERKVTVPCENIAHLPSAEQDAYIQAYTK--QHHAFDLTKDNLMKAAIFQTAENKYRLVWAFHHIIVDGWTLGVLLHKLLTYYAALRKGEPIPREATKP
GrsA-B            VFRTTFLHEK--IKQPLQVVLKERPVQLQFKDISSLDEEKREQAIEQYKYQDGETVFDLTRDPLMRVAIFQTGKVNYQMIWSFHHILMDGWCFNIIFNDLFNIYLSLKEKKPLQLEAVQP
SrfAA-AB          ILRTVFVHQQ--LQKPRQVVLAERKTKVHYEDISHADENRQKEHIERYKQDVQRQGFNLAKDILFKVAVFRLAADQLYLVWSNHHIMMDGWSMGVLMKSLFQNYEALRAGRTPANGQGKP
SrfAB-AC          VFRTVFIHEK--VKRPVQVVLKKRQFQIEEIDLTHLTGSEQTAKINEYKEQDKIRGFDLTRDIPMRAAIFKKAEESFEWVWSYHHIILDGWCFGIVVQDLFKVYNALREQKPYSLPPVKP
LicA-B            ILRTVFVHQQ--LQKPRQVVLAERQAIVEFEDLADLDEEKQNNRIDRYKQEVQAAGFNLAKDMLFKTAVFRLDRNKYLLVWSNHHIVMDGWSMGILMKRLFQNYEAFRANRTVPLDQGKP
LicB-C            IFRTVFVHEK--MKRPVQVVLKERSFQAEEIDLSGLSEAEQNERIEDYKRKDKEKGFNLSKDIPMRTAVFKKGQDRYEWVWSYHHILLDGWCFGIVVQELFEVYNALRENRLYSLGPVKP
BacB-C            ILR--ASFHHRLD-EPLHVIIKDRHMKFDYLDIRG--RHDQDGVLERYLAEDRQKGFDLAKDTLMRACLIRMSDDSYQFVWTYHHILLDGWCLGIILDELLTIYEMKRKGQNHQLEDPRP
LgrA-B            ILRTVFLLEK--VQKPRQIVLRERKTKVQVLDITHLSEGEQAAYLEDFAQKDRQASFDLAKDVLIRLTLVRTSADTHTLFWSHHHILLDGWCIPIVLNDFFQIYQQRKGGLPVELGPVYP
LgrB-C            ILRTGFIWEE--TEKPLQAVFESVPFSIRQKDWSSYGSDEQESMLAAFLQNEKAAGFDLSEAPLMRVTIIKLGEAVHRLIWSFHHLLLDGWSSPIVFQEVLDFYEAYRQGKDLRLPQARP
LgrC-D            IFRTRFVGGK--VKEYVQVVLKDLKISLVEHDLIHLSSSEQEAFLHHFAKEDRKRGFDIEQAPLMRLNVFHLNSETVHFLWTLHHVLIDGWSMPLVFGEVFAAYEMLSKGQPLSLPPVRA
FengA-B           IFRTIFIKEVPDLEGPQQVVLSRRDTAVRTEDISDYSEERQQSIIEEFKETDRHKGFDLQKGPLMRLTLFQTGENRHTCVWTHHHIMMDGWSLGIVLKDFFSMYYANRNGRPVNLGSPAP
FengB-C           IFRTTFIHKN--VAKPRQVVLKNRPSRVQFHDISHLDEKAQDKYTDRFRKEDKDKGFDLQSDPLMRVSILKKAPERYVCIWSHHHIVMDGWCFGIVMKEFLMIYQSLGDGRLPSLEPVQP
FengC-D           IFRTIFISQN--VSVPQQVVLKERNVSIIEENLTNLNKADQIKHIEEAKRRDRKKGFHLQKDMLMRVTLLQTGECEYTCIWSFHHIIMDGWCLGIVLKEFFQIYASRLRRTPLTLEPAVP
FengD-E           IFRTVFLPHVAQLNGPRQVVLREREFRLHREDLTHLDEAEQSIYLQRFKERDRLKGFDLQKDMLMRVSLFKTADEKYICVWSHHHILMDGWCLGIVLQEFMHMYRAIESGSPVTLAPAKP
PpsA—B            IFRTIFIKEVPDLTGPQQVVLSNRELTVYREDISRLADQEQQTLIDAFMTKDREKGFDLQKDPLMRLALFDRGDSQYTCVWTHHHIIMDGWCLGIILKEFFSMYDSLKNNSPVQLGSTVP
PpsB-C            IFRTAFIYKN--VAKPRQVVLKQRHCPIHIEDISHLNERDKEHCTEAFKEQDKSKGFDLQTDVLMRISILKWAPDHYVCIWSHHHILMDGWCLGIVIKDFLHIYQALGKGQLPDLPPVQP
PpsC—D            IFRTIFISQN--VSSPQQVVLRERNVIVLEEDITHLNEAEQSQFIEQWKEKDRDRGFHLQKDVLMRIALIQTGESQYSCIWTFHHIMMDGWCLSIVLKEFLHIYASYVNASPITLEPVQP
PpsD—E            IFRTVFLPHVPHLSGPRQVVMTEREFHLNSEDISHLPTNDQNEYIERFKEKDKQKGFDLQKDMLMRISLFKTAKDEHVCIWSHHHILMDGWCLGIVMQEFMQIYQSIHAGKPLSLDPVRP
FegR-S            SLRAGFQRRS--SGDPVQLIRRRVVLPWREEDLSALPEEEALGEAERLSTEEQAQGFDMTVPPLLKVLLVKVGQDRYRMSVTLHHILLDGWSLPILMRELWTCY--EAGGSAEGLPAVTP
Tcp9-10           ALRASFHRRK--SGETVQLIPREVRLPWAERDLSGLPEKAALAEVGEIAAKERAERFDLTKPPLLRLMLIRLGPQRHCLVTTSHHLLMDGWSRAILESELLHVY--ASGGTVSGLPPAGS
                   :*                :           .                        : :      :  :            : **:  ***   ::       *
```

>>> Figure S1 continued on next page

B

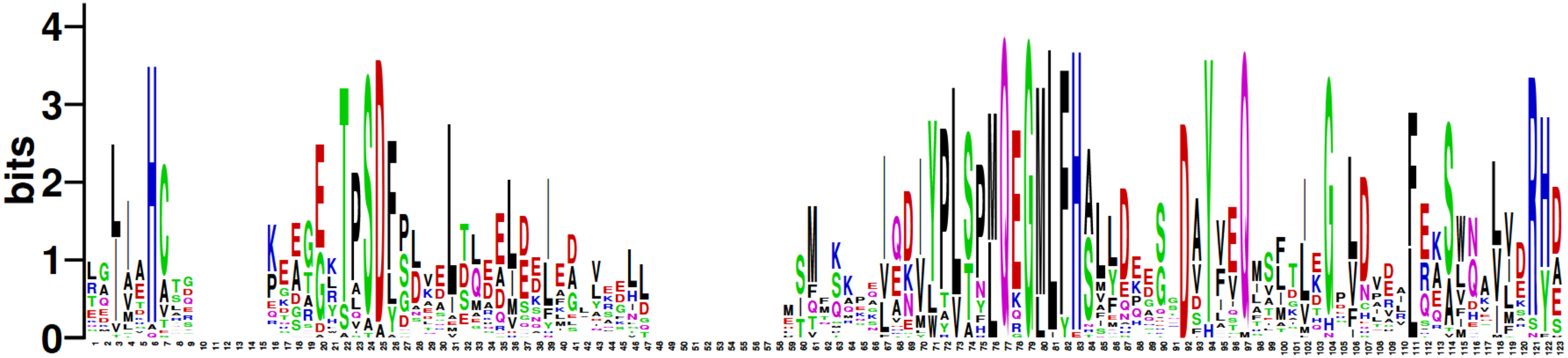

```
BacC4-5     KAVIRHCAE------REETEKTPSDYGDKGISLDQLEEIKLKYKG-------------------MEIEKIYPLANMQRGMLFHALEDKESQ-AYFEQMAINMKGLIDERLFAETFNDIMERHE
TycA-B      LAIIAHCTE------KKEVERTPSDFSVKGLQMEEMDDIFELLANTLR           MSVFSKEQVQDMYALTPMQEGMLFHALLDQEHN-SHLVQMSISLQGDLDVGLFTDSLHVLVERYD
TycB-C      LGIIEHCMA------KEEGEYTPSDLGDEELSMEELENILEWI                  MKKQENIAKIYPLTPLQEGMLFHAVTDTGSS-AYCLQMSATIEGDFHLPLFEKSLNKLVENYE
```

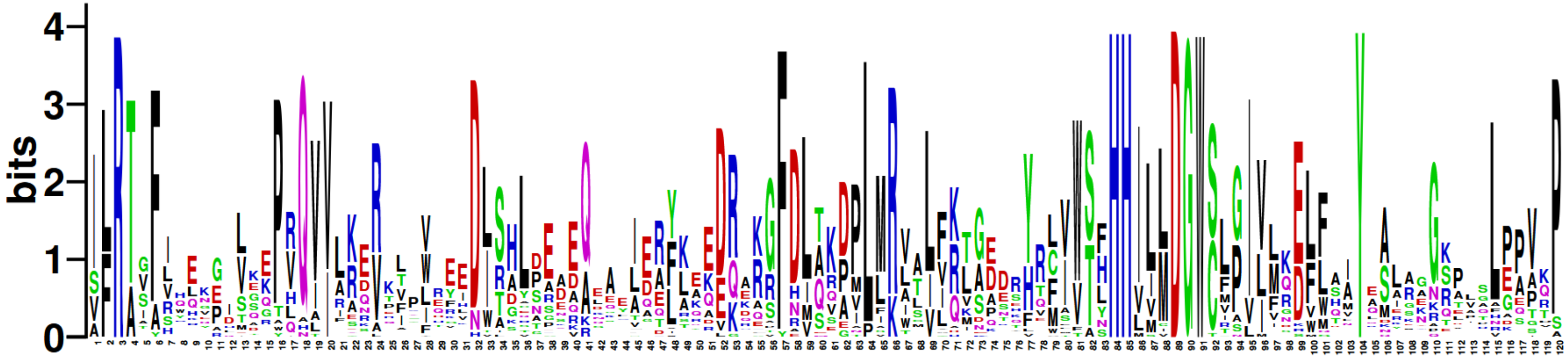

```
BacC4-5     ILR--ASIEYEITDEPRNVIIKDRKINLDYHDLRKQSPAEREQVIQAYRKADREKGFRLNSEPLIRAALMRTEDDSYTFIWTNHHILLDGWSRGIIMGELFHMYHMKEARQKHRLEEARP
TycA-B      VFRTLFLYEK--LKQPLQVVLKQRPIPIEFYDLSACDESEKQLRYTQYKRADQERTFHLAKDPLMRVALFQMSQHDYQVIWSFHHILMDGWCFSIIFDDLLAIYLSLQNKTALSLEPVQP
TycB-C      VLRTAFVYQN--MQRPRQVVFKERKVTVPCENIAHLPSAEQDAYIQAYTK--QHHAFDLTKDNLMKAAIFQTAENKYRLVWAFHHIIVDGWTLGVLLHKLLTYYAALRKGEPIPREATKP
```

Figure S1. Multiple sequence alignment of *cis*- and *trans*-COM domains. A) Shown are selected sequences aligned with CLUSTALW (see Table S3 for accession numbers). Structural elements of the *cis*- and *trans*-COM domains of BacC4-5 and TycA/TycB, respectively, are highlighted in bold and shaded letters. B) Sequence logo consensus representation sequence for all sequences shown in A) displayed on top of the representative *cis*-BacC4-5 and *trans*-TycA/TycB as well as *trans*-TycB/TycB sequences. Sequence logos were created using the Weblogo server at the University of Berkely (weblogo.berkeley.edu).[13]

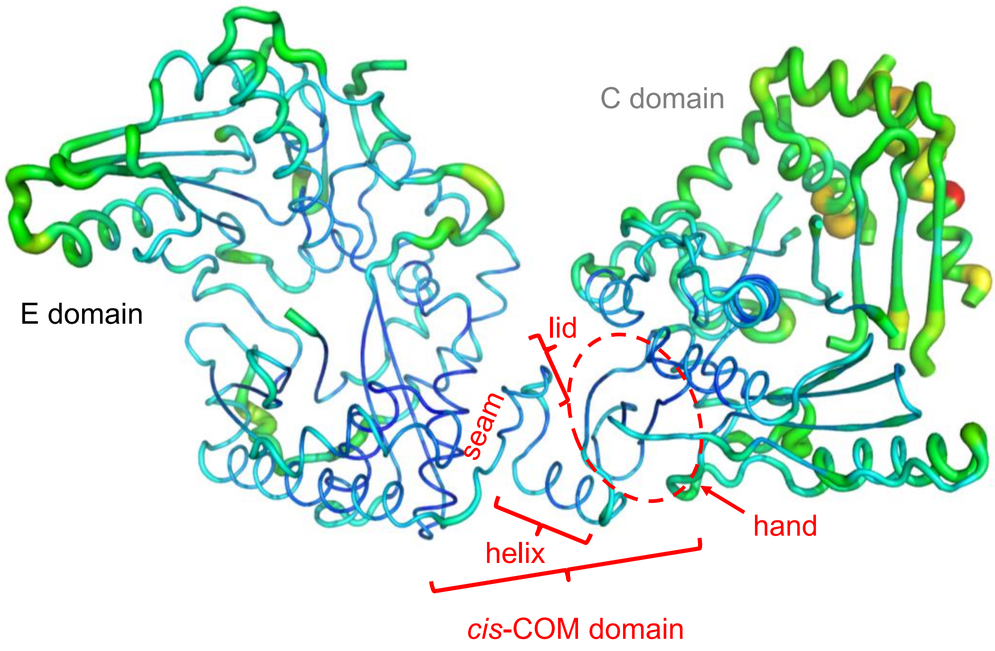

Figure S2. B-factor distribution. Cartoon representation of the E-COM-C structure in the same orientation as in Figure 2A, colored by B-factor distribution ranging from 50 Å² (blue) to 450 Å² (red).

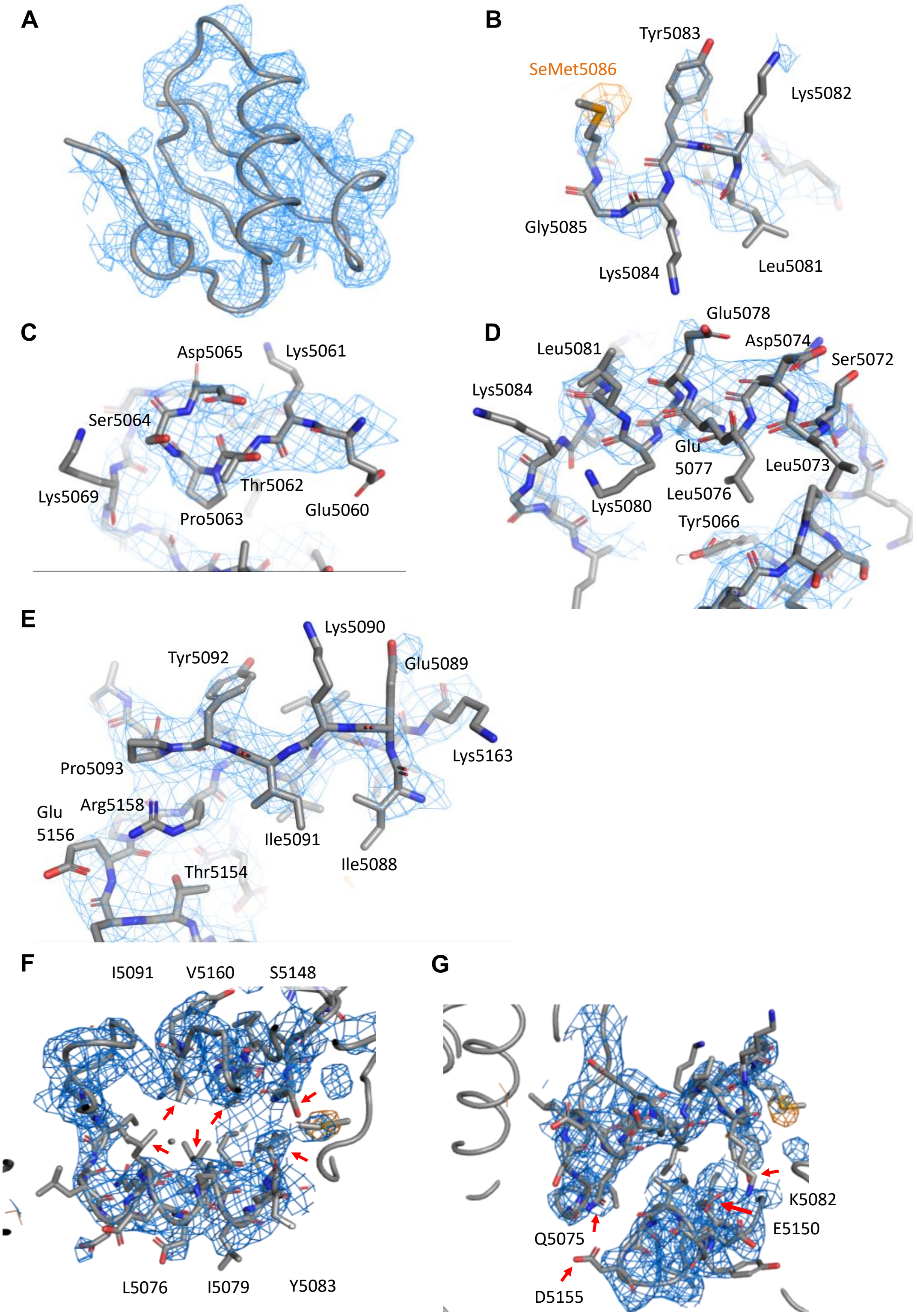

Figure S3. Map quality of the COM domain. Shown are composite omit maps (blue, contoured at 1.5 σ level) covering (A) the COM domain backbone trace in cartoon representation, (B) the linker between helix and thumb (representing the COM^{A}-COM^{D} linker in *trans*-COM domains), (C) seam/lid region, (D) helix, (E) a section of the hand region, as well as (F and G) selections of the helix-hand interaction in the same orientation as depicted in Figures 2B and 2C. Red arrows pinpoint the side chains of indicated residues. The orange map in (B) represents an anomalous difference map contoured at 3 σ level and defines the position of selenomethionine 5086.

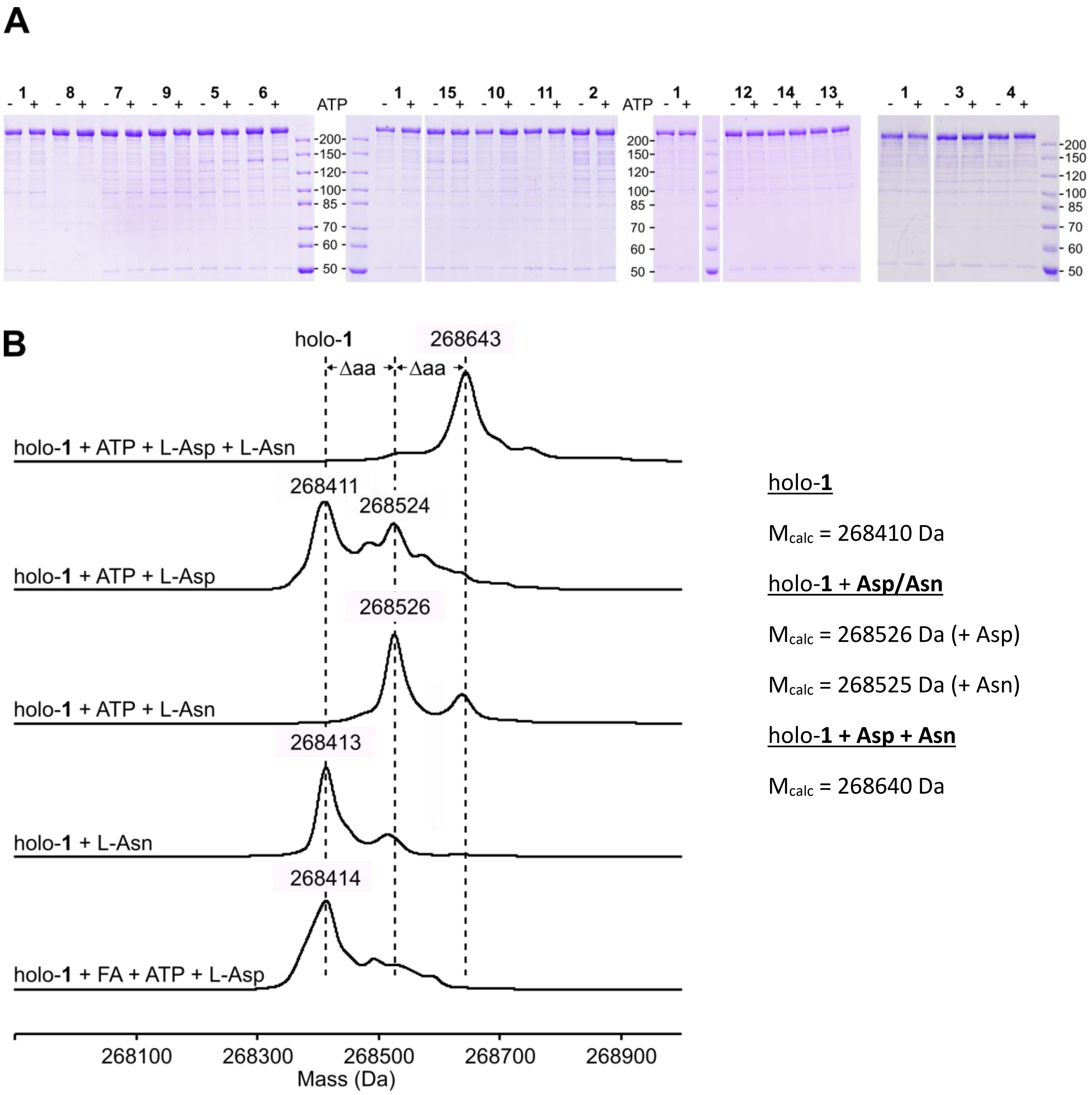

Figure S4. Dimodular BacC4-C5 constructs. A) Coomassie-stained 8% SDS PAGE gels of purified and Ppantylated holo-BacC4-C5 proteins **1** to **15**, with amino acids L-Asp and L-Asn and with or without ATP as indicated. These gels were run from removed aliquots of the dipeptide assays and also used to correct the amounts of formed dipeptide to the actual concentrations of the holo-BacC4-C5 proteins. B) Aminoacylation assay of holo-BacC4-C5 (**1**). Shown are mass spectra of the intact proteins (ESI-Q-TOF). Protein samples were ppantylated prior to the incubation with the respective substrates. Holo-**1** (7 µM) was then mixed with amino acids (2 mM each) and ATP (1 mM), as indicated, and incubated for 10 min. The protein concentration during the 10 min incubation period was 7 µM. Negative controls either lacked ATP or were acidified prior to the addition of ATP by addition of formic acid (FA) (quench control).

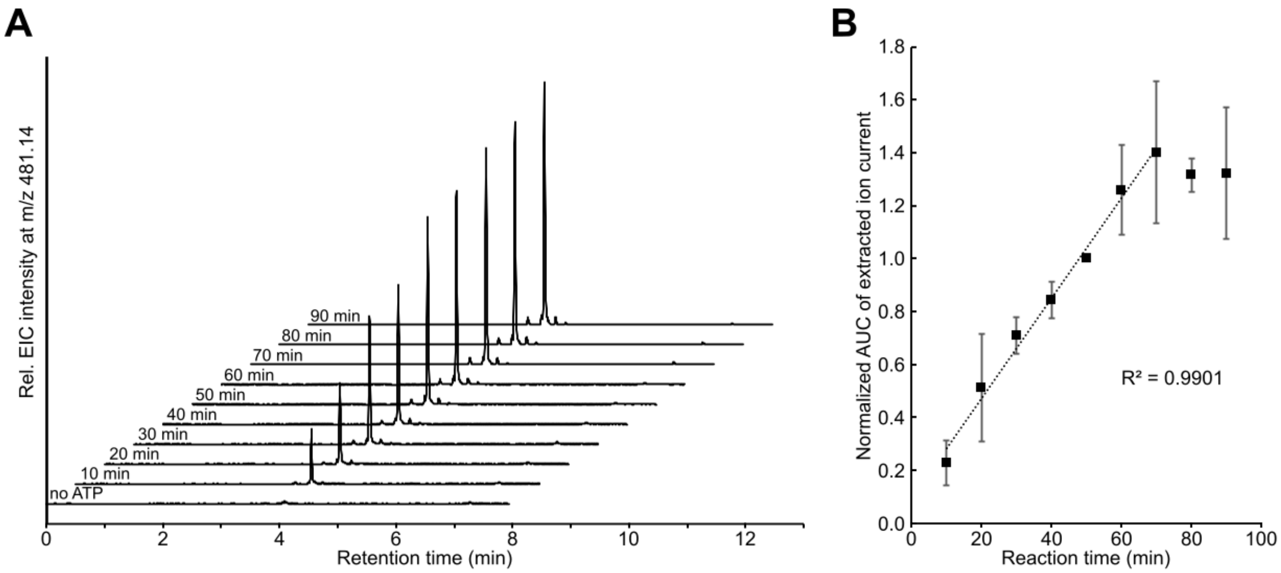

Figure S5. Time-course of product formation of holo-BacC4-C5 (**1**). A) EICs of $[M+H]^+$ of isobaric compounds with $C_{20}H_{24}N_4O_8S$ are shown, corresponding to the dansylated di-peptides D-Asp-L-Asn and L-Asp-L-Asn. Assay conditions: Incubation times were varied from 10 min to 90 min in the presence of 2 mM L-Asp, 2 mM L-Asn, 5 mM ATP (0 mM ATP in control) and 7 µM holo BacC4-C5 protein (**1**). After incubation, samples were quenched and dansylated for 45 min at 60°C. B) Averaged areas under the curve of three repeats of the experiment shown in A. AUCs show linearity with R^2 = 0.9901 for points between 10 min and 70 min. EIC = extracted ion count.

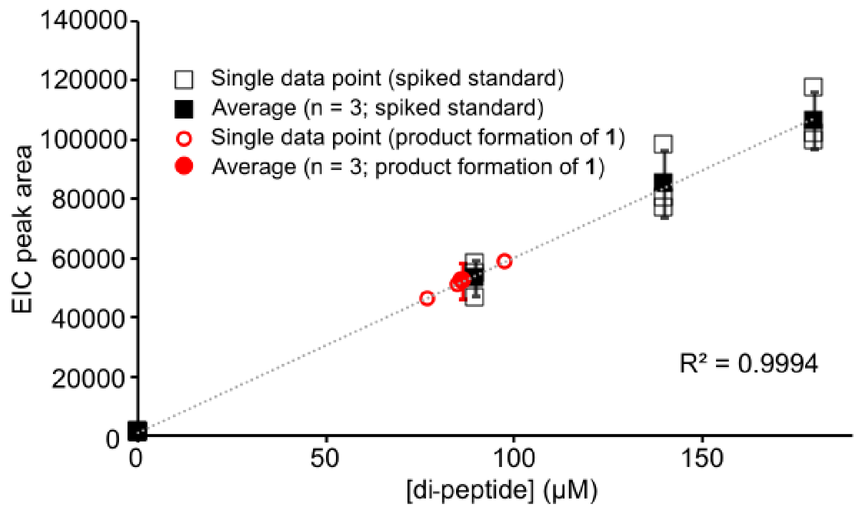

Figure S6. L-Asp-L-Asn standard calibration and product formation measurement. The dipeptide standard was spiked at 0 µM, 90 µM, 140 µM and 180 µM (open rectangles: single data points; filled black rectangles: average of of three technical repeats; error bars: standard error) into assay samples with 7 µM holo-BacC4-C5 WT (**1**), 2 mM L-Asp, 2 mM L-Asn lacking ATP. Enzyme activity samples contained no standard but 5 mM ATP (open circles: single data points; filled red circle: average of three technical repeats; error bar: standard error). Dansylation was carried out after 50 min incubation at 37°C. The whole calibration and product formation procedure was carried out three times. Within the incubation period holo-BacC4-C5 (**1**) produced 101.7 ± 22.3 µM L-Asp-L-Asn (n = 3), corresponding to about 1 molecule in 3 min per enzyme (0.29 min^{-1}), when calculating with the assumed enzyme concentration of 7 µM.

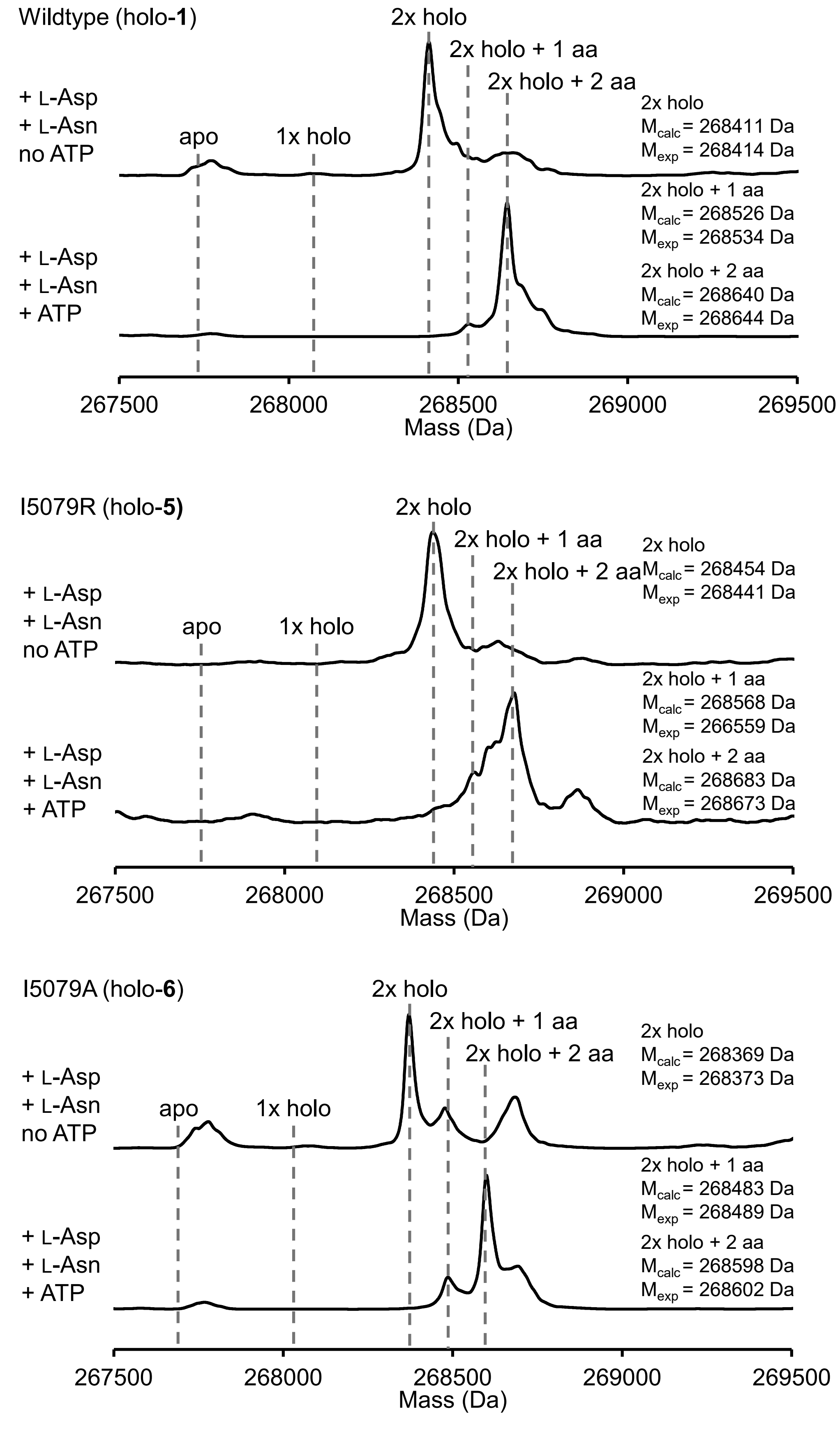

Figure S7 >>> continued

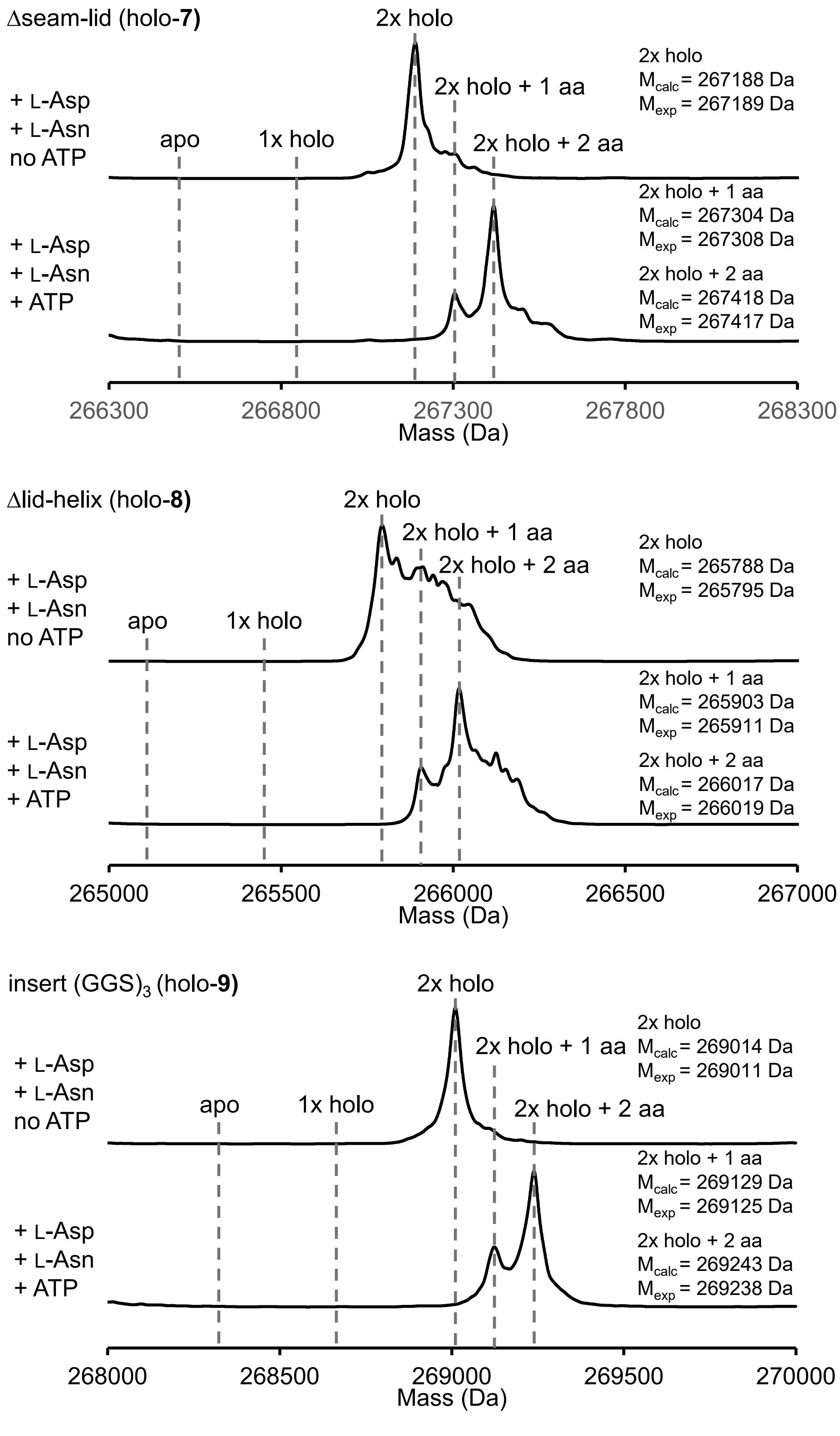

Figure S7 >>> continued

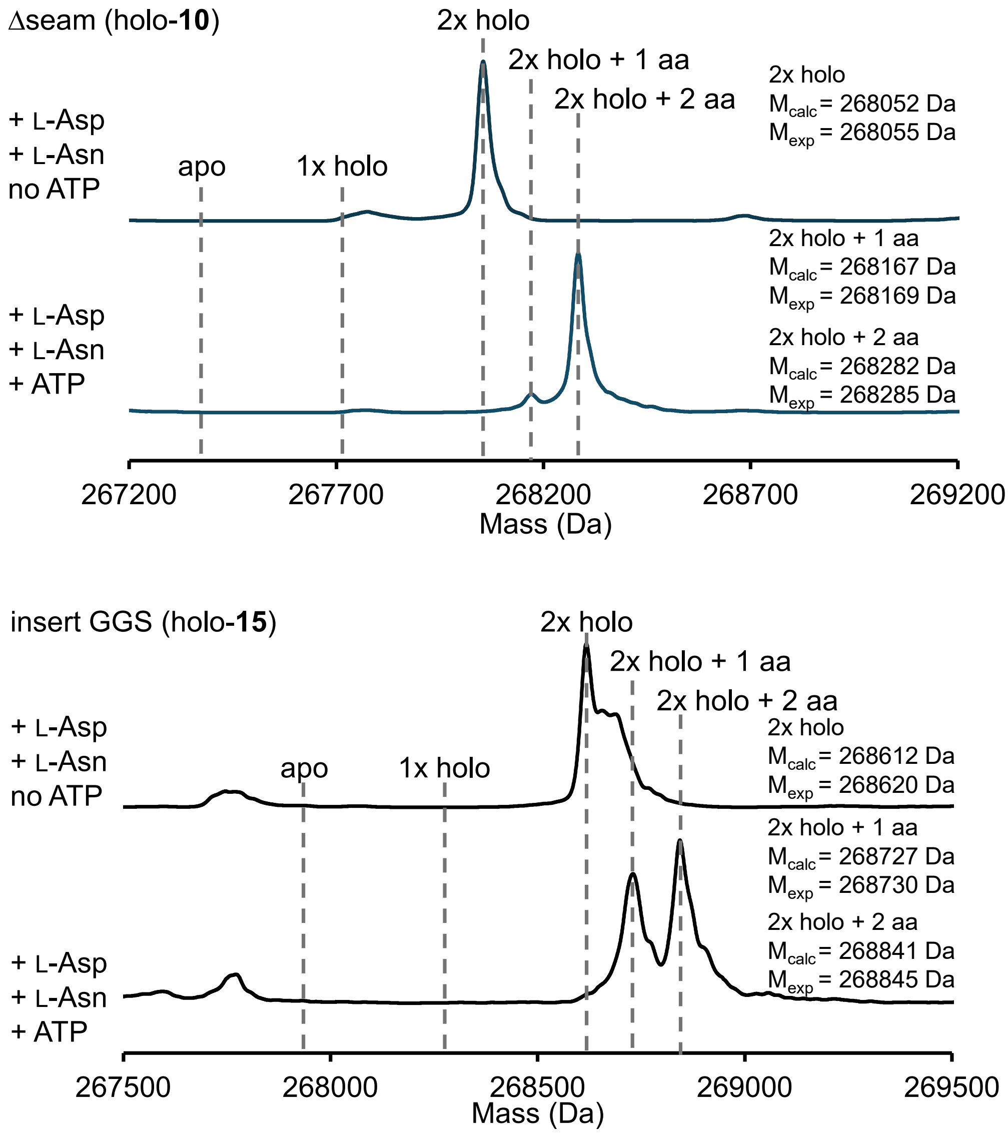

Figure S7. Aminoacylation activity of dimodular BacC4-C5 proteins and its COM mutants. The indicated proteins were analyzed by ESI-MS as ppantylated holo-forms obtained by a 40 min treatment of the purified proteins with Sfp and CoASH and followed by a 10 min incubation period with 2 mM of both, L-Asp and L-Asn, either with or without 1 mM ATP. These data show that all investigated mutants retained most (approximately 60% up to nearly 100%) of the aminoacylation activity of the wildtype protein **1**. Importantly, after incubation with ATP + L-Asp + L-Asn virtually no mass corresponding to the uncharged holo-protein can be detected anymore, suggesting that no inactive protein is present in each of the preparations. The only additional species that can be detected to certain degrees in some cases is the singly aminoacylated protein. Most importantly, the aminoacylation activities look similar for those BacC4-C5 proteins that show high activity in dipeptide formation (wildtype **1** and mutants **6** & **15**) and for those that are heavily impaired in dipeptide formation (mutants **5**, **7**, **8**, **9**, and **10**). Together, these data suggest that the significant activity drop in L-Asp-L-Asn-dipeptide formation seen for several COM-mutant proteins indeed stems from the local COM-mediated defects and not from losses in activity associated with the A and PCP domains, consistent with the semi-autonomous character of the catalytic domains. Note that the chemical lability of the aminoacyl-thioesters complicates the accuracy of this analysis and that in this mass range no distinction between the aspartyl- and asparaginyl-thioesters can be made.

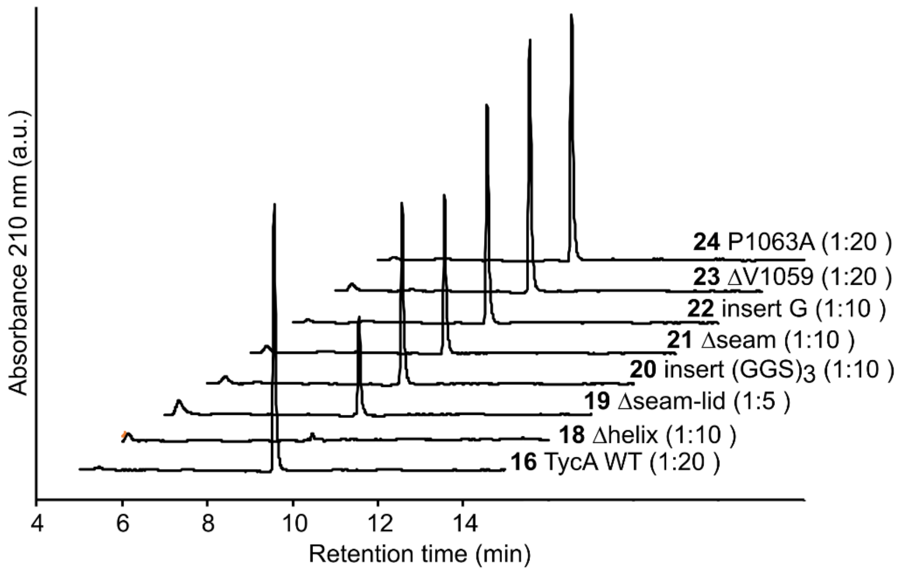

Figure S8. HPLC chromatograms of DKP formation of TycA WT and indicated mutants with TycB1. Numbers in brackets indicate the dilutions (in HPLC buffer), with which the samples were analyzed by HPLC.

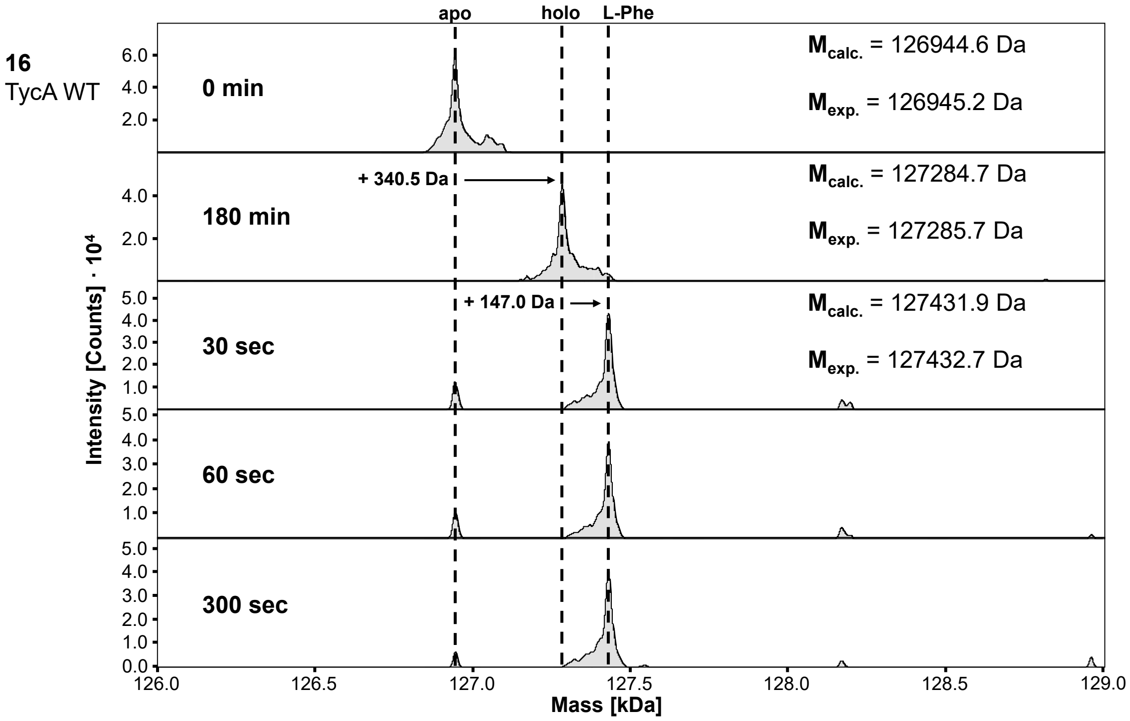

Figure S9 >>> continued

18
Δhelix

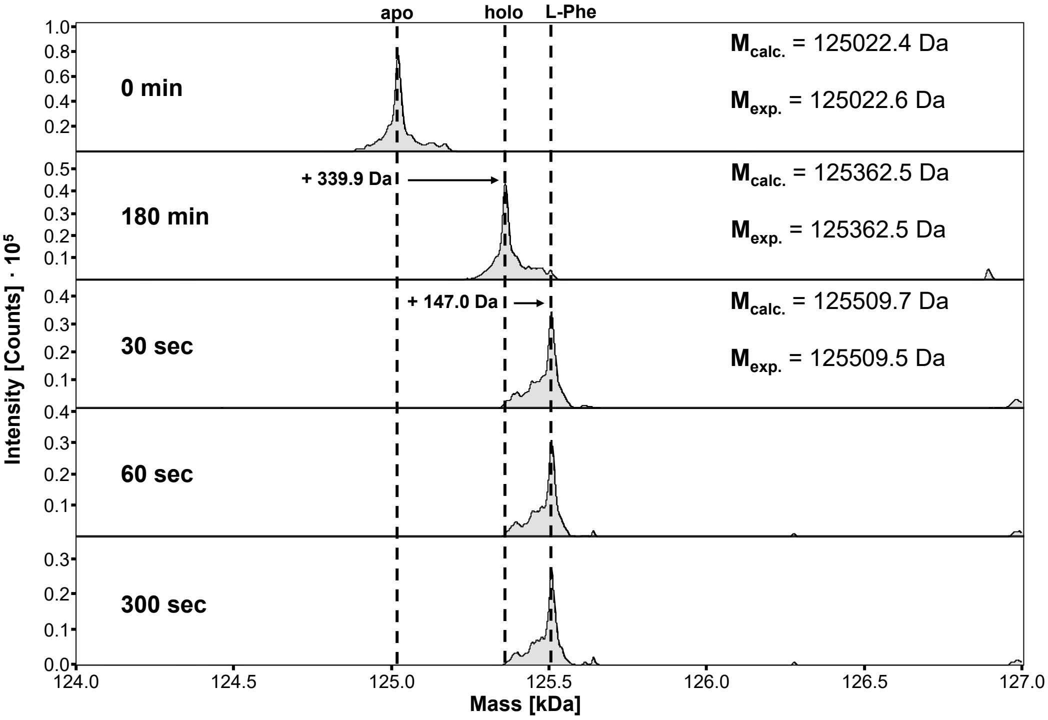

19
Δseam-lid

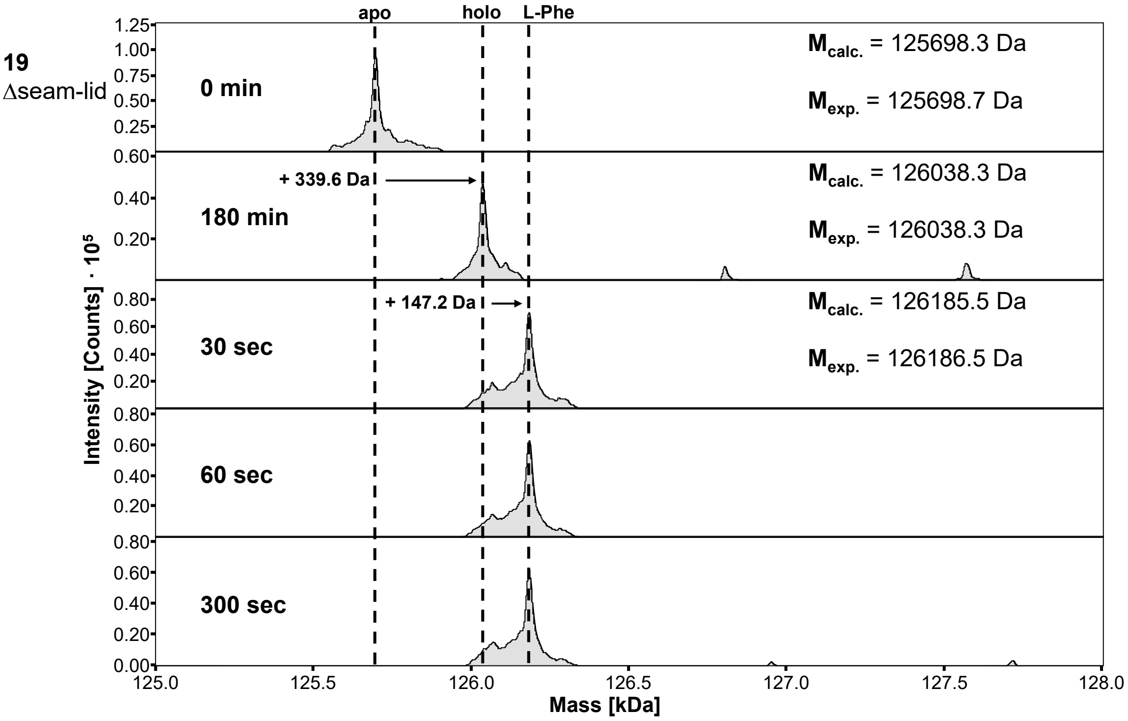

Figure S9 >>> continued

20
insert
$(GGS)_3$

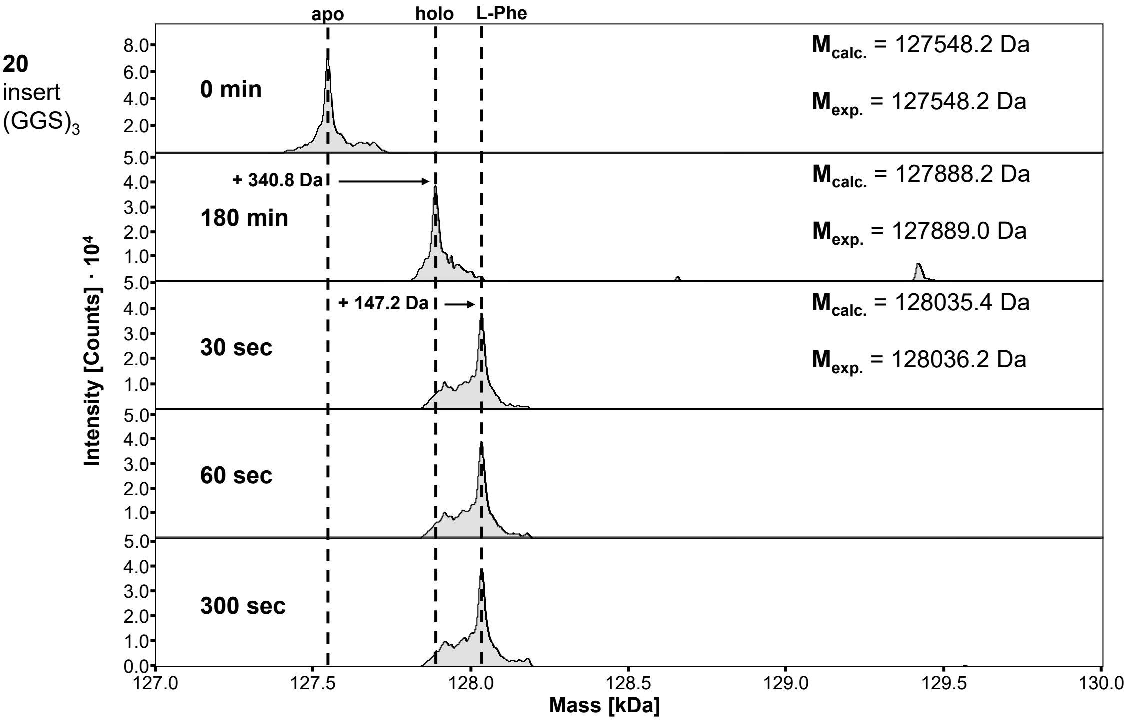

21
Δseam

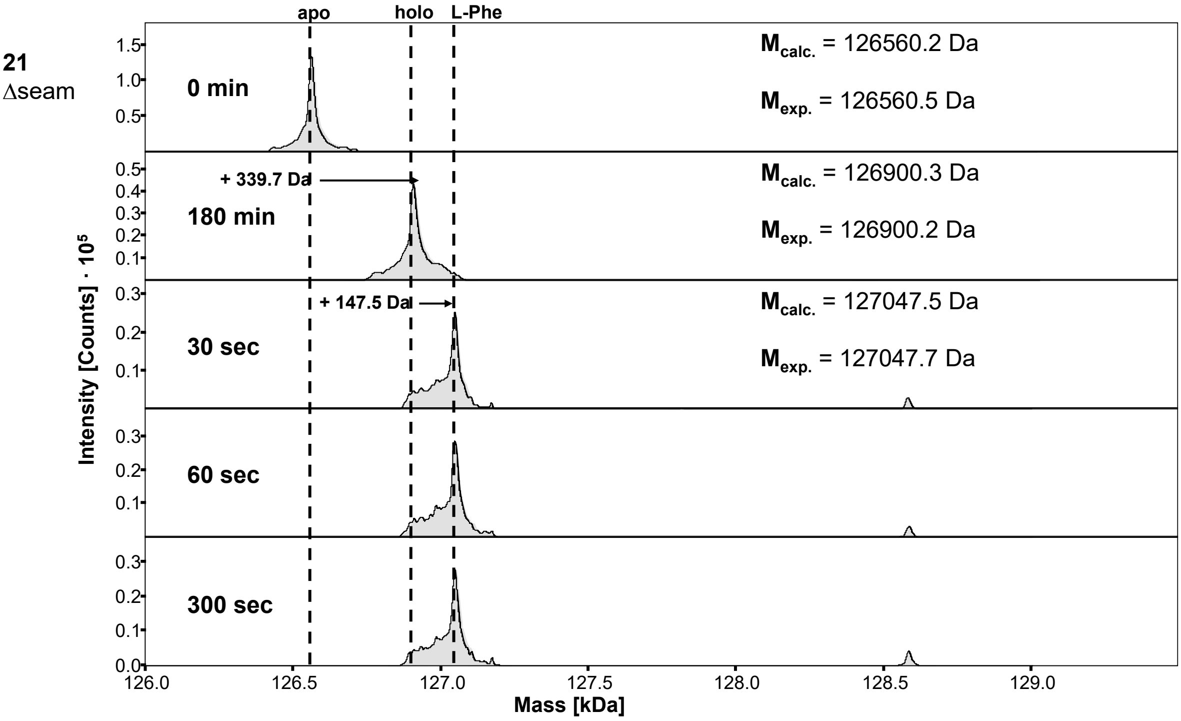

Figure S9 >>> continued

22
insert G

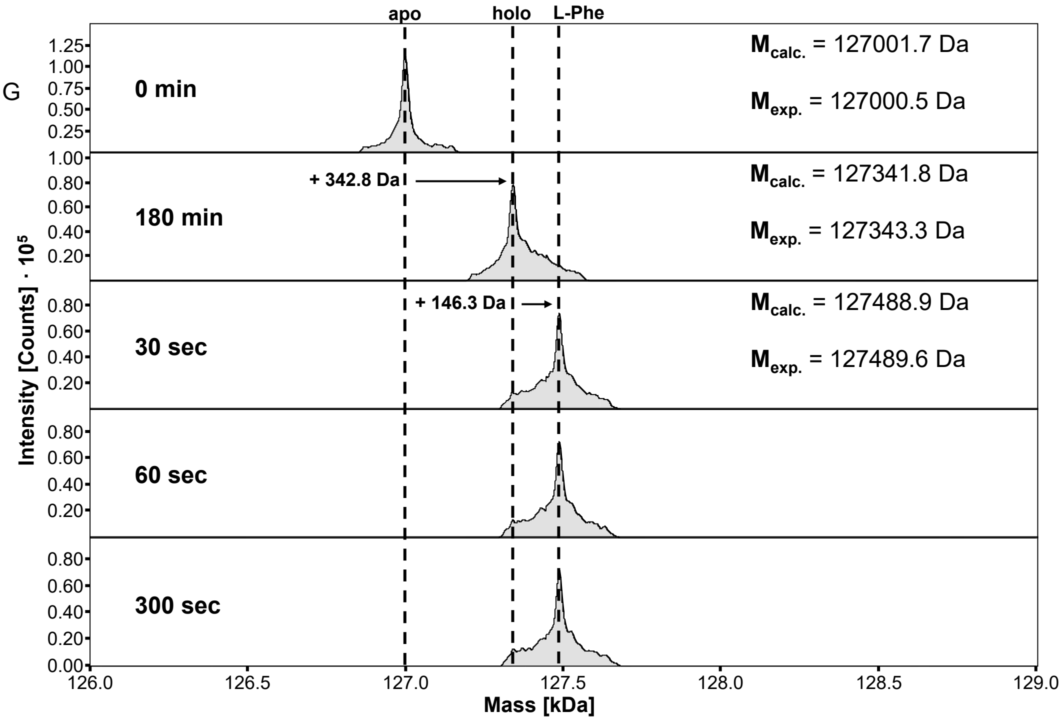

23
ΔV1059

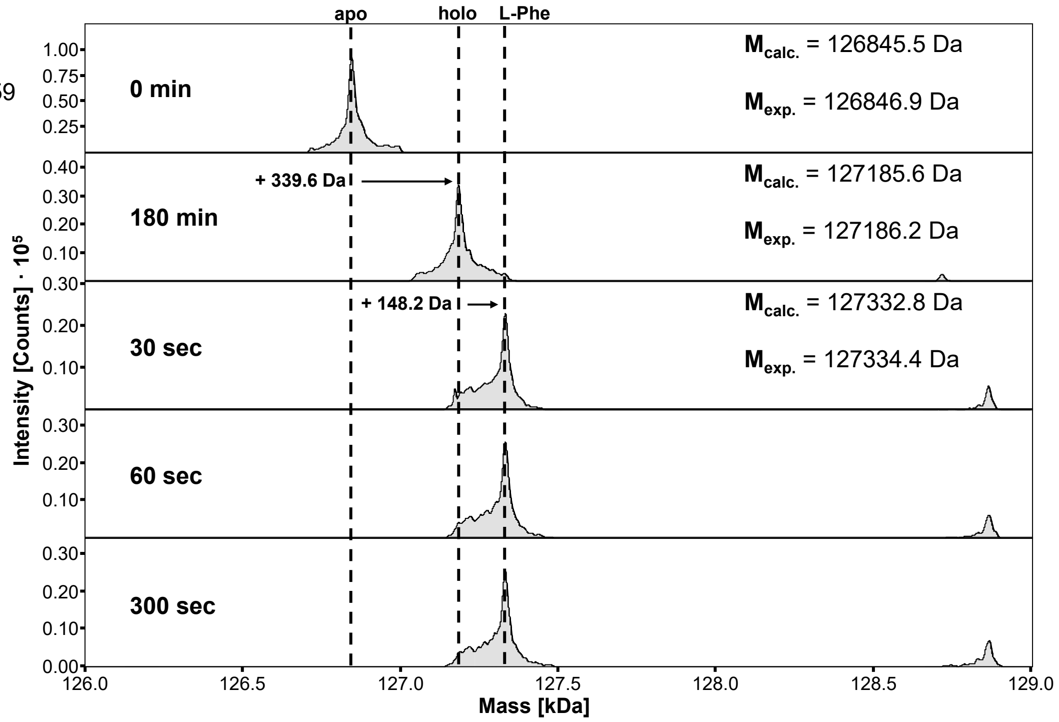

Figure S9 >>> continued

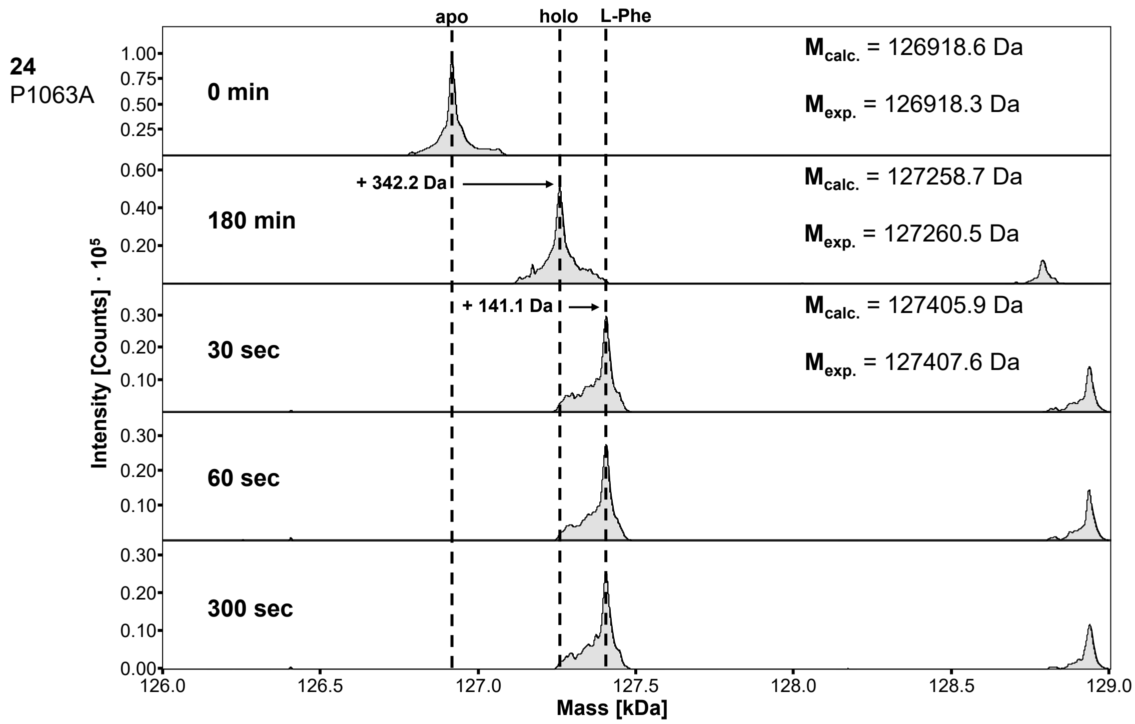

Figure S9. Aminoacylation activity of SBP-TycA (**16**) and its COM mutants **18**-**24**. The indicated proteins were analyzed by ESI-MS as purified apo-proteins (top spectra; 0 min), following 3 h of 4'-Ppantylation with Sfp and CoASH to convert them into the holo-forms (second spectra from top; 180 min), and at 3 time points after addition of ATP and L-Phe to following the formation of the aminoacyl thioester (three spectra from bottom; at 30 sec, 60 sec, and 300 sec). These data show that all mutants retained the virtually full aminoacylation activity of the wild-type protein **16** after 30 sec.

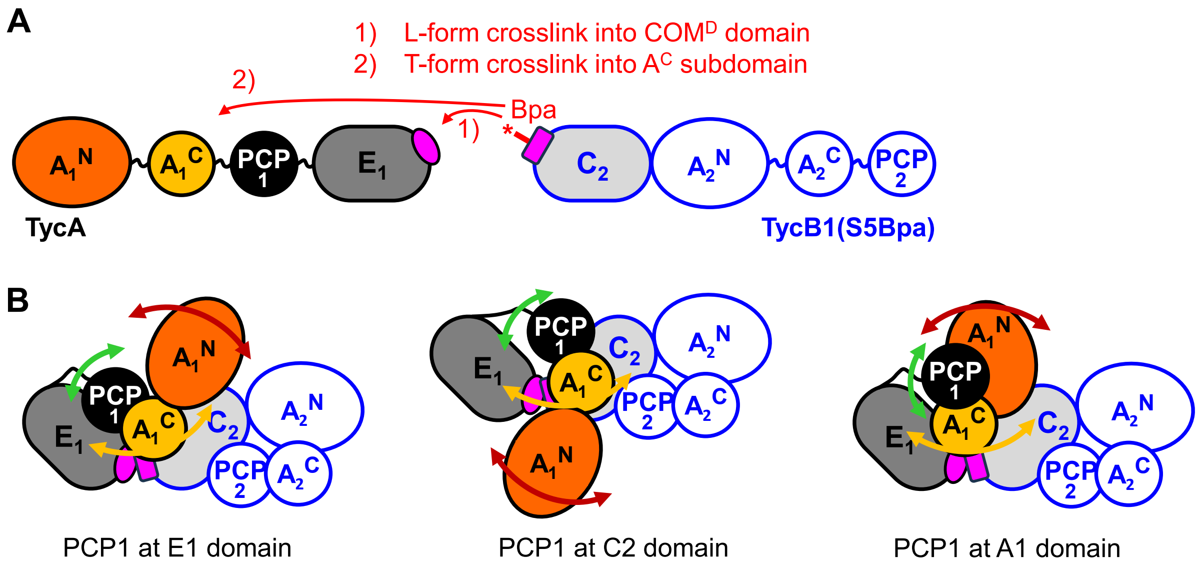

Figure S10. L- and T-crosslink formation in photo-crosslinking between TycA and TycB1. A) Linear representation of the multi-domain TycA and TycB1 module with the photo-crosslinking amino acid Bpa inserted at position S5 of TycB1, corresponding to the thumb region of the COM^A domain. Photo-crosslinking results in proximity-driven covalent bond formation with 1) either the COM^D domain of TycA or 2) the A^C subdomain of TycA. B) Illustration of theoretically possible 3-D domain arrangements in the associated

TycA/TycB1 protein complex to explain the spatial proximity between the A^C subdomain of TycA and the COM^A of TycB1 domain, giving rise to the T-form crosslink. Three different complexes depict the interaction of PCP1 with each of its catalytic domain partners. With a PCP1 positioning in the interspace between E1 and C2 domain, as supported by the E-COM-C structure, the spatial proximity of the A^C1 domain to S5Bpa, which would explain the T-form crosslink, is plausible in each case given the dimensions of the domains and the flexibility of the linkers between them. Note that the preferred exact location, if any, of the A^N1 and A^C1 domains in general is not known. Likewise, the positioning of the PCP1 domain relative to the E domain cannot be extrapolated from known structures in case of the PCP1-A^N1/A^C1 interaction (right panel).

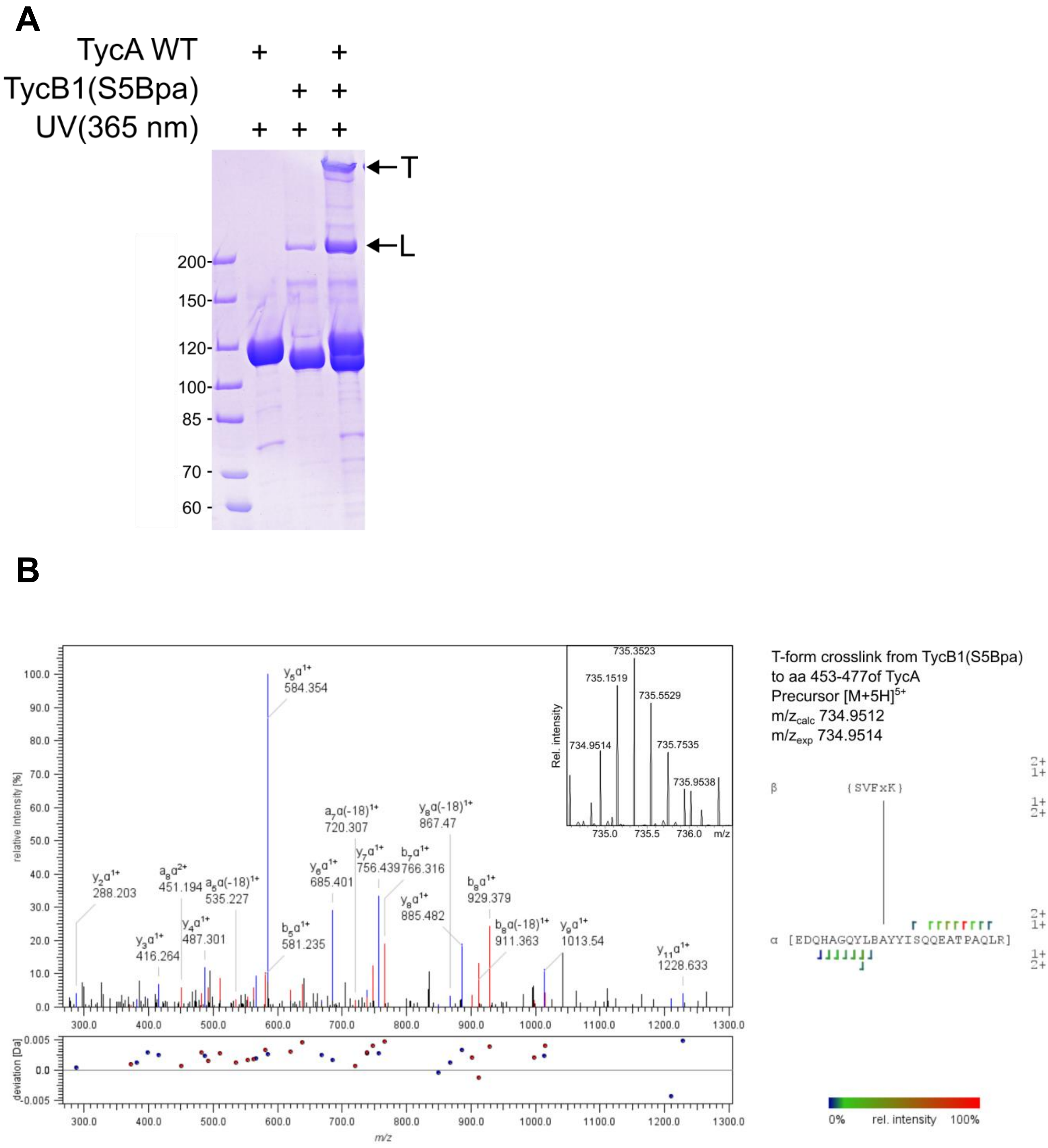

Figure S11 >>> continued

C

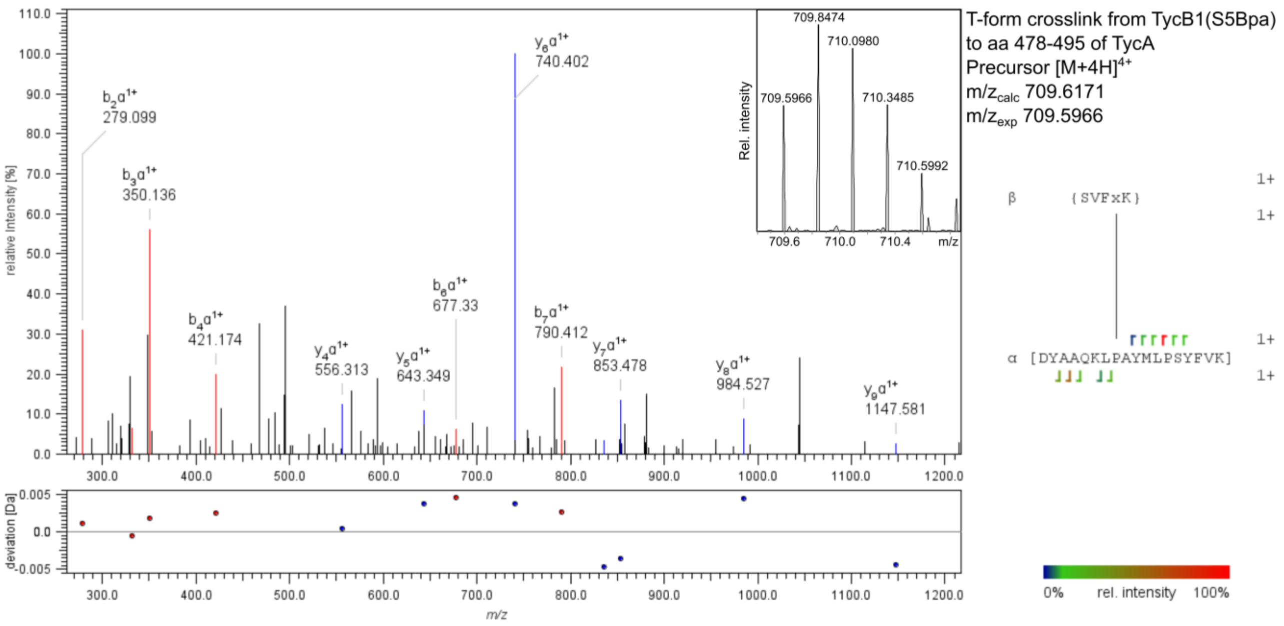

D

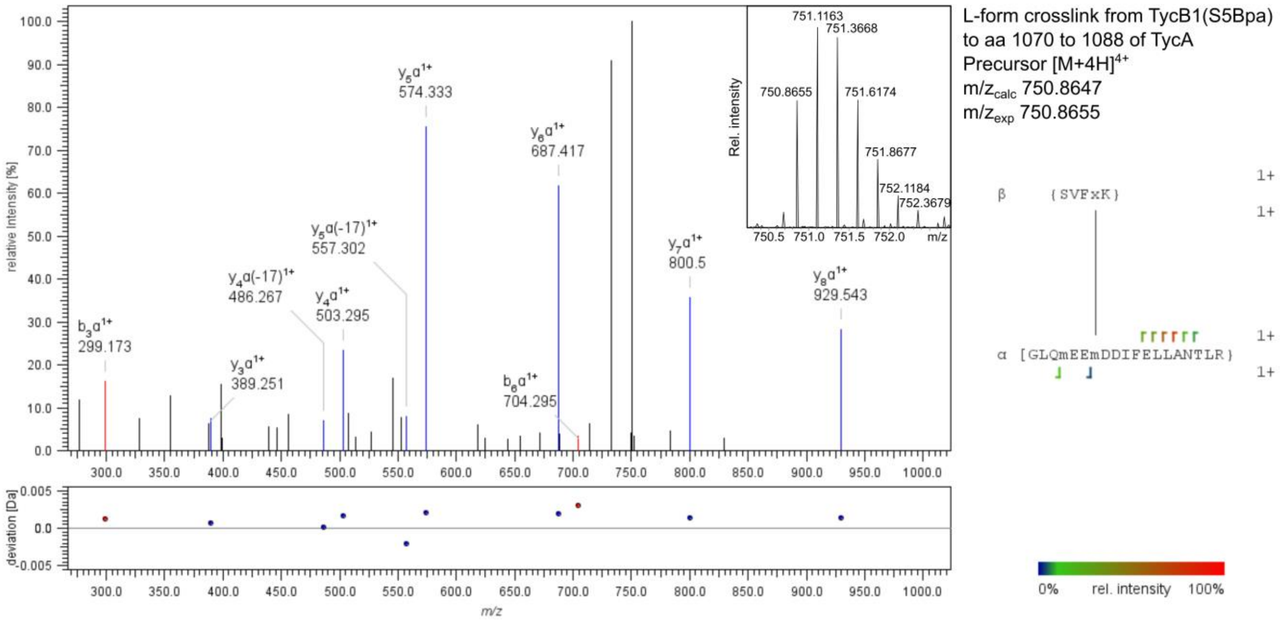

Figure S11. Mapping of photocrosslinks between TycB1 and TycA. A) SDS-PAGE gel of UV-irradiated Tyc module proteins and mixtures thereof. B-D) MSMS spectra of mapped crosslink peptides from tryptic digestion of TycA / TycB1 S5Bpa crosslink gel bands as indicated by arrows in A). General remarks: The corresponding precursors are shown in the upper-right insets of each MSMS spectrum. Crosslink peptides are schematically depicted next to each MSMS spectrum. In the amino acid sequences, “x” stands for the crosslinker Bpa, “B” denotes carbamidomethyl cysteine and “m” indicates oxidized methionine. Fragment ions flanking amino acids are indicated by half-square brackets. Due to the incomplete coverage of detectable fragment ions, there is a range of potential binding partners delimited by brackets rather than one amino acid position. Panel B) shows an identified mapped crosslink from the excised T-crosslink band into the

peptide aa453-477 (EDQHAGQYLBAYYISQQEATPAQLR) of TycA, which corresponds to a part of the A^C subdomain of TycA. Panel C) shows an identified crosslink from the excised T-crosslink band into the peptide aa478-495 (DYAAQKLPAYMLPSYFVK) of TycA, which also corresponds to a part of the A^C subdomain of TycA. Panel D) shows an identified crosslink from the excised L-crosslink band into peptide aa1070-1089 (GLQmEEmDDIFELLANTLR) of TycA, which corresponds to the seam and helix region of the COM^D domain of TycA.

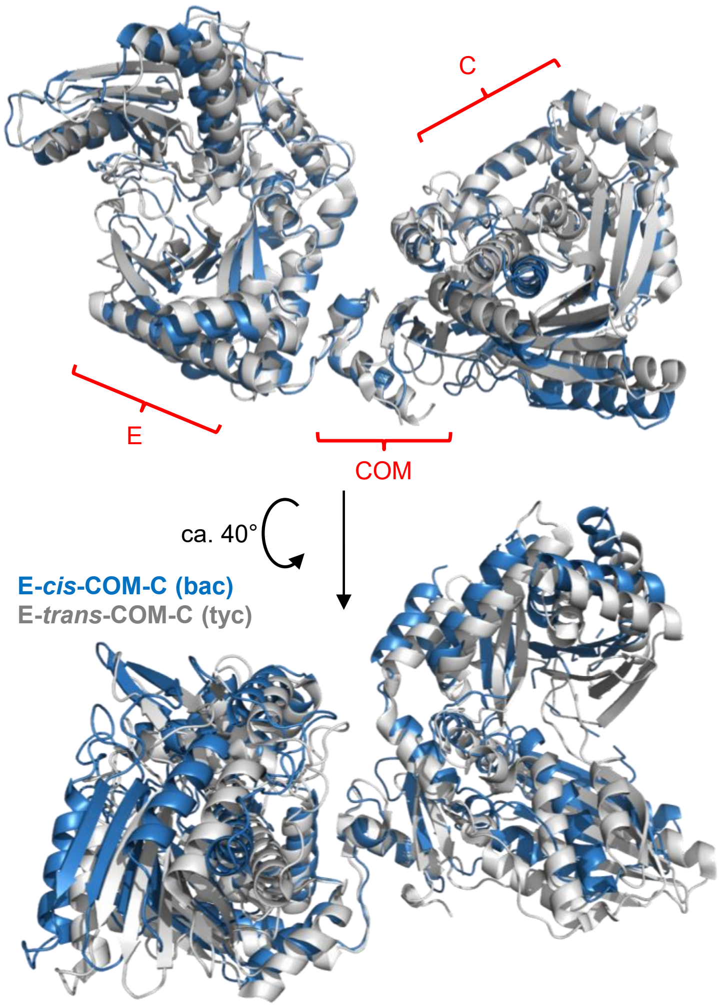

Figure S12. Comparison von *cis*-COM and *trans*-COM ensembles with neighboring E and C domains. Crystal and cryo-EM structures depicting the E-*cis*-COM-C (this study) and E-*trans*-COM-C structures (pdb-code: 9BFD, Ref[14]), respectively, were overlayed using the COM^D parts. Note that the positioning of the E and C domains relative to one another is highly similar for both domain ensembles, further underlining the conserved structural role of the COM domain in orienting its neighboring domains.

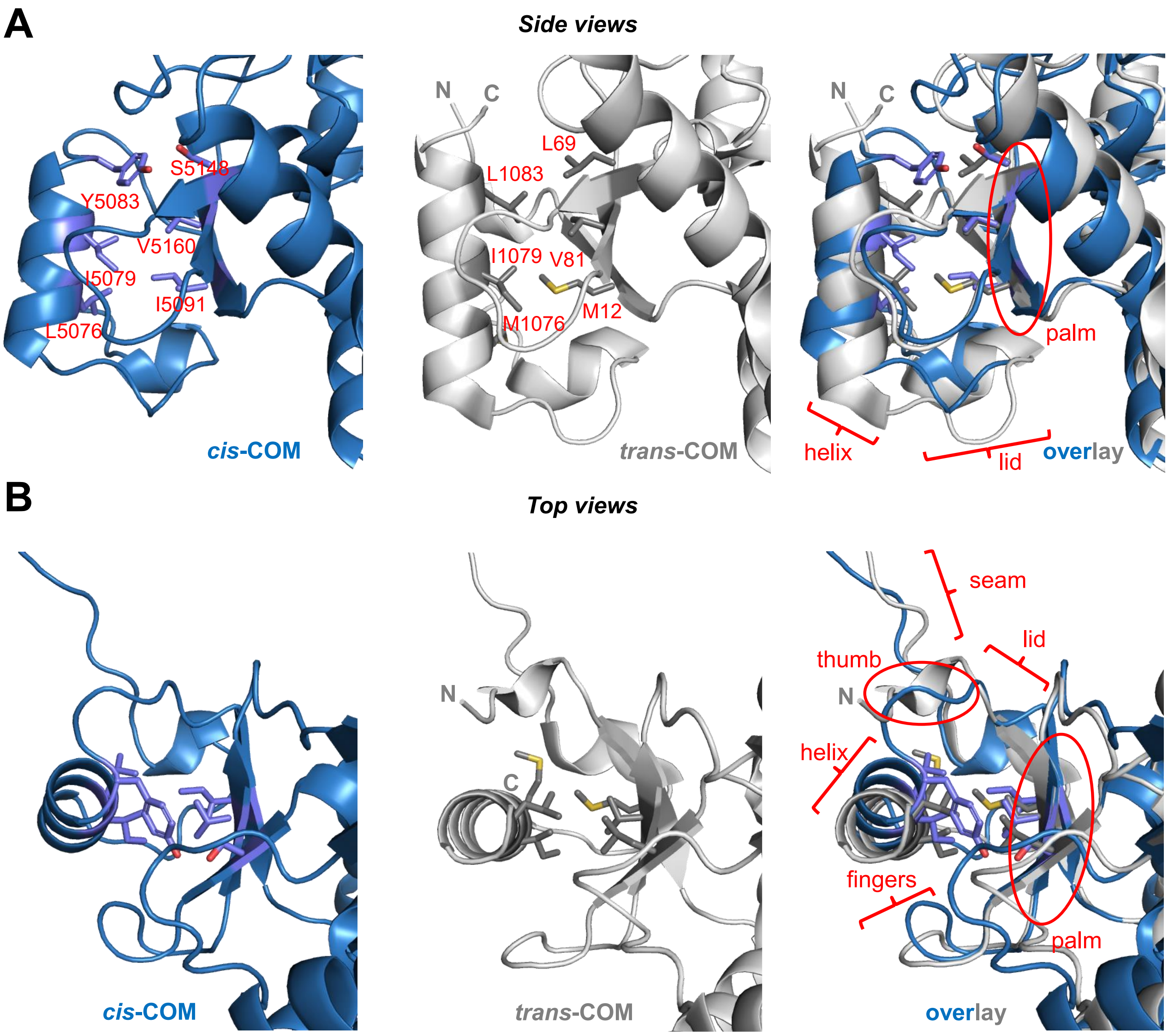

Figure S13. Binding modes in helix-hand interaction. Shown are parts of the crystal and cryo-EM structures depicting the *cis*-COM (this study) and *trans*-COM structures (pdb-code: 9BFD, Ref[14]), respectively. In the illustrations the COM domains are shown starting at the beginning of the seam region (the N-terminally located E domains are not shown) and with most of the body of the C-terminally located C domains being cut off at the right-hand sides of the panels. The central three residues from both the helices and the palm regions of each helix-palm interface are highlighted as stick representations of the side chains with the corresponding residue numbers given in the two upper left panels. The overlayed representations (right panels) were created by overlaying all residues of the three palm β-sheets from the two structures to reveal how the helices are oriented relative to the palm region. Note that the two helices are in differently staggered arrangement relative to their palm interaction interface and show a slightly different tilt of their helix axis relative to the palm region. Together with the additional helix turn found for the central helix of TycA (*trans*-COM), this arrangement results in slight differences of the positioning of the N-terminal ends of the two helices and slight differences in the folding of the lid regions. These differences might represent minor folding differences within COM domains, in particular when comparing COM domains with varying numbers of amino acids (see Figure S1). A) Side views with angle perpendicular to the central α-helices. B) Top views along the helix axis with the N-terminal end of the helices facing the viewer. Images were created using PyMol.

Supporting Tables

Table S1. Diffraction data collection and refinement statistics.

**Wavelength (Å)**	0.979510
**Space group**	$P4_1$
**Unit cell** **a, b, c (Å)**<br>**α, β,γ (°)**	147.749, 147.749, 66.5<br>90 , 90, 90
**Resolution range**	49.42 – 3.29 (3.48-3.29)
**Total reflections**	298,579 (45,532)
**Unique reflections**	42,422 (6,884)
**Multiplicity**	7.04 (6.61)
**Anomal Corr**	53 (2)
**SigAno**	1.31 (0.67)
**Completeness (%)**	99.7 (98.3)
**I / sigma**	9.55 (1.43)
**R-meas (%)**	12.5 (116.4)
**R-pim (%)**	4.71 (45.3)
**CC ½**	0.996 (0.626)
**CC***	0.999 (0.877)
**Wilson B factor**	129.44
**Refinement**	49.42 – 3.29
**Space group**	P4 (1)
**Reflections in refinement**	40297
**Reflections in free set**	2110
**Rwork**	0.2435
**Rfree**	0.3013
**RMSD bonds**	0.0013
**RMSD angles**	0.49
**Ramachandran favoured (%)**	97.28
**Ramachandran allowed (%)**	2.72
**Ramachandran outliers (%)**	0.0
**Rotamer outliers**	0.0
**Clash score**	2.76
**B-factor (min/max/mean)**	51.77/352.73/140.57
**MolProbity score**	1.20

Statistics for the last shell is given in parentheses. Refinement statistics were calculated with MolProbity and phenix_refine.

Table S2. List of purified recombinant proteins and the respective expression plasmids.

No.	Protein	MW (kDa)	Encoding plasmid	Vector backbone
	BacC4-C5[E-COM-C]-His_6	106.366	pAF24	pET28a
1	BacC4-C5[A-PCP-E-COM-C-A-PCP-TE]-His_6	267.730	pJD01	pET28a
2	BacC4-C5[A-PCP-E-COM-C-A-PCP-TE(S6204A)]-His_6	267.714	pJD16	pET28a
3	BacC4-C5[A-PCP-E(H4755A)-COM-C-A-PCP-TE]-His_6	267.664	pJD22	pET28a
4	BacC4-C5[A-PCP-E(E4893A)-COM-C-A-PCP-TE]-His_6	267.672	pJD23	pET28a
5	BacC4-C5[A-PCP-E-COM(I5079R)-C-A-PCP-TE]-His_6	267.773	pJD10	pET28a
6	BacC4-C5[A-PCP-E-COM(I5079A)-C-A-PCP-TE]-His_6	267.688	pJD11	pET28a
7	BacC4-C5[A-PCP-E-COM(Δ5059-5069)-C-A-PCP-TE]-His_6	266.508	pJD08	pET28a
8	BacC4-C5[A-PCP-E-COM(Δ5062-5084)-C-A-PCP-TE]-His_6	265.107	pJD07	pET28a
9	BacC4-C5[A-PCP-E-COM(Ins5062(GGS)$_3$)-C-A-PCP-TE]-His_6	268.334	pJD09	pET28a
10	BacC4-C5[A-PCP-E-COM(Δ5059-5061)-C-A-PCP-TE]-His_6	267.372	pJD14	pET28a
11	BacC4-C5[A-PCP-E-COM(Ins5062G)-C-A-PCP-TE]-His_6	267.787	pJD15	pET28a
12	BacC4-C5[A-PCP-E-COM(Δ5059)-C-A-PCP-TE]-His_6	267.629	pJD19	pET28a
13	BacC4-C5[A-PCP-E-COM(Ins5068G)-C-A-PCP-TE]-His_6	267.787	pJD21	pET28a
14	BacC4-C5[A-PCP-E-COM(P5063A)-C-A-PCP-TE]-His_6	267.704	pJD20	pET28a
15	BacC4-C5[A-PCP-E-COM(Ins5086GGS)-C-A-PCP-TE]-His_6	267.931	pJD13	pET28a
16	SBP-TycA[A-PCP-E-COM^D]	126.945	pJR89	pET28a
17	TycB1[COM^A-C-A-PCP]-His_6	119.696 (excluding Met1)	pJR95 (Ref[2])	pTrc99a
18	SBP-TycA[A-PCP-E-COM^D(ΔHlx)]	125.022	pBH86	pET28a
19	SBP-TycA[A-PCP-E-COM^D(ΔLinker)]	125.698	pBH81	pET28a
20	SBP-TycA[A-PCP-E-COM^D(VER(GGS)$_3$TPSD)]	127.548	pBH82	pET28a
21	SBP-TycA[A-PCP-E-COM^D(ΔLinker_sm)]	126.560	pBH83	pET28a
22	SBP-TycA[A-PCP-E-COM^D(VERgTPSD)]	127.002	pBH88	pET28a
23	SBP-TycA[A-PCP-E-COM^D(ΔV1059)]	126.845	pBH85	pET28a
24	SBP-TycA[A-PCP-E-COM^D(P1063A)]	126.919	pBH89	pET28a
25	TycB1[COM^A-C(S5X)-A-PCP]-His_6 (with S5Bpa)	119.860 (excluding Met1)	pBH96	pTrc99a

Table S3. Accession numbers of protein sequences used in Figure S1

**Short name of NRPS(s)**	**Designation in Figure S1 (listed here in the same order of appearance)**	**Full name of NRPS(s)**	**NCBI Protein Bank accession number**
BacC	BacC4-5, BacC2-3	Bacitracin synthetase 3	AAC06348
BacA	BacA4-5	Bacitracin synthetase 1	AAC06346
LgrB	LgrB2-3	Linear gramicidin A synthetase B	Q70LM6
LgrC	LgrC2-3, LgrC4-5	Linear gramicidin A synthetase C	Q70LM5
LgrD	LgrD2-3	Linear gramicidin A synthetase D	Q70LM4
FegR	FegR2-3, FegR4-5	Feglymycin synthetase R	ALK27915
FegQ	FegQ2-3, FegQ1-2	Feglymycin synthetase Q	ALK27914
DptA	DptA2-3	Daptomycin synthetase A	AAX31557
Tcp11	Tcp11_4-5, Tcp11_5-6	Teicoplanin synthetase 3	CAG15011
TycA + TycB	TycA-B	Tyrocidine synthetases 1 and 2	AAC45928 and T31075
TycB + TycC	TycB-C	Tyrocidine synthetases 2 and 3	T31075 and T31076
GrsA + GrsB	GrsA-B	Gramicidin S synthetases 1 and 2	P0C061 and P0C063
SrfAA + SrfAB	SrfAA-AB	Surfactin synthetases 1 and 2	AIX06065 and AIX06066
SrfAB + SrfAC	SrfAB-AC	Surfactin synthetases 2 and 3	AIX06066 and AIX06067
LicA + LicB	LicA-B	Lichenysin synthetases A and B	AAD04757 and AAD04758
LicB + LicC	LicB-C	Lichenysin synthetases B and C	AAD04758 and
BacB + BacC	BacB-C	Bacitracin synthetases 2 and 3	AAC06347 and AAC06348
LgrA + LgrB	LgrA-B	Linear gramicidin A synthetases A and B	Q70LM7 and Q70LM6
LgrB + LgrC	LgrB-C	Linear gramicidin A synthetases B and C	Q70LM6 and Q70LM5
LgrC + LgrD	LgrC-D	Linear gramicidin A synthetases C and D	Q70LM5 and Q70LM4
FengA + FengB	FengA-B	Fengycin synthetases A and B	AKL76471 and AKL76470
FengB + FengC	FengB-C	Fengycin synthetases B and C	AKL76470 and AKL76469
FengC + FengD	FengC-D	Fengycin synthetases C and D	AKL76469 and AKL76468
FengD + FengE	FengD-E	Fengycin synthetases D and E	AKL76468 and AKL76467
PpsA + PpsB	PpsA-B	Plipastatin synthetases A and B	AGG61210 and AGG61209
PpsB + PpsC	PpsB-C	Plipastatin synthetases B and C	AGG61209 and AGG61208
PpcC + PpsD	PpsC-D	Plipastatin synthetases C and D	AGG61208 and AGG61207
PpsD + PpsE	PpsD-E	Plipastatin synthetases D and E	AGG61207 and AGG61206
FegR + FegS	FegR-S	Feglymycin synthetases R and S	ALK27915 and ALK27916
Tcp9 + Tcp10	Tcp9-10	Teicoplanin synthetases 1 and 2	CAG15009 and CAG15010

Amino acid sequences

Sequence sections differing from wild-type sequence are highlighted in yellow.

BacC4-C5[E-COM-C]-H_6

MGDKSASYETVEGEVLLTPIQQEYFSLNKTDRNHYNHAVMLYRKNGFDESIVKRVFKEIIKHHDALRTVFTEEDGKIIQYNRGPDKQLFDLFVYDVSSENDQPQKVYQLATELQQSIDIETG
PLVKLALFKTNNGDHLLIIIHHLVVDGISWRILFEDLAIGYSQLANGEKVEFYPKTASYQAYARHIAEYAKSVKLLSEKQYWLKAIAEGVEFLDMNENAGAFKVEDSRTFSTELEKEETKRLLR
ETNRAYHTEINDILITALLVAARDMNGQNQLRITLEGHGREQVADGIDISRTVGWFTSKYPVFIDLGQETDMSRTIKMVKEHLRNVPNKGIGYGILKYLTRDSEIAKGAASPILFNYLGQLDED
INSGEFSSSHLSPGEAAGKGITREHPLEINAVVFRGKLAIQTTYNTRAYSEDVVRAFAQNYKEALKAVIRHCAEREETEKTPSDYGDKGISLDQLEEIKLKYKGMEIEKIYPLANMQRGMLFH
ALEDKESQAYFEQMAINMKGLIDERLFAETFNDIMERHEILRASIEYEITDEPRNVIIKDRKINLDYHDLRKQSPAEREQVIQAYRKADREKGFRLNSEPLIRAALMRTEDDSYTFIWTNHHILL
DGWSRGIIMGELFHMYHMKEARQKHRLEEARPYSDYIGWLQQQDKEAAKAYWRNYLSGFTEKSPISVLAGSSGHAKYKRKEAVIEFPEQLTGRITELASRNNVTFHTVLQCIWGMLLARY
NQTDDVVFGTVISGRDAQVTGIEKMVGLFINTVPTRIRLDKSQSFKELIKSVQEQALEGRTYHDMNLSEVQSLSELKRELLDHILIFENYAVDQSAFETSGKRGAGFVFEEIHAEEQTNYGF
NIVAVPGERLVIKLTYDGNIYHDHIIAGIKGHLQQVMEQVVQHEDQSLNDITVLSGSRSHHHHHH

1) BacC4-C5[A-PCP-E-COM-C-A-PCP-TE]-H_6

MDSDEEMNALLDQNQGKQADYPQDQTVHQLFEQQADKTPEQTAVVYADEKLTYRELNERANQLARLLRDKGADADQPVAIMIEPSLEMIISMLAVLKAGAAYVPIEPEQLAKRTNEILSDS
RAAILLVKGSVKENVAFAGEIVNVADGLIDAKVASNLSASGSADQNAYIIYTSGSTGKPKGVFVRHGNVVNYTTWFMKEAGLTENDKAMLVSSYAFDLGYTSIFSALLSGSELHIARKECYT
NAHRALKYIKENGITYIKLTPSLFNIFVNDPGFSAEKPCATLRLVVLGGEMINTRDVETFYNQYPDHVVMNHYGPTETTIGSVFKVIDPEHLDSFKECPVIGTPIHNTNAYVLDENMKLLPEGV
YGELCIAGAGVTGGYVNRPDETKEKFIENPFAPHTKMYRTGDLARRLSDGNIELAGRIDTQVKVRGYRIEPEEIKNRLLAHDDIKEAFIAAREDHKGAKQLCAYFTADAELPFEDIRTYLMHE
LPEYMIPSSFVQIEKMPLSANGKIDTAALPEPQPGKETEYEPPRNETEEKLVQIWEEVLGIDKIGITHHFFAAGGDSIKALQMISRLSREGLSLEMKDLFANPQIKSLSRYVKAESDKSASYET
VEGEVLLTPIQQEYFSLNKTDRNHYNHAVMLYRKNGFDESIVKRVFKEIIKHHDALRTVFTEEDGKIIQYNRGPDKQLFDLFVYDVSSENDQPQKVYQLATELQQSIDIETGPLVKLALFKTN
NGDHLLIIIHHLVVDGISWRILFEDLAIGYSQLANGEKVEFYPKTASYQAYARHIAEYAKSVKLLSEKQYWLKAIAEGVEFLDMNENAGAFKVEDSRTFSTELEKEETKRLLRETNRAYHTEIN
DILITALLVAARDMNGQNQLRITLEGHGREQVADGIDISRTVGWFTSKYPVFIDLGQETDMSRTIKMVKEHLRNVPNKGIGYGILKYLTRDSEIAKGAASPILFNYLGQLDEDINSGEFSSSHL
SPGEAAGKGITREHPLEINAVVFRGKLAIQTTYNTRAYSEDVVRAFAQNYKEALKAVIRHCAEREETEKTPSDYGDKGISLDQLEEIKLKYKGMEIEKIYPLANMQRGMLFHALEDKESQAY
FEQMAINMKGLIDERLFAETFNDIMERHEILRASIEYEITDEPRNVIIKDRKINLDYHDLRKQSPAEREQVIQAYRKADREKGFRLNSEPLIRAALMRTEDDSYTFIWTNHHILLDGWSRGIIMG
ELFHMYHMKEARQKHRLEEARPYSDYIGWLQQQDKEAAKAYWRNYLSGFTEKSPISVLAGSSGHAKYKRKEAVIEFPEQLTGRITELASRNNVTFHTVLQCIWGMLLARYNQTDDVVFG
TVISGRDAQVTGIEKMVGLFINTVPTRIRLDKSQSFKELIKSVQEQALEGRTYHDMNLSEVQSLSELKRELLDHILIFENYAVDQSAFETSGKRGAGFVFEEIHAEEQTNYGFNIVAVPGERL
VIKLTYDGNIYHDHIIAGIKGHLQQVMEQVVQHEDQSLNDITVLSEAERNRLLYEWNDTKAEYPNQTIHRLFEEQAEKTPELAAVVSGNDKLTYRELNEKSNQLARYLRDKGVKADTIVAIMA
ERSPEMVVGIMGILKAGGAYLPIDPDYPEERIKYMLEDSGAAIILADHKQDLGTLHQEAVELTGDFSSYPADNLEPAGNADSLAYIIYTSGSTGKPKGVMIRQRGLVNYITWADRVYVQGEQ
LDFALYSSIAFDLTVTSIFTPLISGNRVIVYRHSEDGEPLIRKVFRDQKAGIVKLTPSHLSLVKDMDASGSSIKRLIVGGEDLKTELAKEITERFHHNIEIYNEYGPTETVVGCMIYQYDAGWDR
QVSVPIGKPASNVQLYILDERQEVQPVGIAGELYISGDGVAKGYLNKPELTSEKFLPNPFLPGERMYRTGDLAKMRPDGHIEYLGRIDHQVKIRGYRIELGEIEHQLLRHSDIKEAAVAAKTD
QNNDQVLCAYVVSERDITQKDIKTFLAKELPEYMVPSYLLKLDELPLTPNGKVDLKALPEPDRSAGALLEYEPPRHELEEKMAAIWEDILNIEQIGINANIFDIGANSLNVMSFVSRLYAELGF
RVPFKDIFSKPTIKELSDFLKHAQDLLKDYTDDCMQLTRAEEGGKNLFCFPPAASMGIAYMGLAKHLKQHSVYSFNFIPSANRIRKYADIIKNIQGEGPYTLIGYSSGGILAFDVAKELNRQG
YEVEDLIIIDSKYRTKAEKHQFTEEEYREEISKTFELEKYRDVEKLLSDYLVDLVMKSYVYIQNTVTTGAIDGHISYIKSSDNQRGENMMMWEKATSKTFTVVQGAGTHMQMISKSHPDILER
NARLIHDIINKTVKIGSRSHHHHHH

2) BacC4-C5[A-PCP-E-COM-C-A-PCP-TE(S6204A)]-H_6

MDSDEEMNALLDQNQGKQADYPQDQTVHQLFEQQADKTPEQTAVVYADEKLTYRELNERANQLARLLRDKGADADQPVAIMIEPSLEMIISMLAVLKAGAAYVPIEPEQLAKRTNEILSDS
RAAILLVKGSVKENVAFAGEIVNVADGLIDAKVASNLSASGSADQNAYIIYTSGSTGKPKGVFVRHGNVVNYTTWFMKEAGLTENDKAMLVSSYAFDLGYTSIFSALLSGSELHIARKECYT
NAHRALKYIKENGITYIKLTPSLFNIFVNDPGFSAEKPCATLRLVVLGGEMINTRDVETFYNQYPDHVVMNHYGPTETTIGSVFKVIDPEHLDSFKECPVIGTPIHNTNAYVLDENMKLLPEGV
YGELCIAGAGVTGGYVNRPDETKEKFIENPFAPHTKMYRTGDLARRLSDGNIELAGRIDTQVKVRGYRIEPEEIKNRLLAHDDIKEAFIAAREDHKGAKQLCAYFTADAELPFEDIRTYLMHE
LPEYMIPSSFVQIEKMPLSANGKIDTAALPEPQPGKETEYEPPRNETEEKLVQIWEEVLGIDKIGITHHFFAAGGDSIKALQMISRLSREGLSLEMKDLFANPQIKSLSRYVKAESDKSASYET
VEGEVLLTPIQQEYFSLNKTDRNHYNHAVMLYRKNGFDESIVKRVFKEIIKHHDALRTVFTEEDGKIIQYNRGPDKQLFDLFVYDVSSENDQPQKVYQLATELQQSIDIETGPLVKLALFKTN
NGDHLLIIIHHLVVDGISWRILFEDLAIGYSQLANGEKVEFYPKTASYQAYARHIAEYAKSVKLLSEKQYWLKAIAEGVEFLDMNENAGAFKVEDSRTFSTELEKEETKRLLRETNRAYHTEIN
DILITALLVAARDMNGQNQLRITLEGHGREQVADGIDISRTVGWFTSKYPVFIDLGQETDMSRTIKMVKEHLRNVPNKGIGYGILKYLTRDSEIAKGAASPILFNYLGQLDEDINSGEFSSSHL
SPGEAAGKGITREHPLEINAVVFRGKLAIQTTYNTRAYSEDVVRAFAQNYKEALKAVIRHCAEREETEKTPSDYGDKGISLDQLEEIKLKYKGMEIEKIYPLANMQRGMLFHALEDKESQAY
FEQMAINMKGLIDERLFAETFNDIMERHEILRASIEYEITDEPRNVIIKDRKINLDYHDLRKQSPAEREQVIQAYRKADREKGFRLNSEPLIRAALMRTEDDSYTFIWTNHHILLDGWSRGIIMG
ELFHMYHMKEARQKHRLEEARPYSDYIGWLQQQDKEAAKAYWRNYLSGFTEKSPISVLAGSSGHAKYKRKEAVIEFPEQLTGRITELASRNNVTFHTVLQCIWGMLLARYNQTDDVVFG
TVISGRDAQVTGIEKMVGLFINTVPTRIRLDKSQSFKELIKSVQEQALEGRTYHDMNLSEVQSLSELKRELLDHILIFENYAVDQSAFETSGKRGAGFVFEEIHAEEQTNYGFNIVAVPGERL
VIKLTYDGNIYHDHIIAGIKGHLQQVMEQVVQHEDQSLNDITVLSEAERNRLLYEWNDTKAEYPNQTIHRLFEEQAEKTPELAAVVSGNDKLTYRELNEKSNQLARYLRDKGVKADTIVAIMA
ERSPEMVVGIMGILKAGGAYLPIDPDYPEERIKYMLEDSGAAIILADHKQDLGTLHQEAVELTGDFSSYPADNLEPAGNADSLAYIIYTSGSTGKPKGVMIRQRGLVNYITWADRVYVQGEQ
LDFALYSSIAFDLTVTSIFTPLISGNRVIVYRHSEDGEPLIRKVFRDQKAGIVKLTPSHLSLVKDMDASGSSIKRLIVGGEDLKTELAKEITERFHHNIEIYNEYGPTETVVGCMIYQYDAGWDR
QVSVPIGKPASNVQLYILDERQEVQPVGIAGELYISGDGVAKGYLNKPELTSEKFLPNPFLPGERMYRTGDLAKMRPDGHIEYLGRIDHQVKIRGYRIELGEIEHQLLRHSDIKEAAVAAKTD
QNNDQVLCAYVVSERDITQKDIKTFLAKELPEYMVPSYLLKLDELPLTPNGKVDLKALPEPDRSAGALLEYEPPRHELEEKMAAIWEDILNIEQIGINANIFDIGANSLNVMSFVSRLYAELGF
RVPFKDIFSKPTIKELSDFLKHAQDLLKDYTDDCMQLTRAEEGGKNLFCFPPAASMGIAYMGLAKHLKQHSVYSFNFIPSANRIRKYADIIKNIQGEGPYTLIGYASGGILAFDVAKELNRQG
YEVEDLIIIDSKYRTKAEKHQFTEEEYREEISKTFELEKYRDVEKLLSDYLVDLVMKSYVYIQNTVTTGAIDGHISYIKSSDNQRGENMMMWEKATSKTFTVVQGAGTHMQMISKSHPDILER
NARLIHDIINKTVKIGSRSHHHHHH

3) BacC4-C5[A-PCP-E(H4755A)-COM-C-A-PCP-TE]-H_6

MDSDEEMNALLDQNQGKQADYPQDQTVHQLFEQQADKTPEQTAVVYADEKLTYRELNERANQLARLLRDKGADADQPVAIMIEPSLEMIISMLAVLKAGAAYVPIEPEQLAKRTNEILSDS
RAAILLVKGSVKENVAFAGEIVNVADGLIDAKVASNLSASGSADQNAYIIYTSGSTGKPKGVFVRHGNVVNYTTWFMKEAGLTENDKAMLVSSYAFDLGYTSIFSALLSGSELHIARKECYT
NAHRALKYIKENGITYIKLTPSLFNIFVNDPGFSAEKPCATLRLVVLGGEMINTRDVETFYNQYPDHVVMNHYGPTETTIGSVFKVIDPEHLDSFKECPVIGTPIHNTNAYVLDENMKLLPEGV
YGELCIAGAGVTGGYVNRPDETKEKFIENPFAPHTKMYRTGDLARRLSDGNIELAGRIDTQVKVRGYRIEPEEIKNRLLAHDDIKEAFIAAREDHKGAKQLCAYFTADAELPFEDIRTYLMHE
LPEYMIPSSFVQIEKMPLSANGKIDTAALPEPQPGKETEYEPPRNETEEKLVQIWEEVLGIDKIGITHHFFAAGGDSIKALQMISRLSREGLSLEMKDLFANPQIKSLSRYVKAESDKSASYET
VEGEVLLTPIQQEYFSLNKTDRNHYNHAVMLYRKNGFDESIVKRVFKEIIKHHDALRTVFTEEDGKIIQYNRGPDKQLFDLFVYDVSSENDQPQKVYQLATELQQSIDIETGPLVKLALFKTN
NGDHLLIIIHALVVDGISWRILFEDLAIGYSQLANGEKVEFYPKTASYQAYARHIAEYAKSVKLLSEKQYWLKAIAEGVEFLDMNENAGAFKVEDSRTFSTELEKEETKRLLRETNRAYHTEIN
DILITALLVAARDMNGQNQLRITLEGHGREQVADGIDISRTVGWFTSKYPVFIDLGQETDMSRTIKMVKEHLRNVPNKGIGYGILKYLTRDSEIAKGAASPILFNYLGQLDEDINSGEFSSSHL
SPGEAAGKGITREHPLEINAVVFRGKLAIQTTYNTRAYSEDVVRAFAQNYKEALKAVIRHCAEREETEKTPSDYGDKGISLDQLEEIKLKYKGMEIEKIYPLANMQRGMLFHALEDKESQAY
FEQMAINMKGLIDERLFAETFNDIMERHEILRASIEYEITDEPRNVIIKDRKINLDYHDLRKQSPAEREQVIQAYRKADREKGFRLNSEPLIRAALMRTEDDSYTFIWTNHHILLDGWSRGIIMG
ELFHMYHMKEARQKHRLEEARPYSDYIGWLQQQDKEAAKAYWRNYLSGFTEKSPISVLAGSSGHAKYKRKEAVIEFPEQLTGRITELASRNNVTFHTVLQCIWGMLLARYNQTDDVVFG
TVISGRDAQVTGIEKMVGLFINTVPTRIRLDKSQSFKELIKSVQEQALEGRTYHDMNLSEVQSLSELKRELLDHILIFENYAVDQSAFETSGKRGAGFVFEEIHAEEQTNYGFNIVAVPGERL
VIKLTYDGNIYHDHIIAGIKGHLQQVMEQVVQHEDQSLNDITVLSEAERNRLLYEWNDTKAEYPNQTIHRLFEEQAEKTPELAAVVSGNDKLTYRELNEKSNQLARYLRDKGVKADTIVAIMA
ERSPEMVVGIMGILKAGGAYLPIDPDYPEERIKYMLEDSGAAIILADHKQDLGTLHQEAVELTGDFSSYPADNLEPAGNADSLAYIIYTSGSTGKPKGVMIRQRGLVNYITWADRVYVQGEQ
LDFALYSSIAFDLTVTSIFTPLISGNRVIVYRHSEDGEPLIRKVFRDQKAGIVKLTPSHLSLVKDMDASGSSIKRLIVGGEDLKTELAKEITERFHHNIEIYNEYGPTETVVGCMIYQYDAGWDR
QVSVPIGKPASNVQLYILDERQEVQPVGIAGELYISGDGVAKGYLNKPELTSEKFLPNPFLPGERMYRTGDLAKMRPDGHIEYLGRIDHQVKIRGYRIELGEIEHQLLRHSDIKEAAVAAKTD
QNNDQVLCAYVVSERDITQKDIKTFLAKELPEYMVPSYLLKLDELPLTPNGKVDLKALPEPDRSAGALLEYEPPRHELEEKMAAIWEDILNIEQIGINANIFDIGANSLNVMSFVSRLYAELGF
RVPFKDIFSKPTIKELSDFLKHAQDLLKDYTDDCMQLTRAEEGGKNLFCFPPAASMGIAYMGLAKHLKQHSVYSFNFIPSANRIRKYADIIKNIQGEGPYTLIGYSSGGILAFDVAKELNRQG
YEVEDLIIIDSKYRTKAEKHQFTEEEYREEISKTFELEKYRDVEKLLSDYLVDLVMKSYVYIQNTVTTGAIDGHISYIKSSDNQRGENMMMWEKATSKTFTVVQGAGTHMQMISKSHPDILER
NARLIHDIINKTVKIGSRSHHHHHH

4) BacC4-C5[A-PCP-E(E4893A)-COM-C-A-PCP-TE]-H_6

MDSDEEMNALLDQNQGKQADYPQDQTVHQLFEQQADKTPEQTAVVYADEKLTYRELNERANQLARLLRDKGADADQPVAIMIEPSLEMIISMLAVLKAGAAYVPIEPEQLAKRTNEILSDS
RAAILLVKGSVKENVAFAGEIVNVADGLIDAKVASNLSASGSADQNAYIIYTSGSTGKPKGVFVRHGNVVNYTTWFMKEAGLTENDKAMLVSSYAFDLGYTSIFSALLSGSELHIARKECYT
NAHRALKYIKENGITYIKLTPSLFNIFVNDPGFSAEKPCATLRLVVLGGEMINTRDVETFYNQYPDHVVMNHYGPTETTIGSVFKVIDPEHLDSFKECPVIGTPIHNTNAYVLDENMKLLPEGV
YGELCIAGAGVTGGYVNRPDETKEKFIENPFAPHTKMYRTGDLARRLSDGNIELAGRIDTQVKVRGYRIEPEEIKNRLLAHDDIKEAFIAAREDHKGAKQLCAYFTADAELPFEDIRTYLMHE
LPEYMIPSSFVQIEKMPLSANGKIDTAALPEPQPGKETEYEPPRNETEEKLVQIWEEVLGIDKIGITHHFFAAGGDSIKALQMISRLSREGLSLEMKDLFANPQIKSLSRYVKAESDKSASYET
VEGEVLLTPIQQEYFSLNKTDRNHYNHAVMLYRKNGFDESIVKRVFKEIIKHHDALRTVFTEEDGKIIQYNRGPDKQLFDLFVYDVSSENDQPQKVYQLATELQQSIDIETGPLVKLALFKTN

NGDHLLIIIHHLVVDGISWRILFEDLAIGYSQLANGEKVEFYPKTASYQAYARHIAEYAKSVKLLSEKQYWLKAIAEGVEFLDMNENAGAFKVEDSRTFSTELEKEETKRLLRETNRAYHTEIN
DILITALLVAARDMNGQNQLRITLAGHGREQVADGIDISRTVGWFTSKYPVFIDLGQETDMSRTIKMVKEHLRNVPNKGIGYGILKYLTRDSEIAKGAASPILFNYLGQLDEDINSGEFSSSHL
SPGEAAGKGITREHPLEINAVVFRGKLAIQTTYNTRAYSEDVVRAFAQNYKEALKAVIRHCAEREETEKTPSDYGDKGISLDQLEEIKLKYKGMEIEKIYPLANMQRGMLFHALEDKESQAY
FEQMAINMKGLIDERLFAETFNDIMERHEILRASIEYEITDEPRNVIIKDRKINLDYHDLRKQSPAEREQVIQAYRKADREKGFRLNSEPLIRAALMRTEDDSYTFIWTNHHILLDGWSRGIIMG
ELFHMYHMKEARQKHRLEEARPYSDYIGWLQQQDKEAAKAYWRNYLSGFTEKSPISVLAGSSGHAKYKRKEAVIEFPEQLTGRITELASRNNVTFHTVLQCIWGMLLARYNQTDDVVFG
TVISGRDAQVTGIEKMVGLFINTVPTRIRLDKSQSFKELIKSVQEQALEGRTYHDMNLSEVQSLSELKRELLDHILIFENYAVDQSAFETSGKRGAGFVFEEIHAEEQTNYGFNIVAVPGERL
VIKLTYDGNIYHDHIIAGIKGHLQQVMEQVVQHEDQSLNDITVLSEAERNRLLYEWNDTKAEYPNQTIHRLFEEQAEKTPELAAVVSGNDKLTYRELNEKSNQLARYLRDKGVKADTIVAIMA
ERSPEMVVGIMGILKAGGAYLPIDPDYPEERIKYMLEDSGAAIILADHKQDLGTLHQEAVELTGDFSSYPADNLEPAGNADSLAYIIYTSGSTGKPKGVMIRQRGLVNYITWADRVYVQGEQ
LDFALYSSIAFDLTVTSIFTPLISGNRVIVYRHSEDGEPLIRKVFRDQKAGIVKLTPSHLSLVKDMDASGSSIKRLIVGGEDLKTELAKEITERFHHNIEIYNEYGPTETVVGCMIYQYDAGWDR
QVSVPIGKPASNVQLYILDERQEVQPVGIAGELYISGDGVAKGYLNKPELTSEKFLPNPFLPGERMYRTGDLAKMRPDGHIEYLGRIDHQVKIRGYRIELGEIEHQLLRHSDIKEAAVAAKTD
QNNDQVLCAYVVSERDITQKDIKTFLAKELPEYMVPSYLLKLDELPLTPNGKVDLKALPEPDRSAGALLEYEPPRHELEEKMAAIWEDILNIEQIGINANIFDIGANSLNVMSFVSRLYAELGF
RVPFKDIFSKPTIKELSDFLKHAQDLLKDYTDDCMQLTRAEEGGKNLFCFPPAASMGIAYMGLAKHLKQHSVYSFNFIPSANRIRKYADIIKNIQGEGPYTLIGYSSGGILAFDVAKELNRQG
YEVEDLIIIDSKYRTKAEKHQFTEEEYREEISKTFELEKYRDVEKLLSDYLVDLVMKSYVYIQNTVTTGAIDGHISYIKSSDNQRGENMMMWEKATSKTFTVVQGAGTHMQMISKSHPDILER
NARLIHDIINKTVKIGSRSHHHHHH

5) BacC4-C5[A-PCP-E-COM(I5079R)-C-A-PCP-TE]-H_6

MDSDEEMNALLDQNQGKQADYPQDQTVHQLFEQQADKTPEQTAVVYADEKLTYRELNERANQLARLLRDKGADADQPVAIMIEPSLEMIISMLAVLKAGAAYVPIEPEQLAKRTNEILSDS
RAAILLVKGSVKENVAFAGEIVNVADGLIDAKVASNLSASGSADQNAYIIYTSGSTGKPKGVFVRHGNVVNYTTWFMKEAGLTENDKAMLVSSYAFDLGYTSIFSALLSGSELHIARKECYT
NAHRALKYIKENGITYIKLTPSLFNIFVNDPGFSAEKPCATLRLVVLGGEMINTRDVETFYNQYPDHVVMNHYGPTETTIGSVFKVIDPEHLDSFKECPVIGTPIHNTNAYVLDENMKLLPEGV
YGELCIAGAGVTGGYVNRPDETKEKFIENPFAPHTKMYRTGDLARRLSDGNIELAGRIDTQVKVRGYRIEPEEIKNRLLAHDDIKEAFIAAREDHKGAKQLCAYFTADAELPFEDIRTYLMHE
LPEYMIPSSFVQIEKMPLSANGKIDTAALPEPQPGKETEYEPPRNETEEKLVQIWEEVLGIDKIGITHHFFAAGGDSIKALQMISRLSREGLSLEMKDLFANPQIKSLSRYVKAESDKSASYET
VEGEVLLTPIQQEYFSLNKTDRNHYNHAVMLYRKNGFDESIVKRVFKEIIKHHDALRTVFTEEDGKIIQYNRGPDKQLFDLFVYDVSSENDQPQKVYQLATELQQSIDIETGPLVKLALFKTN
NGDHLLIIIHHLVVDGISWRILFEDLAIGYSQLANGEKVEFYPKTASYQAYARHIAEYAKSVKLLSEKQYWLKAIAEGVEFLDMNENAGAFKVEDSRTFSTELEKEETKRLLRETNRAYHTEIN
DILITALLVAARDMNGQNQLRITLEGHGREQVADGIDISRTVGWFTSKYPVFIDLGQETDMSRTIKMVKEHLRNVPNKGIGYGILKYLTRDSEIAKGAASPILFNYLGQLDEDINSGEFSSSHL
SPGEAAGKGITREHPLEINAVVFRGKLAIQTTYNTRAYSEDVVRAFAQNYKEALKAVIRHCAEREETEKTPSDYGDKGISLDQLEERKLKYKGMEIEKIYPLANMQRGMLFHALEDKESQAY
FEQMAINMKGLIDERLFAETFNDIMERHEILRASIEYEITDEPRNVIIKDRKINLDYHDLRKQSPAEREQVIQAYRKADREKGFRLNSEPLIRAALMRTEDDSYTFIWTNHHILLDGWSRGIIMG
ELFHMYHMKEARQKHRLEEARPYSDYIGWLQQQDKEAAKAYWRNYLSGFTEKSPISVLAGSSGHAKYKRKEAVIEFPEQLTGRITELASRNNVTFHTVLQCIWGMLLARYNQTDDVVFG
TVISGRDAQVTGIEKMVGLFINTVPTRIRLDKSQSFKELIKSVQEQALEGRTYHDMNLSEVQSLSELKRELLDHILIFENYAVDQSAFETSGKRGAGFVFEEIHAEEQTNYGFNIVAVPGERL
VIKLTYDGNIYHDHIIAGIKGHLQQVMEQVVQHEDQSLNDITVLSEAERNRLLYEWNDTKAEYPNQTIHRLFEEQAEKTPELAAVVSGNDKLTYRELNEKSNQLARYLRDKGVKADTIVAIMA
ERSPEMVVGIMGILKAGGAYLPIDPDYPEERIKYMLEDSGAAIILADHKQDLGTLHQEAVELTGDFSSYPADNLEPAGNADSLAYIIYTSGSTGKPKGVMIRQRGLVNYITWADRVYVQGEQ
LDFALYSSIAFDLTVTSIFTPLISGNRVIVYRHSEDGEPLIRKVFRDQKAGIVKLTPSHLSLVKDMDASGSSIKRLIVGGEDLKTELAKEITERFHHNIEIYNEYGPTETVVGCMIYQYDAGWDR
QVSVPIGKPASNVQLYILDERQEVQPVGIAGELYISGDGVAKGYLNKPELTSEKFLPNPFLPGERMYRTGDLAKMRPDGHIEYLGRIDHQVKIRGYRIELGEIEHQLLRHSDIKEAAVAAKTD
QNNDQVLCAYVVSERDITQKDIKTFLAKELPEYMVPSYLLKLDELPLTPNGKVDLKALPEPDRSAGALLEYEPPRHELEEKMAAIWEDILNIEQIGINANIFDIGANSLNVMSFVSRLYAELGF
RVPFKDIFSKPTIKELSDFLKHAQDLLKDYTDDCMQLTRAEEGGKNLFCFPPAASMGIAYMGLAKHLKQHSVYSFNFIPSANRIRKYADIIKNIQGEGPYTLIGYSSGGILAFDVAKELNRQG
YEVEDLIIIDSKYRTKAEKHQFTEEEYREEISKTFELEKYRDVEKLLSDYLVDLVMKSYVYIQNTVTTGAIDGHISYIKSSDNQRGENMMMWEKATSKTFTVVQGAGTHMQMISKSHPDILER
NARLIHDIINKTVKIGSRSHHHHHH

6) BacC4-C5[A-PCP-E-COM(I5079A)-C-A-PCP-TE]-H_6

MDSDEEMNALLDQNQGKQADYPQDQTVHQLFEQQADKTPEQTAVVYADEKLTYRELNERANQLARLLRDKGADADQPVAIMIEPSLEMIISMLAVLKAGAAYVPIEPEQLAKRTNEILSDS
RAAILLVKGSVKENVAFAGEIVNVADGLIDAKVASNLSASGSADQNAYIIYTSGSTGKPKGVFVRHGNVVNYTTWFMKEAGLTENDKAMLVSSYAFDLGYTSIFSALLSGSELHIARKECYT
NAHRALKYIKENGITYIKLTPSLFNIFVNDPGFSAEKPCATLRLVVLGGEMINTRDVETFYNQYPDHVVMNHYGPTETTIGSVFKVIDPEHLDSFKECPVIGTPIHNTNAYVLDENMKLLPEGV
YGELCIAGAGVTGGYVNRPDETKEKFIENPFAPHTKMYRTGDLARRLSDGNIELAGRIDTQVKVRGYRIEPEEIKNRLLAHDDIKEAFIAAREDHKGAKQLCAYFTADAELPFEDIRTYLMHE
LPEYMIPSSFVQIEKMPLSANGKIDTAALPEPQPGKETEYEPPRNETEEKLVQIWEEVLGIDKIGITHHFFAAGGDSIKALQMISRLSREGLSLEMKDLFANPQIKSLSRYVKAESDKSASYET
VEGEVLLTPIQQEYFSLNKTDRNHYNHAVMLYRKNGFDESIVKRVFKEIIKHHDALRTVFTEEDGKIIQYNRGPDKQLFDLFVYDVSSENDQPQKVYQLATELQQSIDIETGPLVKLALFKTN
NGDHLLIIIHHLVVDGISWRILFEDLAIGYSQLANGEKVEFYPKTASYQAYARHIAEYAKSVKLLSEKQYWLKAIAEGVEFLDMNENAGAFKVEDSRTFSTELEKEETKRLLRETNRAYHTEIN
DILITALLVAARDMNGQNQLRITLEGHGREQVADGIDISRTVGWFTSKYPVFIDLGQETDMSRTIKMVKEHLRNVPNKGIGYGILKYLTRDSEIAKGAASPILFNYLGQLDEDINSGEFSSSHL
SPGEAAGKGITREHPLEINAVVFRGKLAIQTTYNTRAYSEDVVRAFAQNYKEALKAVIRHCAEREETEKTPSDYGDKGISLDQLEEAKLKYKGMEIEKIYPLANMQRGMLFHALEDKESQAY
FEQMAINMKGLIDERLFAETFNDIMERHEILRASIEYEITDEPRNVIIKDRKINLDYHDLRKQSPAEREQVIQAYRKADREKGFRLNSEPLIRAALMRTEDDSYTFIWTNHHILLDGWSRGIIMG
ELFHMYHMKEARQKHRLEEARPYSDYIGWLQQQDKEAAKAYWRNYLSGFTEKSPISVLAGSSGHAKYKRKEAVIEFPEQLTGRITELASRNNVTFHTVLQCIWGMLLARYNQTDDVVFG
TVISGRDAQVTGIEKMVGLFINTVPTRIRLDKSQSFKELIKSVQEQALEGRTYHDMNLSEVQSLSELKRELLDHILIFENYAVDQSAFETSGKRGAGFVFEEIHAEEQTNYGFNIVAVPGERL
VIKLTYDGNIYHDHIIAGIKGHLQQVMEQVVQHEDQSLNDITVLSEAERNRLLYEWNDTKAEYPNQTIHRLFEEQAEKTPELAAVVSGNDKLTYRELNEKSNQLARYLRDKGVKADTIVAIMA
ERSPEMVVGIMGILKAGGAYLPIDPDYPEERIKYMLEDSGAAIILADHKQDLGTLHQEAVELTGDFSSYPADNLEPAGNADSLAYIIYTSGSTGKPKGVMIRQRGLVNYITWADRVYVQGEQ
LDFALYSSIAFDLTVTSIFTPLISGNRVIVYRHSEDGEPLIRKVFRDQKAGIVKLTPSHLSLVKDMDASGSSIKRLIVGGEDLKTELAKEITERFHHNIEIYNEYGPTETVVGCMIYQYDAGWDR
QVSVPIGKPASNVQLYILDERQEVQPVGIAGELYISGDGVAKGYLNKPELTSEKFLPNPFLPGERMYRTGDLAKMRPDGHIEYLGRIDHQVKIRGYRIELGEIEHQLLRHSDIKEAAVAAKTD
QNNDQVLCAYVVSERDITQKDIKTFLAKELPEYMVPSYLLKLDELPLTPNGKVDLKALPEPDRSAGALLEYEPPRHELEEKMAAIWEDILNIEQIGINANIFDIGANSLNVMSFVSRLYAELGF
RVPFKDIFSKPTIKELSDFLKHAQDLLKDYTDDCMQLTRAEEGGKNLFCFPPAASMGIAYMGLAKHLKQHSVYSFNFIPSANRIRKYADIIKNIQGEGPYTLIGYSSGGILAFDVAKELNRQG
YEVEDLIIIDSKYRTKAEKHQFTEEEYREEISKTFELEKYRDVEKLLSDYLVDLVMKSYVYIQNTVTTGAIDGHISYIKSSDNQRGENMMMWEKATSKTFTVVQGAGTHMQMISKSHPDILER
NARLIHDIINKTVKIGSRSHHHHHH

7) BacC4-C5[A-PCP-E-COM(Δ5059-5069)-C-A-PCP-TE]-H_6

MDSDEEMNALLDQNQGKQADYPQDQTVHQLFEQQADKTPEQTAVVYADEKLTYRELNERANQLARLLRDKGADADQPVAIMIEPSLEMIISMLAVLKAGAAYVPIEPEQLAKRTNEILSDS
RAAILLVKGSVKENVAFAGEIVNVADGLIDAKVASNLSASGSADQNAYIIYTSGSTGKPKGVFVRHGNVVNYTTWFMKEAGLTENDKAMLVSSYAFDLGYTSIFSALLSGSELHIARKECYT
NAHRALKYIKENGITYIKLTPSLFNIFVNDPGFSAEKPCATLRLVVLGGEMINTRDVETFYNQYPDHVVMNHYGPTETTIGSVFKVIDPEHLDSFKECPVIGTPIHNTNAYVLDENMKLLPEGV
YGELCIAGAGVTGGYVNRPDETKEKFIENPFAPHTKMYRTGDLARRLSDGNIELAGRIDTQVKVRGYRIEPEEIKNRLLAHDDIKEAFIAAREDHKGAKQLCAYFTADAELPFEDIRTYLMHE
LPEYMIPSSFVQIEKMPLSANGKIDTAALPEPQPGKETEYEPPRNETEEKLVQIWEEVLGIDKIGITHHFFAAGGDSIKALQMISRLSREGLSLEMKDLFANPQIKSLSRYVKAESDKSASYET
VEGEVLLTPIQQEYFSLNKTDRNHYNHAVMLYRKNGFDESIVKRVFKEIIKHHDALRTVFTEEDGKIIQYNRGPDKQLFDLFVYDVSSENDQPQKVYQLATELQQSIDIETGPLVKLALFKTN
NGDHLLIIIHHLVVDGISWRILFEDLAIGYSQLANGEKVEFYPKTASYQAYARHIAEYAKSVKLLSEKQYWLKAIAEGVEFLDMNENAGAFKVEDSRTFSTELEKEETKRLLRETNRAYHTEIN
DILITALLVAARDMNGQNQLRITLEGHGREQVADGIDISRTVGWFTSKYPVFIDLGQETDMSRTIKMVKEHLRNVPNKGIGYGILKYLTRDSEIAKGAASPILFNYLGQLDEDINSGEFSSSHL
SPGEAAGKGITREHPLEINAVVFRGKLAIQTTYNTRAYSEDVVRAFAQNYKEALKAVIRHCAEREEGISLDQLEEIKLKYKGMEIEKIYPLANMQRGMLFHALEDKESQAYFEQMAINMKGLI
DERLFAETFNDIMERHEILRASIEYEITDEPRNVIIKDRKINLDYHDLRKQSPAEREQVIQAYRKADREKGFRLNSEPLIRAALMRTEDDSYTFIWTNHHILLDGWSRGIIMGELFHMYHMKEA
RQKHRLEEARPYSDYIGWLQQQDKEAAKAYWRNYLSGFTEKSPISVLAGSSGHAKYKRKEAVIEFPEQLTGRITELASRNNVTFHTVLQCIWGMLLARYNQTDDVVFGTVISGRDAQVTG
IEKMVGLFINTVPTRIRLDKSQSFKELIKSVQEQALEGRTYHDMNLSEVQSLSELKRELLDHILIFENYAVDQSAFETSGKRGAGFVFEEIHAEEQTNYGFNIVAVPGERLVIKLTYDGNIYHD
HIIAGIKGHLQQVMEQVVQHEDQSLNDITVLSEAERNRLLYEWNDTKAEYPNQTIHRLFEEQAEKTPELAAVVSGNDKLTYRELNEKSNQLARYLRDKGVKADTIVAIMAERSPEMVVGIM
GILKAGGAYLPIDPDYPEERIKYMLEDSGAAIILADHKQDLGTLHQEAVELTGDFSSYPADNLEPAGNADSLAYIIYTSGSTGKPKGVMIRQRGLVNYITWADRVYVQGEQLDFALYSSIAFD
LTVTSIFTPLISGNRVIVYRHSEDGEPLIRKVFRDQKAGIVKLTPSHLSLVKDMDASGSSIKRLIVGGEDLKTELAKEITERFHHNIEIYNEYGPTETVVGCMIYQYDAGWDRQVSVPIGKPAS
NVQLYILDERQEVQPVGIAGELYISGDGVAKGYLNKPELTSEKFLPNPFLPGERMYRTGDLAKMRPDGHIEYLGRIDHQVKIRGYRIELGEIEHQLLRHSDIKEAAVAAKTDQNNDQVLCAY
VVSERDITQKDIKTFLAKELPEYMVPSYLLKLDELPLTPNGKVDLKALPEPDRSAGALLEYEPPRHELEEKMAAIWEDILNIEQIGINANIFDIGANSLNVMSFVSRLYAELGFRVPFKDIFSKP
TIKELSDFLKHAQDLLKDYTDDCMQLTRAEEGGKNLFCFPPAASMGIAYMGLAKHLKQHSVYSFNFIPSANRIRKYADIIKNIQGEGPYTLIGYSSGGILAFDVAKELNRQGYEVEDLIIIDSKY
RTKAEKHQFTEEEYREEISKTFELEKYRDVEKLLSDYLVDLVMKSYVYIQNTVTTGAIDGHISYIKSSDNQRGENMMMWEKATSKTFTVVQGAGTHMQMISKSHPDILERNARLIHDIINKTV
KIGSRSHHHHHH

8) BacC4-C5[A-PCP-E-COM(Δ5062-5084)-C-A-PCP-TE]-H_6

MDSDEEMNALLDQNQGKQADYPQDQTVHQLFEQQADKTPEQTAVVYADEKLTYRELNERANQLARLLRDKGADADQPVAIMIEPSLEMIISMLAVLKAGAAYVPIEPEQLAKRTNEILSDS
RAAILLVKGSVKENVAFAGEIVNVADGLIDAKVASNLSASGSADQNAYIIYTSGSTGKPKGVFVRHGNVVNYTTWFMKEAGLTENDKAMLVSSYAFDLGYTSIFSALLSGSELHIARKECYT
NAHRALKYIKENGITYIKLTPSLFNIFVNDPGFSAEKPCATLRLVVLGGEMINTRDVETFYNQYPDHVVMNHYGPTETTIGSVFKVIDPEHLDSFKECPVIGTPIHNTNAYVLDENMKLLPEGV
YGELCIAGAGVTGGYVNRPDETKEKFIENPFAPHTKMYRTGDLARRLSDGNIELAGRIDTQVKVRGYRIEPEEIKNRLLAHDDIKEAFIAAREDHKGAKQLCAYFTADAELPFEDIRTYLMHE
LPEYMIPSSFVQIEKMPLSANGKIDTAALPEPQPGKETEYEPPRNETEEKLVQIWEEVLGIDKIGITHHFFAAGGDSIKALQMISRLSREGLSLEMKDLFANPQIKSLSRYVKAESDKSASYET
VEGEVLLTPIQQEYFSLNKTDRNHYNHAVMLYRKNGFDESIVKRVFKEIIKHHDALRTVFTEEDGKIIQYNRGPDKQLFDLFVYDVSSENDQPQKVYQLATELQQSIDIETGPLVKLALFKTN
NGDHLLIIIHHLVVDGISWRILFEDLAIGYSQLANGEKVEFYPKTASYQAYARHIAEYAKSVKLLSEKQYWLKAIAEGVEFLDMNENAGAFKVEDSRTFSTELEKEETKRLLRETNRAYHTEIN
DILITALLVAARDMNGQNQLRITLEGHGREQVADGIDISRTVGWFTSKYPVFIDLGQETDMSRTIKMVKEHLRNVPNKGIGYGILKYLTRDSEIAKGAASPILFNYLGQLDEDINSGEFSSSHL
SPGEAAGKGITREHPLEINAVVFRGKLAIQTTYNTRAYSEDVVRAFAQNYKEALKAVIRHCAEREETEKGMEIEKIYPLANMQRGMLFHALEDKESQAYFEQMAINMKGLIDERLFAETFND

IMERHEILRASIEYEITDEPRNVIIKDRKINLDYHDLRKQSPAEREQVIQAYRKADREKGFRLNSEPLIRAALMRTEDDSYTFIWTNHHILLDGWSRGIIMGELFHMYHMKEARQKHRLEEARP
YSDYIGWLQQQDKEAAKAYWRNYLSGFTEKSPISVLAGSSGHAKYKRKEAVIEFPEQLTGRITELASRNNVTFHTVLQCIWGMLLARYNQTDDVVFGTVISGRDAQVTGIEKMVGLFINTV
PTRIRLDKSQSFKELIKSVQEQALEGRTYHDMNLSEVQSLSELKRELLDHILIFENYAVDQSAFETSGKRGAGFVFEEIHAEEQTNYGFNIVAVPGERLVIKLTYDGNIYHDHIIAGIKGHLQQ
VMEQVVQHEDQSLNDITVLSEAERNRLLYEWNDTKAEYPNQTIHRLFEEQAEKTPELAAVVSGNDKLTYRELNEKSNQLARYLRDKGVKADTIVAIMAERSPEMVVGIMGILKAGGAYLPI
DPDYPEERIKYMLEDSGAAIILADHKQDLGTLHQEAVELTGDFSSYPADNLEPAGNADSLAYIIYTSGSTGKPKGVMIRQRGLVNYITWADRVYVQGEQLDFALYSSIAFDLTVTSIFTPLISG
NRVIVYRHSEDGEPLIRKVFRDQKAGIVKLTPSHLSLVKDMDASGSSIKRLIVGGEDLKTELAKEITERFHHNIEIYNEYGPTETVVGCMIYQYDAGWDRQVSVPIGKPASNVQLYILDERQE
VQPVGIAGELYISGDGVAKGYLNKPELTSEKFLPNPFLPGERMYRTGDLAKMRPDGHIEYLGRIDHQVKIRGYRIELGEIEHQLLRHSDIKEAAVAAKTDQNNDQVLCAYVVSERDITQKDIK
TFLAKELPEYMVPSYLLKLDELPLTPNGKVDLKALPEPDRSAGALLEYEPPRHELEEKMAAIWEDILNIEQIGINANIFDIGANSLNVMSFVSRLYAELGFRVPFKDIFSKPTIKELSDFLKHAQ
DLLKDYTDDCMQLTRAEEGGKNLFCFPPAASMGIAYMGLAKHLKQHSVYSFNFIPSANRIRKYADIIKNIQGEGPYTLIGYSSGGILAFDVAKELNRQGYEVEDLIIIDSKYRTKAEKHQFTEE
EYREEISKTFELEKYRDVEKLLSDYLVDLVMKSYVYIQNTVTTGAIDGHISYIKSSDNQRGENMMMWEKATSKTFTVVQGAGTHMQMISKSHPDILERNARLIHDIINKTVKIGSRSHHHHHH

9) BacC4-C5[A-PCP-E-COM(Ins5062$(GGS)_3$)-C-A-PCP-TE]-H_6

MDSDEEMNALLDQNQGKQADYPQDQTVHQLFEQQADKTPEQTAVVYADEKLTYRELNERANQLARLLRDKGADADQPVAIMIEPSLEMIISMLAVLKAGAAYVPIEPEQLAKRTNEILSDS
RAAILLVKGSVKENVAFAGEIVNVADGLIDAKVASNLSASGSADQNAYIIYTSGSTGKPKGVFVRHGNVVNYTTWFMKEAGLTENDKAMLVSSYAFDLGYTSIFSALLSGSELHIARKECYT
NAHRALKYIKENGITYIKLTPSLFNIFVNDPGFSAEKPCATLRLVVLGGEMINTRDVETFYNQYPDHVVMNHYGPTETTIGSVFKVIDPEHLDSFKECPVIGTPIHNTNAYVLDENMKLLPEGV
YGELCIAGAGVTGGYVNRPDETKEKFIENPFAPHTKMYRTGDLARRLSDGNIELAGRIDTQVKVRGYRIEPEEIKNRLLAHDDIKEAFIAAREDHKGAKQLCAYFTADAELPFEDIRTYLMHE
LPEYMIPSSFVQIEKMPLSANGKIDTAALPEPQPGKETEYEPPRNETEEKLVQIWEEVLGIDKIGITHHFFAAGGDSIKALQMISRLSREGLSLEMKDLFANPQIKSLSRYVKAESDKSASYET
VEGEVLLTPIQQEYFSLNKTDRNHYNHAVMLYRKNGFDESIVKRVFKEIIKHHDALRTVFTEEDGKIIQYNRGPDKQLFDLFVYDVSSENDQPQKVYQLATELQQSIDIETGPLVKLALFKTN
NGDHLLIIIHHLVVDGISWRILFEDLAIGYSQLANGEKVEFYPKTASYQAYARHIAEYAKSVKLLSEKQYWLKAIAEGVEFLDMNENAGAFKVEDSRTFSTELEKEETKRLLRETNRAYHTEIN
DILITALLVAARDMNGQNQLRITLEGHGREQVADGIDISRTVGWFTSKYPVFIDLGQETDMSRTIKMVKEHLRNVPNKGIGYGILKYLTRDSEIAKGAASPILFNYLGQLDEDINSGEFSSSHL
SPGEAAGKGITREHPLEINAVVFRGKLAIQTTYNTRAYSEDVVRAFAQNYKEALKAVIRHCAEREETEKggsggsggsTPSDYGDKGISLDQLEEIKLKYKGMEIEKIYPLANMQRGMLFHALE
DKESQAYFEQMAINMKGLIDERLFAETFNDIMERHEILRASIEYEITDEPRNVIIKDRKINLDYHDLRKQSPAEREQVIQAYRKADREKGFRLNSEPLIRAALMRTEDDSYTFIWTNHHILLDG
WSRGIIMGELFHMYHMKEARQKHRLEEARPYSDYIGWLQQQDKEAAKAYWRNYLSGFTEKSPISVLAGSSGHAKYKRKEAVIEFPEQLTGRITELASRNNVTFHTVLQCIWGMLLARYNQ
TDDVVFGTVISGRDAQVTGIEKMVGLFINTVPTRIRLDKSQSFKELIKSVQEQALEGRTYHDMNLSEVQSLSELKRELLDHILIFENYAVDQSAFETSGKRGAGFVFEEIHAEEQTNYGFNIV
AVPGERLVIKLTYDGNIYHDHIIAGIKGHLQQVMEQVVQHEDQSLNDITVLSEAERNRLLYEWNDTKAEYPNQTIHRLFEEQAEKTPELAAVVSGNDKLTYRELNEKSNQLARYLRDKGVKA
DTIVAIMAERSPEMVVGIMGILKAGGAYLPIDPDYPEERIKYMLEDSGAAIILADHKQDLGTLHQEAVELTGDFSSYPADNLEPAGNADSLAYIIYTSGSTGKPKGVMIRQRGLVNYITWADR
VYVQGEQLDFALYSSIAFDLTVTSIFTPLISGNRVIVYRHSEDGEPLIRKVFRDQKAGIVKLTPSHLSLVKDMDASGSSIKRLIVGGEDLKTELAKEITERFHHNIEIYNEYGPTETVVGCMIYQ
YDAGWDRQVSVPIGKPASNVQLYILDERQEVQPVGIAGELYISGDGVAKGYLNKPELTSEKFLPNPFLPGERMYRTGDLAKMRPDGHIEYLGRIDHQVKIRGYRIELGEIEHQLLRHSDIKE
AAVAAKTDQNNDQVLCAYVVSERDITQKDIKTFLAKELPEYMVPSYLLKLDELPLTPNGKVDLKALPEPDRSAGALLEYEPPRHELEEKMAAIWEDILNIEQIGINANIFDIGANSLNVMSFVS
RLYAELGFRVPFKDIFSKPTIKELSDFLKHAQDLLKDYTDDCMQLTRAEEGGKNLFCFPPAASMGIAYMGLAKHLKQHSVYSFNFIPSANRIRKYADIIKNIQGEGPYTLIGYSSGGILAFDVA
KELNRQGYEVEDLIIIDSKYRTKAEKHQFTEEEYREEISKTFELEKYRDVEKLLSDYLVDLVMKSYVYIQNTVTTGAIDGHISYIKSSDNQRGENMMMWEKATSKTFTVVQGAGTHMQMISK
SHPDILERNARLIHDIINKTVKIGSRSHHHHHH

10) BacC4-C5[A-PCP-E-COM(Δ5059-5061)-C-A-PCP-TE]-H_6

MDSDEEMNALLDQNQGKQADYPQDQTVHQLFEQQADKTPEQTAVVYADEKLTYRELNERANQLARLLRDKGADADQPVAIMIEPSLEMIISMLAVLKAGAAYVPIEPEQLAKRTNEILSDS
RAAILLVKGSVKENVAFAGEIVNVADGLIDAKVASNLSASGSADQNAYIIYTSGSTGKPKGVFVRHGNVVNYTTWFMKEAGLTENDKAMLVSSYAFDLGYTSIFSALLSGSELHIARKECYT
NAHRALKYIKENGITYIKLTPSLFNIFVNDPGFSAEKPCATLRLVVLGGEMINTRDVETFYNQYPDHVVMNHYGPTETTIGSVFKVIDPEHLDSFKECPVIGTPIHNTNAYVLDENMKLLPEGV
YGELCIAGAGVTGGYVNRPDETKEKFIENPFAPHTKMYRTGDLARRLSDGNIELAGRIDTQVKVRGYRIEPEEIKNRLLAHDDIKEAFIAAREDHKGAKQLCAYFTADAELPFEDIRTYLMHE
LPEYMIPSSFVQIEKMPLSANGKIDTAALPEPQPGKETEYEPPRNETEEKLVQIWEEVLGIDKIGITHHFFAAGGDSIKALQMISRLSREGLSLEMKDLFANPQIKSLSRYVKAESDKSASYET
VEGEVLLTPIQQEYFSLNKTDRNHYNHAVMLYRKNGFDESIVKRVFKEIIKHHDALRTVFTEEDGKIIQYNRGPDKQLFDLFVYDVSSENDQPQKVYQLATELQQSIDIETGPLVKLALFKTN
NGDHLLIIIHHLVVDGISWRILFEDLAIGYSQLANGEKVEFYPKTASYQAYARHIAEYAKSVKLLSEKQYWLKAIAEGVEFLDMNENAGAFKVEDSRTFSTELEKEETKRLLRETNRAYHTEIN
DILITALLVAARDMNGQNQLRITLEGHGREQVADGIDISRTVGWFTSKYPVFIDLGQETDMSRTIKMVKEHLRNVPNKGIGYGILKYLTRDSEIAKGAASPILFNYLGQLDEDINSGEFSSSHL
SPGEAAGKGITREHPLEINAVVFRGKLAIQTTYNTRAYSEDVVRAFAQNYKEALKAVIRHCAEREETPSDYGDKGISLDQLEEIKLKYKGMEIEKIYPLANMQRGMLFHALEDKESQAYFEQ
MAINMKGLIDERLFAETFNDIMERHEILRASIEYEITDEPRNVIIKDRKINLDYHDLRKQSPAEREQVIQAYRKADREKGFRLNSEPLIRAALMRTEDDSYTFIWTNHHILLDGWSRGIIMGELF
HMYHMKEARQKHRLEEARPYSDYIGWLQQQDKEAAKAYWRNYLSGFTEKSPISVLAGSSGHAKYKRKEAVIEFPEQLTGRITELASRNNVTFHTVLQCIWGMLLARYNQTDDVVFGTVIS
GRDAQVTGIEKMVGLFINTVPTRIRLDKSQSFKELIKSVQEQALEGRTYHDMNLSEVQSLSELKRELLDHILIFENYAVDQSAFETSGKRGAGFVFEEIHAEEQTNYGFNIVAVPGERLVIKLT
YDGNIYHDHIIAGIKGHLQQVMEQVVQHEDQSLNDITVLSEAERNRLLYEWNDTKAEYPNQTIHRLFEEQAEKTPELAAVVSGNDKLTYRELNEKSNQLARYLRDKGVKADTIVAIMAERSP
EMVVGIMGILKAGGAYLPIDPDYPEERIKYMLEDSGAAIILADHKQDLGTLHQEAVELTGDFSSYPADNLEPAGNADSLAYIIYTSGSTGKPKGVMIRQRGLVNYITWADRVYVQGEQLDFA
LYSSIAFDLTVTSIFTPLISGNRVIVYRHSEDGEPLIRKVFRDQKAGIVKLTPSHLSLVKDMDASGSSIKRLIVGGEDLKTELAKEITERFHHNIEIYNEYGPTETVVGCMIYQYDAGWDRQVSV
PIGKPASNVQLYILDERQEVQPVGIAGELYISGDGVAKGYLNKPELTSEKFLPNPFLPGERMYRTGDLAKMRPDGHIEYLGRIDHQVKIRGYRIELGEIEHQLLRHSDIKEAAVAAKTDQNND
QVLCAYVVSERDITQKDIKTFLAKELPEYMVPSYLLKLDELPLTPNGKVDLKALPEPDRSAGALLEYEPPRHELEEKMAAIWEDILNIEQIGINANIFDIGANSLNVMSFVSRLYAELGFRVPFK
DIFSKPTIKELSDFLKHAQDLLKDYTDDCMQLTRAEEGGKNLFCFPPAASMGIAYMGLAKHLKQHSVYSFNFIPSANRIRKYADIIKNIQGEGPYTLIGYSSGGILAFDVAKELNRQGYEVEDL
IIIDSKYRTKAEKHQFTEEEYREEISKTFELEKYRDVEKLLSDYLVDLVMKSYVYIQNTVTTGAIDGHISYIKSSDNQRGENMMMWEKATSKTFTVVQGAGTHMQMISKSHPDILERNARLIH
DIINKTVKIGSRSHHHHHH

11) BacC4-C5[A-PCP-E-COM(Ins5062G)-C-A-PCP-TE]-H_6

MDSDEEMNALLDQNQGKQADYPQDQTVHQLFEQQADKTPEQTAVVYADEKLTYRELNERANQLARLLRDKGADADQPVAIMIEPSLEMIISMLAVLKAGAAYVPIEPEQLAKRTNEILSDS
RAAILLVKGSVKENVAFAGEIVNVADGLIDAKVASNLSASGSADQNAYIIYTSGSTGKPKGVFVRHGNVVNYTTWFMKEAGLTENDKAMLVSSYAFDLGYTSIFSALLSGSELHIARKECYT
NAHRALKYIKENGITYIKLTPSLFNIFVNDPGFSAEKPCATLRLVVLGGEMINTRDVETFYNQYPDHVVMNHYGPTETTIGSVFKVIDPEHLDSFKECPVIGTPIHNTNAYVLDENMKLLPEGV
YGELCIAGAGVTGGYVNRPDETKEKFIENPFAPHTKMYRTGDLARRLSDGNIELAGRIDTQVKVRGYRIEPEEIKNRLLAHDDIKEAFIAAREDHKGAKQLCAYFTADAELPFEDIRTYLMHE
LPEYMIPSSFVQIEKMPLSANGKIDTAALPEPQPGKETEYEPPRNETEEKLVQIWEEVLGIDKIGITHHFFAAGGDSIKALQMISRLSREGLSLEMKDLFANPQIKSLSRYVKAESDKSASYET
VEGEVLLTPIQQEYFSLNKTDRNHYNHAVMLYRKNGFDESIVKRVFKEIIKHHDALRTVFTEEDGKIIQYNRGPDKQLFDLFVYDVSSENDQPQKVYQLATELQQSIDIETGPLVKLALFKTN
NGDHLLIIIHHLVVDGISWRILFEDLAIGYSQLANGEKVEFYPKTASYQAYARHIAEYAKSVKLLSEKQYWLKAIAEGVEFLDMNENAGAFKVEDSRTFSTELEKEETKRLLRETNRAYHTEIN
DILITALLVAARDMNGQNQLRITLEGHGREQVADGIDISRTVGWFTSKYPVFIDLGQETDMSRTIKMVKEHLRNVPNKGIGYGILKYLTRDSEIAKGAASPILFNYLGQLDEDINSGEFSSSHL
SPGEAAGKGITREHPLEINAVVFRGKLAIQTTYNTRAYSEDVVRAFAQNYKEALKAVIRHCAEREETEKgTPSDYGDKGISLDQLEEIKLKYKGMEIEKIYPLANMQRGMLFHALEDKESQA
YFEQMAINMKGLIDERLFAETFNDIMERHEILRASIEYEITDEPRNVIIKDRKINLDYHDLRKQSPAEREQVIQAYRKADREKGFRLNSEPLIRAALMRTEDDSYTFIWTNHHILLDGWSRGIIM
GELFHMYHMKEARQKHRLEEARPYSDYIGWLQQQDKEAAKAYWRNYLSGFTEKSSISVLAGSSGHAKYKRKEAVIEFPEQLTGRITELASRNNVTFHTVLQCIWGMLLARYNQTDDVVF
GTVISGRDAQVTGIEKMVGLFINTVPTRIRLDKSQSFKELIKSVQEQALEGRTYHDMNLSEVQSLSELKRELLDHILIFENYAVDQSAFETSGKRGAGFVFEEIHAEEQTNYGFNIVAVPGER
LVIKLTYDGNIYHDHIIAGIKGHLQQVMEQVVQHEDQSLNDITVLSEAERNRLLYEWNDTKAEYPNQTIHRLFEEQAEKTPELAAVVSGNDKLTYRELNEKSNQLARYLRDKGVKADTIVAIM
AERSPEMVVGIMGILKAGGAYLPIDPDYPEERIKYMLEDSGAAIIPADHKQDLGTLHQEAVELTGDFSSYPADNLEPAGNADSLAYIIYTSGSTGKPKGVMIRQRGLVNYITWADRVYVQGE
QLDFALYSSIAFDLTVTSIFTPLISGNRVIVYRHSEDGEPLIRKVFRDQKAGIVKLTPSHLSLVKDMDASGSSIKRLIVGGEDLKTELAKEITERFHHNIEIYNEYGPTETVVGCMIYQYDAGWD
RQVSVPIGKPASNVQLYILDERQEVQPVGIAGELYISGDGVAKGYLNKPELTSEKFLPNPFLPGERMYRTGDLAKMRPDGHIEYLGRIDHQVKIRGYRIELGEIEHQLLRHSDIKEAAVAAKT
DQNNDQVLCAYVVSERDITQKDIKTFLAKELPEYMVPSYLLKLDELPLTPNGKVDLKALPEPDRSAGALLEYEPPRHELEEKMAAIWEDILNIEQIGINANIFDIGANSLNVMSFVSRLYAELG
FRVPFKDIFSKPTIKELSDFLKHAQDLLKDYTDDCMQLTRAEEGGKNLFCFPPAASMGIAYMGLAKHLKQHSVYSFNFIPSANRIRKYADIIKNIQGEGPYTLIGYSSGGILAFDVAKELNRQG
YEVEDLIIIDSKYRTKAEKHQFTEEEYREEISKTFELEKYRDVEKLLSDYLVDLVMKSYVYIQNTVTTGAIDGHISYIKSSDNQRGENMMMWEKATSKTFTVVQGAGTHMQMISKSHPDILER
NARLIHDIINKTVKIGSRSHHHHHH

12) BacC4-C5[A-PCP-E-COM(Δ5059)-C-A-PCP-TE]-H_6

MDSDEEMNALLDQNQGKQADYPQDQTVHQLFEQQADKTPEQTAVVYADEKLTYRELNERANQLARLLRDKGADADQPVAIMIEPSLEMIISMLAVLKAGAAYVPIEPEQLAKRTNEILSDS
RAAILLVKGSVKENVAFAGEIVNVADGLIDAKVASNLSASGSADQNAYIIYTSGSTGKPKGVFVRHGNVVNYTTWFMKEAGLTENDKAMLVSSYAFDLGYTSIFSALLSGSELHIARKECYT
NAHRALKYIKENGITYIKLTPSLFNIFVNDPGFSAEKPCATLRLVVLGGEMINTRDVETFYNQYPDHVVMNHYGPTETTIGSVFKVIDPEHLDSFKECPVIGTPIHNTNAYVLDENMKLLPEGV
YGELCIAGAGVTGGYVNRPDETKEKFIENPFAPHTKMYRTGDLARRLSDGNIELAGRIDTQVKVRGYRIEPEEIKNRLLAHDDIKEAFIAAREDHKGAKQLCAYFTADAELPFEDIRTYLMHE
LPEYMIPSSFVQIEKMPLSANGKIDTAALPEPQPGKETEYEPPRNETEEKLVQIWEEVLGIDKIGITHHFFAAGGDSIKALQMISRLSREGLSLEMKDLFANPQIKSLSRYVKAESDKSASYET
VEGEVLLTPIQQEYFSLNKTDRNHYNHAVMLYRKNGFDESIVKRVFKEIIKHHDALRTVFTEEDGKIIQYNRGPDKQLFDLFVYDVSSENDQPQKVYQLATELQQSIDIETGPLVKLALFKTN
NGDHLLIIIHHLVVDGISWRILFEDLAIGYSQLANGEKVEFYPKTASYQAYARHIAEYAKSVKLLSEKQYWLKAIAEGVEFLDMNENAGAFKVEDSRTFSTELEKEETKRLLRETNRAYHTEIN
DILITALLVAARDMNGQNQLRITLEGHGREQVADGIDISRTVGWFTSKYPVFIDLGQETDMSRTIKMVKEHLRNVPNKGIGYGILKYLTRDSEIAKGAASPILFNYLGQLDEDINSGEFSSSHL
SPGEAAGKGITREHPLEINAVVFRGKLAIQTTYNTRAYSEDVVRAFAQNYKEALKAVIRHCAEREEEKTPSDYGDKGISLDQLEEIKLKYKGMEIEKIYPLANMQRGMLFHALEDKESQAYF
EQMAINMKGLIDERLFAETFNDIMERHEILRASIEYEITDEPRNVIIKDRKINLDYHDLRKQSPAEREQVIQAYRKADREKGFRLNSEPLIRAALMRTEDDSYTFIWTNHHILLDGWSRGIIMGE
LFHMYHMKEARQKHRLEEARPYSDYIGWLQQQDKEAAKAYWRNYLSGFTEKSPISVLAGSSGHAKYKRKEAVIEFPEQLTGRITELASRNNVTFHTVLQCIWGMLLARYNQTDDVVFGT
VISGRDAQVTGIEKMVGLFINTVPTRIRLDKSQSFKELIKSVQEQALEGRTYHDMNLSEVQSLSELKRELLDHILIFENYAVDQSAFETSGKRGAGFVFEEIHAEEQTNYGFNIVAVPGERLVI
KLTYDGNIYHDHIIAGIKGHLQQVMEQVVQHEDQSLNDITVLSEAERNRLLYEWNDTKAEYPNQTIHRLFEEQAEKTPELAAVVSGNDKLTYRELNEKSNQLARYLRDKGVKADTIVAIMAE

RSPEMVVGIMGILKAGGAYLPIDPDYPEERIKYMLEDSGAAIILADHKQDLGTLHQEAVELTGDFSSYPADNLEPAGNADSLAYIIYTSGSTGKPKGVMIRQRGLVNYITWADRVYVQGEQL
DFALYSSIAFDLTVTSIFTPLISGNRVIVYRHSEDGEPLIRKVFRDQKAGIVKLTPSHLSLVKDMDASGSSIKRLIVGGEDLKTELAKEITERFHHNIEIYNEYGPTETVVGCMIYQYDAGWDRQ
VSVPIGKPASNVQLYILDERQEVQPVGIAGELYISGDGVAKGYLNKPELTSEKFLPNPFLPGERMYRTGDLAKMRPDGHIEYLGRIDHQVKIRGYRIELGEIEHQLLRHSDIKEAAVAAKTDQ
NNDQVLCAYVVSERDITQKDIKTFLAKELPEYMVPSYLLKLDELPLTPNGKVDLKALPEPDRSAGALLEYEPPRHELEEKMAAIWEDILNIEQIGINANIFDIGANSLNVMSFVSRLYAELGFR
VPFKDIFSKPTIKELSDFLKHAQDLLKDYTDDCMQLTRAEEGGKNLFCFPPAASMGIAYMGLAKHLKQHSVYSFNFIPSANRIRKYADIIKNIQGEGPYTLIGYSSGGILAFDVAKELNRQGYE
VEDLIIIDSKYRTKAEKHQFTEEEYREEISKTFELEKYRDVEKLLSDYLVDLVMKSYVYIQNTVTTGAIDGHISYIKSSDNQRGENMMMWEKATSKTFTVVQGAGTHMQMISKSHPDILERNA
RLIHDIINKTVKIGSRSHHHHHH

13) BacC4-C5[A-PCP-E-COM(Ins5068G)-C-A-PCP-TE]-H$_6$

MDSDEEMNALLDQNQGKQADYPQDQTVHQLFEQQADKTPEQTAVVYADEKLTYRELNERANQLARLLRDKGADADQPVAIMIEPSLEMIISMLAVLKAGAAYVPIEPEQLAKRTNEILSDS
RAAILLVKGSVKENVAFAGEIVNVADGLIDAKVASNLSASGSADQNAYIIYTSGSTGKPKGVFVRHGNVVNYTTWFMKEAGLTENDKAMLVSSYAFDLGYTSIFSALLSGSELHIARKECYT
NAHRALKYIKENGITYIKLTPSLFNIFVNDPGFSAEKPCATLRLVVLGGEMINTRDVETFYNQYPDHVVMNHYGPTETTIGSVFKVIDPEHLDSFKECPVIGTPIHNTNAYVLDENMKLLPEGV
YGELCIAGAGVTGGYVNRPDETKEKFIENPFAPHTKMYRTGDLARRLSDGNIELAGRIDTQVKVRGYRIEPEEIKNRLLAHDDIKEAFIAAREDHKGAKQLCAYFTADAELPFEDIRTYLMHE
LPEYMIPSSFVQIEKMPLSANGKIDTAALPEPQPGKETEYEPPRNETEEKLVQIWEEVLGIDKIGITHHFFAAGGDSIKALQMISRLSREGLSLEMKDLFANPQIKSLSRYVKAESDKSASYET
VEGEVLLTPIQQEYFSLNKTDRNHYNHAVMLYRKNGFDESIVKRVFKEIIKHHDALRTVFTEEDGKIIQYNRGPDKQLFDLFVYDVSSENDQPQKVYQLATELQQSIDIETGPLVKLALFKTN
NGDHLLIIIHHLVVDGISWRILFEDLAIGYSQLANGEKVEFYPKTASYQAYARHIAEYAKSVKLLSEKQYWLKAIAEGVEFLDMNENAGAFKVEDSRTFSTELEKEETKRLLRETNRAYHTEIN
DILITALLVAARDMNGQNQLRITLEGHGREQVADGIDISRTVGWFTSKYPVFIDLGQETDMSRTIKMVKEHLRNVPNKGIGYGILKYLTRDSEIAKGAASPILFNYLGQLDEDINSGEFSSSHL
SPGEAAGKGITREHPLEINAVVFRGKLAIQTTYNTRAYSEDVVRAFAQNYKEALKAVIRHCAEREETEKTPSDYgGDKGISLDQLEEIKLKYKGMEIEKIYPLANMQRGMLFHALEDKESQA
YFEQMAINMKGLIDERLFAETFNDIMERHEILRASIEYEITDEPRNVIIKDRKINLDYHDLRKQSPAEREQVIQAYRKADREKGFRLNSEPLIRAALMRTEDDSYTFIWTNHHILLDGWSRGIIM
GELFHMYHMKEARQKHRLEEARPYSDYIGWLQQQDKEAAKAYWRNYLSGFTEKSPISVLAGSSGHAKYKRKEAVIEFPEQLTGRITELASRNNVTFHTVLQCIWGMLLARYNQTDDVVF
GTVISGRDAQVTGIEKMVGLFINTVPTRIRLDKSQSFKELIKSVQEQALEGRTYHDMNLSEVQSLSELKRELLDHILIFENYAVDQSAFETSGKRGAGFVFEEIHAEEQTNYGFNIVAVPGER
LVIKLTYDGNIYHDHIIAGIKGHLQQVMEQVVQHEDQSLNDITVLSEAERNRLLYEWNDTKAEYPNQTIHRLFEEQAEKTPELAAVVSGNDKLTYRELNEKSNQLARYLRDKGVKADTIVAIM
AERSPEMVVGIMGILKAGGAYLPIDPDYPEERIKYMLEDSGAAIILADHKQDLGTLHQEAVELTGDFSSYPADNLEPAGNADSLAYIIYTSGSTGKPKGVMIRQRGLVNYITWADRVYVQGE
QLDFALYSSIAFDLTVTSIFTPLISGNRVIVYRHSEDGEPLIRKVFRDQKAGIVKLTPSHLSLVKDMDASGSSIKRLIVGGEDLKTELAKEITERFHHNIEIYNEYGPTETVVGCMIYQYDAGWD
RQVSVPIGKPASNVQLYILDERQEVQPVGIAGELYISGDGVAKGYLNKPELTSEKFLPNPFLPGERMYRTGDLAKMRPDGHIEYLGRIDHQVKIRGYRIELGEIEHQLLRHSDIKEAAVAAKT
DQNNDQVLCAYVVSERDITQKDIKTFLAKELPEYMVPSYLLKLDELPLTPNGKVDLKALPEPDRSAGALLEYEPPRHELEEKMAAIWEDILNIEQIGINANIFDIGANSLNVMSFVSRLYAELG
FRVPFKDIFSKPTIKELSDFLKHAQDLLKDYTDDCMQLTRAEEGGKNLFCFPPAASMGIAYMGLAKHLKQHSVYSFNFIPSANRIRKYADIIKNIQGEGPYTLIGYSSGGILAFDVAKELNRQG
YEVEDLIIIDSKYRTKAEKHQFTEEEYREEISKTFELEKYRDVEKLLSDYLVDLVMKSYVYIQNTVTTGAIDGHISYIKSSDNQRGENMMMWEKATSKTFTVVQGAGTHMQMISKSHPDILER
NARLIHDIINKTVKIGSRSHHHHHH

14) BacC4-C5[A-PCP-E-COM(P5063A)-C-A-PCP-TE]-H$_6$

MDSDEEMNALLDQNQGKQADYPQDQTVHQLFEQQADKTPEQTAVVYADEKLTYRELNERANQLARLLRDKGADADQPVAIMIEPSLEMIISMLAVLKAGAAYVPIEPEQLTKRTNEILSDS
RAAILLVKGSVKENVAFAGEIVNVADGLIDAKVASNLSASGSADQNAYIIYTSGSTGKPKGVFVRHGNVVNYTTWFMKEAGLTENDKAMLVSSYAFDLGYTSIFSALLSGSELHIARKECYT
NAHRALKYIKENGITYIKLTPSLFNIFVNDPGFSAEKPCATLRLVVLGGEMINTRDVETFYNQYPDHVVMNHYGPTETTIGSVFKVIDPEHLDSFKECPVIGTPIHNTNAYVLDENMKLLPEGV
YGELCIAGAGVTGGYVNRPDETKEKFIENPFAPHTKMYRTGDLARRLSDGNIELAGRIDTQVKVRGYRIEPEEIKNRLLAHDDIKEAFIAAREDHKGAKQLCAYFTADAELPFEDIRTYLMHE
LPEYMIPSSFVQIEKMPLSANGKIDTAALPEPQPGKETEYEPPRNETEEKLVQIWEEVLGIDKIGITHHFFAAGGDSIKALQMISRLSREGLSLEMKDLFANPQIKSLSRYVKAESDKSASYET
VEGEVLLTPIQQEYFSLNKTDRNHYNHAVMLYRKNGFDESIVKRVFKEIIKHHDALRTVFTEEDGKIIQYNRGPDKQLFDLFVYDVSSENDQPQKVYQLATELQQSIDIETGPLVKLALFKTN
NGDHLLIIIHHLVVDGISWRILFEDLAIGYSQLANGEKVEFYPKTASYQAYARHIAEYAKSVKLLSEKQYWLKAIAEGVEFLDMNENAGAFKVEDSRTFSTELEKEETKRLLRETNRAYHTEIN
DILITALLVAARDMNGQNQLRITLEGHGREQVADGIDISRTVGWFTSKYPVFIDLGQETDMSRTIKMVKEHLRNVPNKGIGYGILKYLTRDSEIAKGAASPILFNYLGQLDEDINSGEFSSSHL
SPGEAAGKGITREHPLEINAVVFRGKLAIQTTYNTRAYSEDVVRAFAQNYKEALKAVIRHCAEREETEKTASDYGDKGISLDQLEEIKLKYKGMEIEKIYPLANMQRGMLFHALEDKESQAY
FEQMAINMKGLIDERLFAETFNDIMERHEILRASIEYEITDEPRNVIIKDRKINLDYHDLRKQSPAEREQVIQAYRKADREKGFRLNSEPLIRAALMRTEDDSYTFIWTNHHILLDGWSRGIIMG
ELFHMYHMKEARQKHRLEEARPYSDYIGWLQQQDKEAAKAYWRNYLSGFTEKSPISVLAGSSGHAKYKRKEAVIEFPEQLTGRITELASRNNVTFHTVLQCIWGMLLARYNQTDDVVFG
TVISGRDAQVTGIEKMVGLFINTVPTRIRLDKSQSFKELIKSVQEQALEGRTYHDMNLSEVQSLSELKRELLDHILIFENYAVDQSAFETSGKRGAGFVFEEIHAEEQTNYGFNIVAVPGERL
VIKLTYDGNIYHDHIIAGIKGHLQQVMEQVVQHEDQSLNDITVLSEAERNRLLYEWNDTKAEYPNQTIHRLFEEQAEKTPELAAVVSGNDKLTYRELNEKSNQLARYLRDKGVKADTIVAIMA
ERSPEMVVGIMGILKAGGAYLPIDPDYPEERIKYMLEDSGAAIILADHKQDLGTLHQEAVELTGDFSSYPADNLEPAGNADSLAYIIYTSGSTGKPKGVMIRQRGLVNYITWADRVYVQGEQ
LDFALYSSIAFDLTVTSIFTPLISGNRVIVYRHSEDGEPLIRKVFRDQKAGIVKLTPSHLSLVKDMDASGSSIKRLIVGGEDLKTELAKEITERFHHNIEIYNEYGPTETVVGCMIYQYDAGWDR
QVSVPIGKPASNVQLYILDERQEVQPVGIAGELYISGDGVAKGYLNKPELTSEKFLPNPFLPGERMYRTGDLAKMRPDGHIEYLGRIDHQVKIRGYRIELGEIEHQLLRHSDIKEAAVAAKTD
QNNDQVLCAYVVSERDITQKDIKTFLAKELPEYMVPSYLLKLDELPLTPNGKVDLKALPEPDRSAGALLEYEPPRHELEEKMAAIWEDILNIEQIGINANIFDIGANSLNVMSFVSRLYAELGF
RVPFKDIFSKPTIKELSDFLKHAQDLLKDYTDDCMQLTRAEEGGKNLFCFPPAASMGIAYMGLAKHLKQHSVYSFNFIPSANRIRKYADIIKNIQGEGPYTLIGYSSGGILAFDVAKELNRQG
YEVEDLIIIDSKYRTKAEKHQFTEEEYREEISKTFELEKYRDVEKLLSDYLVDLVMKSYVYIQNTVTTGAIDGHISYIKSSDNQRGENMMMWEKATSKTFTVVQGAGTHMQMISKSHPDILER
NARLIHDIINKTVKIGSRSHHHHHH

15) BacC4-C5[A-PCP-E-COM(Ins5086GGS)-C-A-PCP-TE]-H$_6$

MDSDEEMNALLDQNQGKQADYPQDQTVHQLFEQQADKTPEQTAVVYADEKLTYRELNERANQLARLLRDKGADADQPVAIMIEPSLEMIISMLAVLKAGAAYVPIEPEQLAKRTNEILSDS
RAAILLVKGSVKENVAFAGEIVNVADGLIDAKVASNLSASGSADQNAYIIYTSGSTGKPKGVFVRHGNVVNYTTWFMKEAGLTENDKAMLVSSYAFDLGYTSIFSALLSGSELHIARKECYT
NAHRALKYIKENGITYIKLTPSLFNIFVNDPGFSAEKPCATLRLVVLGGEMINTRDVETFYNQYPDHVVMNHYGPTETTIGSVFKVIDPEHLDSFKECPVIGTPIHNTNAYVLDENMKLLPEGV
YGELCIAGAGVTGGYVNRPDETKEKFIENPFAPHTKMYRTGDLARRLSDGNIELAGRIDTQVKVRGYRIEPEEIKNRLLAHDDIKEAFIAAREDHKGAKQLCAYFTADAELPFEDIRTYLMHE
LPEYMIPSSFVQIEKMPLSANGKIDTAALPEPQPGKETEYEPPRNETEEKLVQIWEEVLGIDKIGITHHFFAAGGDSIKALQMISRLSREGLSLEMKDLFANPQIKSLSRYVKAESDKSASYET
VEGEVLLTPIQQEYFSLNKTDRNHYNHAVMLYRKNGFDESIVKRVFKEIIKHHDALRTVFTEEDGKIIQYNRGPDKQLFDLFVYDVSSENDQPQKVYQLATELQQSIDIETGPLVKLALFKTN
NGDHLLIIIHHLVVDGISWRILFEDLAIGYSQLANGEKVEFYPKTASYQAYARHIAEYAKSVKLLSEKQYWLKAIAEGVEFLDMNENAGAFKVEDSRTFSTELEKEETKRLLRETNRAYHTEIN
DILITALLVAARDMNGQNQLRITLEGHGREQVADGIDISRTVGWFTSKYPVFIDLGQETDMSRTIKMVKEHLRNVPNKGIGYGILKYLTRDSEIAKGAASPILFNYLGQLDEDINSGEFSSSHL
SPGEAAGKGITREHPLEINAVVFRGKLAIQTTYNTRAYSEDVVRAFAQNYKEALKAVIRHCAEREETEKTPSDYGDKGISLDQLEEIKLKYKGggsMEIEKIYPLANMQRGMLFHALEDKESQ
AYFEQMAINMKGLIDERLFAETFNDIMERHEILRASIEYEITDEPRNVIIKDRKINLDYHDLRKQSPAEREQVIQAYRKADREKGFRLNSEPLIRAALMRTEDDSYTFIWTNHHILLDGWSRGII
MGELFHMYHMKEARQKHRLEEARPYSDYIGWLQQQDKEAAKAYWRNYLSGFTEKSPISVLAGSSGHAKYKRKEAVIEFPEQLTGRITELASRNNVTFHTVLQCIWGMLLARYNQTDDVV
FGTVISGRDAQVTGIEKMVGLFINTVPTRIRLDKSQSFKELIKSVQEQALEGRTYHDMNLSEVQSLSELKRELLDHILIFENYAVDQSAFETSGKRGAGFVFEEIHAEEQTNYGFNIVAVPGE
RLVIKLTYDGNIYHDHIIAGIKGHLQQVMEQVVQHEDQSLNDITVLSEAERNRLLYEWNDTKAEYPNQTIHRLFEEQAEKTPELAAVVSGNDKLTYRELNEKSNQLARYLRDKGVKADTIVAI
MAERSPEMVVGIMGILKAGGAYLPIDPDYPEERIKYMLEDSGAAIILADHKQDLGTLHQEAVELTGDFSSYPADNLEPAGNADSLAYIIYTSGSTGKPKGVMIRQRGLVNYITWADRVYVQG
EQLDFALYSSIAFDLTVTSIFTPLISGNRVIVYRHSEDGEPLIRKVFRDQKAGIVKLTPSHLSLVKDMDASGSSIKRLIVGGEDLKTELAKEITERFHHNIEIYNEYGPTETVVGCMIYQYDAGW
DRQVSVPIGKPASNVQLYILDERQEVQPVGIAGELYISGDGVAKGYLNKPELTSEKFLPNPFLPGERMYRTGDLAKMRPDGHIEYLGRIDHQVKIRGYRIELGEIEHQLLRHSDIKEAAVAAK
TDQNNDQVLCAYVVSERDITQKDIKTFLAKELPEYMVPSYLLKLDELPLTPNGKVDLKALPEPDRSAGALLEYEPPRHELEEKMAAIWEDILNIEQIGINANIFDIGANSLNVMSFVSRLYAEL
GFRVPFKDIFSKPTIKELSDFLKHAQDLLKDYTDDCMQLTRAEEGGKNLFCFPPAASMGIAYMGLAKHLKQHSVYSFNFIPSANRIRKYADIIKNIQGEGPYTLIGYSSGGILAFDVAKELNRQ
GYEVEDLIIIDSKYRTKAEKHQFTEEEYREEISKTFELEKYRDVEKLLSDYLVDLVMKSYVYIQNTVTTGAIDGHISYIKSSDNQRGENMMMWEKATSKTFTVVQGAGTHMQMISKSHPDILE
RNARLIHDIINKTVKIGSRS**HHHHHH**

16) SBP-TycA[A-PCP-E-COM^D]

MDEKTTGWRGGHVVEGLAGELEQLRARLEHHPQGQREPMVANQANLIDNKRELEQHALVPYAQGKSIHQLFEEQAEAFPDRVAIVFENRRLSYQELNRKANQLARALLEKGVQTDSIV
GVMMEKSIENVIAILAVLKAGGAYVPIDIEYPRDRIQYILQDSQTKIVLTQKSVSQLVHDVGYSGEVVVLDEEQLDARETANLHQPSKPTDLAYVIYTSGTTGKPKGTMLEHKGIANLQSFFQ
NSFGVTEQDRIGLFASMSFDASVWEMFMALLSGASLYILSKQTIHDFAAFEHYLSENELTIITLPPTYLTHLTPERITSLRIMITAGSASSAPLVNKWKDKLRYINAYGPTETSICATIWEAPSN
QLSVQSVPIGKPIQNTHIYIVNEDLQLLPTGSEGELCIGGVGLARGYWNRPDLTAEKFVDNPFVPGEKMYRTGDLAKWLTDGTIEFLGRIDHQVKIRGHRIELGEIESVLLAHEHITEAVVIAR
EDQHAGQYLCAYYISQQEATPAQLRDYAAQKLPAYMLPSYFVKLDKMPLTPNDKIDRKALPEPDLTANQSQAAYHPPRTETESILVSIWQNVLGIEKIGIRDNFYSLGGDSIQAIQVVARLH
SYQLKLETKDLLNYPTIEQVALFVKSTTRKSDQGIIAGNVPLTPIQKWFFGKNFTNTGHWNQSSVLYRPEGFDPKVIQSVMDKIIEHHDALRMVYQHENGNVVQHNRGLGGQLYDFFSYNL
TAQPDVQQAIEAETQRLHSSMNLQEGPLVKVALFQTLHGDHLFLAIHHLVVDGISWRILFEDLATGYAQALAGQAISLPEKTDSFQSWSQWLQEYANEADLLSEIPYWESLESQAKNVSLP
KDYEVTDCKQKSVRNMRIRLHPEETEQLLKHANQAYQTEINDLLLAALGLAFAEWSKLAQIVIHLEGHGREDIIEQANVARTVGWFTSQYPVLLDLKQTAPLSDYIKLTKENMRKIPRKGIGY
DILKHVTLPENRGSLSFRVQPEVTFNYLGQFDADMRTELFTRSPYSGGNTLGADGKNNLSPESEVYTALNITGLIEGGELVLTFSYSSEQYREESIQQLSQSYQKHLLAIIAHCTEKKEVER
TPSDFSVKGLQMEEMDDIFELLANTLR

17) TycB1[COM^A-A-PCP]-His$_6$

MSVFSKEQVQDMYALTPMQEGMLFHALLDQEHNSHLVQMSISLQGDLDVGLFTDSLHVLVERYDVFRTLFLYEKLKQPLQVVLKQRPIPIEFYDLSACDESEKQLRYTQYKRADQERTFH
LAKDPLMRVALFQMSQHDYQVIWSFHHILMDGWCFSIIFDDLLAIYLSLQNKTALSLEPVQPYSRFINWLEKQNKQAALNYWSDYLEAYEQKTTLPKKEAAFAKAFQPTQYRFSLNRTLTK
QLGTIASQNQVTLSTVIQTIWGVLLQKYNAAHDVLFGSIVSGRPTDIVGIDKMVGLFINTIPFRVQAKAGQTFSELLQAVHKRTLQSQPYEHVPLYDIQTQSVLKQELIDHLLVIENYPLVEALQ

KKALNQQIGFTITAVEMFEPTNYDLTVMVMPKEELAFRFDYNAALFDEQVVQKLAGHLQQIADCVANNSGVELCQIPLLTEAETSQLLAKRTETAADYPAATMHELFSRQAEKTPEQVAVV
FADQHLTYRELDEKSNQLARFLRKKGIGTGSLVGTLLDRSLDMIVGILGVLKAGGAFVPIDPELPAERIAYMLTHSRVPLVVTQNHLRAKVTTPTETIDINTAVIGEESRAPIESLNQPHDLFYII
YTSGTTGQPKGVMLEHRNMANLMHFTFDQTNIAFHEKVLQYTTCSFDVCYQEIFSTLLSGGQLYLITNELRRHVEKLFAFIQEKQISILSLPVSFLKFIFNEQDYAQSFPRCVKHIITAGEQLV
VTHELQKYLRQHRVFLHNHYGPSETHVVTTCTMDPGQAIPELPPIGKPISNTGIYILDEGLQLKPEGIVGELYISGANVGRGYLHQPELTAEKFLDNPYQPGERMYRTGDLARWLPDGQLE
FLGRIDHQVKIRGHRIELGEIESRLLNHPAIKEAVVIDRADETGGKFLCAYVVLQKALSDEEMRAYLAQALPEYMIPSFFVTLERIPVTPNGKTDRRALPKPEGSAKTKADYVAPTTELEQKLV
AIWEQILGVSPIGIQDHFFTLGGHSLKAIQLISRIQKECQADVPLRVLFEQPTIQALAAYVEGSRS**HHHHHH**

18) SBP-TycA[A-PCP-E-COM^{D}(ΔHlx)]

MDEKTTGWRGGHVVEGLAGELEQLRARLEHHPQGQREPMVANQANLIDNKRELEQHALVPYAQGKSIHQLFEEQAEAFPDRVAIVFENRRLSYQELNRKANQLARALLEKGVQTDSIV
GVMMEKSIENVIAILAVLKAGGAYVPIDIEYPRDRIQYILQDSQTKIVLTQKSVSQLVHDVGYSGEVVVLDEEQLDARETANLHQPSKPTDLAYVIYTSGTTGKPKGTMLEHKGIANLQSFFQ
NSFGVTEQDRIGLFASMSFDASVWEMFMALLSGASLYILSKQTIHDFAAFEHYLSENELTIITLPPTYLTHLTPERITSLRIMITAGSASSAPLVNKWKDKLRYINAYGPTETSICATIWEAPSN
QLSVQSVPIGKPIQNTHIYIVNEDLQLLPTGSEGELCIGGVGLARGYWNRPDLTAEKFVDNPFVPGEKMYRTGDLAKWLTDGTIEFLGRIDHQVKIRGHRIELGEIESVLLAHEHITEAVVIAR
EDQHAGQYLCAYYISQQEATPAQLRDYAAQKLPAYMLPSYFVKLDKMPLTPNDKIDRKALPEPDLTANQSQAAYHPPRTETESILVSIWQNVLGIEKIGIRDNFYSLGGDSIQAIQVVARLH
SYQLKLETKDLLNYPTIEQVALFVKSTTRKSDQGIIAGNVPLTPIQKWFFGKNFTNTGHWNQSSVLYRPEGFDPKVIQSVMDKIIEHHDALRMVYQHENGNVVQHNRGLGGQLYDFFSYNL
TAQPDVQQAIEAETQRLHSSMNLQEGPLVKVALFQTLHGDHLFLAIHHLVVDGISWRILFEDLATGYAQALAGQAISLPEKTDSFQSWSQWLQEYANEADLLSEIPYWESLESQAKNVSLP
KDYEVTDCKQKSVRNMRIRLHPEETEQLLKHANQAYQTEINDLLLAALGLAFAEWSKLAQIVIHLEGHGREDIIEQANVARTVGWFTSQYPVLLDLKQTAPLSDYIKLTKENMRKIPRKGIGY
DILKHVTLPENRGSLSFRVQPEVTFNYLGQFDADMRTELFTRSPYSGGNTLGADGKNNLSPESEVYTALNITGLIEGGELVLTFSYSSEQYREESIQQLSQSYQKHLLAIIAHCTEKKEVER
TPSDFSVKGLQ

19) SBP-TycA[A-PCP-E-COM^{D}(ΔLinker)]

MDEKTTGWRGGHVVEGLAGELEQLRARLEHHPQGQREPMVANQANLIDNKRELEQHALVPYAQGKSIHQLFEEQAEAFPDRVAIVFENRRLSYQELNRKANQLARALLEKGVQTDSIV
GVMMEKSIENVIAILAVLKAGGAYVPIDIEYPRDRIQYILQDSQTKIVLTQKSVSQLVHDVGYSGEVVVLDEEQLDARETANLHQPSKPTDLAYVIYTSGTTGKPKGTMLEHKGIANLQSFFQ
NSFGVTEQDRIGLFASMSFDASVWEMFMALLSGASLYILSKQTIHDFAAFEHYLSENELTIITLPPTYLTHLTPERITSLRIMITAGSASSAPLVNKWKDKLRYINAYGPTETSICATIWEAPSN
QLSVQSVPIGKPIQNTHIYIVNEDLQLLPTGSEGELCIGGVGLARGYWNRPDLTAEKFVDNPFVPGEKMYRTGDLAKWLTDGTIEFLGRIDHQVKIRGHRIELGEIESVLLAHEHITEAVVIAR
EDQHAGQYLCAYYISQQEATPAQLRDYAAQKLPAYMLPSYFVKLDKMPLTPNDKIDRKALPEPDLTANQSQAAYHPPRTETESILVSIWQNVLGIEKIGIRDNFYSLGGDSIQAIQVVARLH
SYQLKLETKDLLNYPTIEQVALFVKSTTRKSDQGIIAGNVPLTPIQKWFFGKNFTNTGHWNQSSVLYRPEGFDPKVIQSVMDKIIEHHDALRMVYQHENGNVVQHNRGLGGQLYDFFSYNL
TAQPDVQQAIEAETQRLHSSMNLQEGPLVKVALFQTLHGDHLFLAIHHLVVDGISWRILFEDLATGYAQALAGQAISLPEKTDSFQSWSQWLQEYANEADLLSEIPYWESLESQAKNVSLP
KDYEVTDCKQKSVRNMRIRLHPEETEQLLKHANQAYQTEINDLLLAALGLAFAEWSKLAQIVIHLEGHGREDIIEQANVARTVGWFTSQYPVLLDLKQTAPLSDYIKLTKENMRKIPRKGIGY
DILKHVTLPENRGSLSFRVQPEVTFNYLGQFDADMRTELFTRSPYSGGNTLGADGKNNLSPESEVYTALNITGLIEGGELVLTFSYSSEQYREESIQQLSQSYQKHLLAIIAHCTEKKEGLQ
MEEMDDIFELLANTLR

20) SBP-TycA[A-PCP-E-COM^{D}(VER(GGS)$_3$TPSD)]

MDEKTTGWRGGHVVEGLAGELEQLRARLEHHPQGQREPMVANQANLIDNKRELEQHALVPYAQGKSIHQLFEEQAEAFPDRVAIVFENRRLSYQELNRKANQLARALLEKGVQTDSIV
GVMMEKSIENVIAILAVLKAGGAYVPIDIEYPRDRIQYILQDSQTKIVLTQKSVSQLVHDVGYSGEVVVLDEEQLDARETANLHQPSKPTDLAYVIYTSGTTGKPKGTMLEHKGIANLQSFFQ
NSFGVTEQDRIGLFASMSFDASVWEMFMALLSGASLYILSKQTIHDFAAFEHYLSENELTIITLPPTYLTHLTPERITSLRIMITAGSASSAPLVNKWKDKLRYINAYGPTETSICATIWEAPSN
QLSVQSVPIGKPIQNTHIYIVNEDLQLLPTGSEGELCIGGVGLARGYWNRPDLTAEKFVDNPFVPGEKMYRTGDLAKWLTDGTIEFLGRIDHQVKIRGHRIELGEIESVLLAHEHITEAVVIAR
EDQHAGQYLCAYYISQQEATPAQLRDYAAQKLPAYMLPSYFVKLDKMPLTPNDKIDRKALPEPDLTANQSQAAYHPPRTETESILVSIWQNVLGIEKIGIRDNFYSLGGDSIQAIQVVARLH
SYQLKLETKDLLNYPTIEQVALFVKSTTRKSDQGIIAGNVPLTPIQKWFFGKNFTNTGHWNQSSVLYRPEGFDPKVIQSVMDKIIEHHDALRMVYQHENGNVVQHNRGLGGQLYDFFSYNL
TAQPDVQQAIEAETQRLHSSMNLQEGPLVKVALFQTLHGDHLFLAIHHLVVDGISWRILFEDLATGYAQALAGQAISLPEKTDSFQSWSQWLQEYANEADLLSEIPYWESLESQAKNVSLP
KDYEVTDCKQKSVRNMRIRLHPEETEQLLKHANQAYQTEINDLLLAALGLAFAEWSKLAQIVIHLEGHGREDIIEQANVARTVGWFTSQYPVLLDLKQTAPLSDYIKLTKENMRKIPRKGIGY
DILKHVTLPENRGSLSFRVQPEVTFNYLGQFDADMRTELFTRSPYSGGNTLGADGKNNLSPESEVYTALNITGLIEGGELVLTFSYSSEQYREESIQQLSQSYQKHLLAIIAHCTEKKEVERg
gsggsggsTPSDFSVKGLQMEEMDDIFELLANTLR

21) SBP-TycA[A-PCP-E-COM^{D}(ΔLinker_sm)]

MDEKTTGWRGGHVVEGLAGELEQLRARLEHHPQGQREPMVANQANLIDNKRELEQHALVPYAQGKSIHQLFEEQAEAFPDRVAIVFENRRLSYQELNRKANQLARALLEKGVQTDSIV
GVMMEKSIENVIAILAVLKAGGAYVPIDIEYPRDRIQYILQDSQTKIVLTQKSVSQLVHDVGYSGEVVVLDEEQLDARETANLHQPSKPTDLAYVIYTSGTTGKPKGTMLEHKGIANLQSFFQ
NSFGVTEQDRIGLFASMSFDASVWEMFMALLSGASLYILSKQTIHDFAAFEHYLSENELTIITLPPTYLTHLTPERITSLRIMITAGSASSAPLVNKWKDKLRYINAYGPTETSICATIWEAPSN
QLSVQSVPIGKPIQNTHIYIVNEDLQLLPTGSEGELCIGGVGLARGYWNRPDLTAEKFVDNPFVPGEKMYRTGDLAKWLTDGTIEFLGRIDHQVKIRGHRIELGEIESVLLAHEHITEAVVIAR
EDQHAGQYLCAYYISQQEATPAQLRDYAAQKLPAYMLPSYFVKLDKMPLTPNDKIDRKALPEPDLTANQSQAAYHPPRTETESILVSIWQNVLGIEKIGIRDNFYSLGGDSIQAIQVVARLH
SYQLKLETKDLLNYPTIEQVALFVKSTTRKSDQGIIAGNVPLTPIQKWFFGKNFTNTGHWNQSSVLYRPEGFDPKVIQSVMDKIIEHHDALRMVYQHENGNVVQHNRGLGGQLYDFFSYNL
TAQPDVQQAIEAETQRLHSSMNLQEGPLVKVALFQTLHGDHLFLAIHHLVVDGISWRILFEDLATGYAQALAGQAISLPEKTDSFQSWSQWLQEYANEADLLSEIPYWESLESQAKNVSLP
KDYEVTDCKQKSVRNMRIRLHPEETEQLLKHANQAYQTEINDLLLAALGLAFAEWSKLAQIVIHLEGHGREDIIEQANVARTVGWFTSQYPVLLDLKQTAPLSDYIKLTKENMRKIPRKGIGY
DILKHVTLPENRGSLSFRVQPEVTFNYLGQFDADMRTELFTRSPYSGGNTLGADGKNNLSPESEVYTALNITGLIEGGELVLTFSYSSEQYREESIQQLSQSYQKHLLAIIAHCTEKKETPS
DFSVKGLQMEEMDDIFELLANTLR

22) SBP-TycA[A-PCP-E-COM^{D}(VERgTPSD)]

MDEKTTGWRGGHVVEGLAGELEQLRARLEHHPQGQREPMVANQANLIDNKRELEQHALVPYAQGKSIHQLFEEQAEAFPDRVAIVFENRRLSYQELNRKANQLARALLEKGVQTDSIV
GVMMEKSIENVIAILAVLKAGGAYVPIDIEYPRDRIQYILQDSQTKIVLTQKSVSQLVHDVGYSGEVVVLDEEQLDARETANLHQPSKPTDLAYVIYTSGTTGKPKGTMLEHKGIANLQSFFQ
NSFGVTEQDRIGLFASMSFDASVWEMFMALLSGASLYILSKQTIHDFAAFEHYLSENELTIITLPPTYLTHLTPERITSLRIMITAGSASSAPLVNKWKDKLRYINAYGPTETSICATIWEAPSN
QLSVQSVPIGKPIQNTHIYIVNEDLQLLPTGSEGELCIGGVGLARGYWNRPDLTAEKFVDNPFVPGEKMYRTGDLAKWLTDGTIEFLGRIDHQVKIRGHRIELGEIESVLLAHEHITEAVVIAR
EDQHAGQYLCAYYISQQEATPAQLRDYAAQKLPAYMLPSYFVKLDKMPLTPNDKIDRKALPEPDLTANQSQAAYHPPRTETESILVSIWQNVLGIEKIGIRDNFYSLGGDSIQAIQVVARLH
SYQLKLETKDLLNYPTIEQVALFVKSTTRKSDQGIIAGNVPLTPIQKWFFGKNFTNTGHWNQSSVLYRPEGFDPKVIQSVMDKIIEHHDALRMVYQHENGNVVQHNRGLGGQLYDFFSYNL
TAQPDVQQAIEAETQRLHSSMNLQEGPLVKVALFQTLHGDHLFLAIHHLVVDGISWRILFEDLATGYAQALAGQAISLPEKTDSFQSWSQWLQEYANEADLLSEIPYWESLESQAKNVSLP
KDYEVTDCKQKSVRNMRIRLHPEETEQLLKHANQAYQTEINDLLLAALGLAFAEWSKLAQIVIHLEGHGREDIIEQANVARTVGWFTSQYPVLLDLKQTAPLSDYIKLTKENMRKIPRKGIGY
DILKHVTLPENRGSLSFRVQPEVTFNYLGQFDADMRTELFTRSPYSGGNTLGADGKNNLSPESEVYTALNITGLIEGGELVLTFSYSSEQYREESIQQLSQSYQKHLLAIIAHCTEKKEVERg
TPSDFSVKGLQMEEMDDIFELLANTLR

23) SBP-TycA[A-PCP-E-COM^{D}(ΔV1059)]

MDEKTTGWRGGHVVEGLAGELEQLRARLEHHPQGQREPMVANQANLIDNKRELEQHALVPYAQGKSIHQLFEEQAEAFPDRVAIVFENRRLSYQELNRKANQLARALLEKGVQTDSIV
GVMMEKSIENVIAILAVLKAGGAYVPIDIEYPRDRIQYILQDSQTKIVLTQKSVSQLVHDVGYSGEVVVLDEEQLDARETANLHQPSKPTDLAYVIYTSGTTGKPKGTMLEHKGIANLQSFFQ
NSFGVTEQDRIGLFASMSFDASVWEMFMALLSGASLYILSKQTIHDFAAFEHYLSENELTIITLPPTYLTHLTPERITSLRIMITAGSASSAPLVNKWKDKLRYINAYGPTETSICATIWEAPSN
QLSVQSVPIGKPIQNTHIYIVNEDLQLLPTGSEGELCIGGVGLARGYWNRPDLTAEKFVDNPFVPGEKMYRTGDLAKWLTDGTIEFLGRIDHQVKIRGHRIELGEIESVLLAHEHITEAVVIAR
EDQHAGQYLCAYYISQQEATPAQLRDYAAQKLPAYMLPSYFVKLDKMPLTPNDKIDRKALPEPDLTANQSQAAYHPPRTETESILVSIWQNVLGIEKIGIRDNFYSLGGDSIQAIQVVARLH
SYQLKLETKDLLNYPTIEQVALFVKSTTRKSDQGIIAGNVPLTPIQKWFFGKNFTNTGHWNQSSVLYRPEGFDPKVIQSVMDKIIEHHDALRMVYQHENGNVVQHNRGLGGQLYDFFSYNL
TAQPDVQQAIEAETQRLHSSMNLQEGPLVKVALFQTLHGDHLFLAIHHLVVDGISWRILFEDLATGYAQALAGQAISLPEKTDSFQSWSQWLQEYANEADLLSEIPYWESLESQAKNVSLP
KDYEVTDCKQKSVRNMRIRLHPEETEQLLKHANQAYQTEINDLLLAALGLAFAEWSKLAQIVIHLEGHGREDIIEQANVARTVGWFTSQYPVLLDLKQTAPLSDYIKLTKENMRKIPRKGIGY
DILKHVTLPENRGSLSFRVQPEVTFNYLGQFDADMRTELFTRSPYSGGNTLGADGKNNLSPESEVYTALNITGLIEGGELVLTFSYSSEQYREESIQQLSQSYQKHLLAIIAHCTEKKEERT
PSDFSVKGLQMEEMDDIFELLANTLR

24) SBP-TycA[A-PCP-E-COM^{D}(P1063A)]

MDEKTTGWRGGHVVEGLAGELEQLRARLEHHPQGQREPMVANQANLIDNKRELEQHALVPYAQGKSIHQLFEEQAEAFPDRVAIVFENRRLSYQELNRKANQLARALLEKGVQTDSIV
GVMMEKSIENVIAILAVLKAGGAYVPIDIEYPRDRIQYILQDSQTKIVLTQKSVSQLVHDVGYSGEVVVLDEEQLDARETANLHQPSKPTDLAYVIYTSGTTGKPKGTMLEHKGIANLQSFFQ
NSFGVTEQDRIGLFASMSFDASVWEMFMALLSGASLYILSKQTIHDFAAFEHYLSENELTIITLPPTYLTHLTPERITSLRIMITAGSASSAPLVNKWKDKLRYINAYGPTETSICATIWEAPSN
QLSVQSVPIGKPIQNTHIYIVNEDLQLLPTGSEGELCIGGVGLARGYWNRPDLTAEKFVDNPFVPGEKMYRTGDLAKWLTDGTIEFLGRIDHQVKIRGHRIELGEIESVLLAHEHITEAVVIAR
EDQHAGQYLCAYYISQQEATPAQLRDYAAQKLPAYMLPSYFVKLDKMPLTPNDKIDRKALPEPDLTANQSQAAYHPPRTETESILVSIWQNVLGIEKIGIRDNFYSLGGDSIQAIQVVARLH
SYQLKLETKDLLNYPTIEQVALFVKSTTRKSDQGIIAGNVPLTPIQKWFFGKNFTNTGHWNQSSVLYRPEGFDPKVIQSVMDKIIEHHDALRMVYQHENGNVVQHNRGLGGQLYDFFSYNL
TAQPDVQQAIEAETQRLHSSMNLQEGPLVKVALFQTLHGDHLFLAIHHLVVDGISWRILFEDLATGYAQALAGQAISLPEKTDSFQSWSQWLQEYANEADLLSEIPYWESLESQAKNVSLP
KDYEVTDCKQKSVRNMRIRLHPEETEQLLKHANQAYQTEINDLLLAALGLAFAEWSKLAQIVIHLEGHGREDIIEQANVARTVGWFTSQYPVLLDLKQTAPLSDYIKLTKENMRKIPRKGIGY

DILKHVTLPENRGSLSFRVQPEVTFNYLGQFDADMRTELFTRSPYSGGNTLGADGKNNLSPESEVYTALNITGLIEGGELVLTFSYSSEQYREESIQQLSQSYQKHLLAIIAHCTEKKEVER
TASDFSVKGLQMEEMDDIFELLANTLR

25) TycB1[COM^{A}(S5Bpa)-A-PCP]

SVFBpaKEQVQDMYALTPMQEGMLFHALLDQEHNSHLVQMSISLQGDLDVGLFTDSLHVLVERYDVFRTLFLYEKLKQPLQVVLKQRPIPIEFYDLSACDESEKQLRYTQYKRADQERTFH
LAKDPLMRVALFQMSQHDYQVIWSFHHILMDGWCFSIIFDDLLAIYLSLQNKTALSLEPVQPYSRFINWLEKQNKQAALNYWSDYLEAYEQKTTLPKKEAAFAKAFQPTQYRFSLNRTLTK
QLGTIASQNQVTLSTVIQTIWGVLLQKYNAAHDVLFGSIVSGRPTDIVGIDKMVGLFINTIPFRVQAKAGQTFSELLQAVHKRTLQSQPYEHVPLYDIQTQSVLKQELIDHLLVIENYPLVEALQ
KKALNQQIGFTITAVEMFEPTNYDLTVMVMPKEELAFRFDYNAALFDEQVVQKLAGHLQQIADCVANNSGVELCQIPLLTEAETSQLLAKRTETAADYPAATMHELFSRQAEKTPEQVAVV
FADQHLTYRELDEKSNQLARFLRKKGIGTGSLVGTLLDRSLDMIVGILGVLKAGGAFVPIDPELPAERIAYMLTHSRVPLVVTQNHLRAKVTTPTETIDINTAVIGEESRAPIESLNQPHDLFYII
YTSGTTGQPKGVMLEHRNMANLMHFTFDQTNIAFHEKVLQYTTCSFDVCYQEIFSTLLSGGQLYLITNELRRHVEKLFAFIQEKQISILSLPVSFLKFIFNEQDYAQSFPRCVKHIITAGEQLV
VTHELQKYLRQHRVFLHNHYGPSETHVVTTCTMDPGQAIPELPPIGKPISNTGIYILDEGLQLKPEGIVGELYISGANVGRGYLHQPELTAEKFLDNPYQPGERMYRTGDLARWLPDGQLE
FLGRIDHQVKIRGHRIELGEIESRLLNHPAIKEAVVIDRADETGGKFLCAYVVLQKALSDEEMRAYLAQALPEYMIPSFFVTLERIPVTPNGKTDRRALPKPEGSAKTKADYVAPTTELEQKLV
AIWEQILGVSPIGIQDHFFTLGGHSLKAIQLISRIQKECQADVPLRVLFEQPTIQALAAYVEGSRSHHHHHH

Supporting References

[1] E. Dehling, J. Rüschenbaum, J. Diecker, W. Dörner, H. D. Mootz, *Chem Sci*, **2020**, *11*, 8945-8954.

[2] J. Rüschenbaum, W. Steinchen, F. Mayerthaler, A. L. Feldberg, H. D. Mootz, *Angew Chem Int Ed Engl*, **2022**, *61*, e202212994.

[3] J. W. Chin, A. B. Martin, D. S. King, L. Wang, P. G. Schultz, *Proc Natl Acad Sci U S A*, **2002**, *99*, 11020-11024.

[4] F. Gorrec, *J Appl Crystallogr*, **2009**, *42*, 1035-1042.

[5] K. M. Sparta, M. Krug, U. Heinemann, U. Mueller, M. S. Weiss, *J. Appl. Cryst.*, **2016**, *49*, 1085-1092.

[6] D. Liebschner, P. V. Afonine, M. L. Baker, G. Bunkoczi, V. B. Chen, T. I. Croll, B. Hintze, L. W. Hung, S. Jain, A. J. McCoy, N. W. Moriarty, R. D. Oeffner, B. K. Poon, M. G. Prisant, R. J. Read, J. S. Richardson, D. C. Richardson, M. D. Sammito, O. V. Sobolev, D. H. Stockwell, T. C. Terwilliger, A. G. Urzhumtsev, L. L. Videau, C. J. Williams, P. D. Adams, *Acta Crystallogr D Struct Biol*, **2019**, *75*, 861-877.

[7] T. C. Terwilliger, P. D. Adams, R. J. Read, A. J. McCoy, N. W. Moriarty, R. W. Grosse-Kunstleve, P. V. Afonine, P. H. Zwart, L. W. Hung, *Acta Crystallogr D Biol Crystallogr*, **2009**, *65*, 582-601.

[8] T. C. Terwilliger, R. W. Grosse-Kunstleve, P. V. Afonine, N. W. Moriarty, P. H. Zwart, L. W. Hung, R. J. Read, P. D. Adams, *Acta Crystallogr D Biol Crystallogr*, **2008**, *64*, 61-69.

[9] P. Emsley, B. Lohkamp, W. G. Scott, K. Cowtan, *Acta Crystallogr D Biol Crystallogr*, **2010**, *66*, 486-501.

[10] P. V. Afonine, R. W. Grosse-Kunstleve, N. Echols, J. J. Headd, N. W. Moriarty, M. Mustyakimov, T. C. Terwilliger, A. Urzhumtsev, P. H. Zwart, P. D. Adams, *Acta Crystallogr D Biol Crystallogr*, **2012**, *68*, 352-367.

[11] E. Dehling, G. Volkmann, J. C. Matern, W. Dörner, J. Alfermann, J. Diecker, H. D. Mootz, *J Mol Biol*, **2016**, *428*, 4345-4360.

[12] M. Götze, J. Pettelkau, S. Schaks, K. Bosse, C. H. Ihling, F. Krauth, R. Fritzsche, U. Kuhn, A. Sinz, *J Am Soc Mass Spectrom*, **2012**, *23*, 76-87.

[13] G. E. Crooks, G. Hon, J. M. Chandonia, S. E. Brenner, *Genome Res*, **2004**, *14*, 1188-1190.

[14] G. W. Heberlig, J. J. La Clair, M. D. Burkart, *Nature*, **2025**, *638*, 261-269.
